# Novel Heteroleptic Iridium(III) Complexes Containing COUBPY Ligands for Effective Photoinduction of Ferroptosis for Cancer Therapy

**DOI:** 10.1021/jacsau.5c01441

**Published:** 2025-12-20

**Authors:** Pezhman Ashoo, Alba Hernández-García, Eduardo Izquierdo-García, Neus Santiago, Rebeca Mondaray-Marín, Diego Abad-Montero, Manel Bosch, Neus Isidro, Valentin V. Novikov, Josep Rocas, María Dolores Santana, José Ruiz, Vicente Marchán

**Affiliations:** † Departamento de Química Inorgánica, Universidad de Murcia, and Murcia BioHealth Research Institute (IMIB-Arrixaca), Murcia E-30100, Spain; ‡ Departament de Química Inorgànica i Orgànica, Secció de Química Orgànica, 16724 Universitat de Barcelona (UB), Institut de Biomedicina de la Universitat de Barcelona (IBUB), Barcelona E-08028, Spain; § Ecopol Tech S.L., Nanobiotechnological Polymers Division, R&D Department, L’Arboç Del Penedès, Tarragona E-43720, Spain; ∥ Unitat de Microscòpia Òptica Avançada, Centres Científics i Tecnològics, Universitat de Barcelona, Barcelona E-08028, Spain; ⊥ Departament de Química Inorgànica i Orgànica, Secció de Química Inorgànica, Universitat de Barcelona (UB) and Institute of Nanoscience and Nanotechnology of the University of Barcelona (IN2UB), Martí i Franquès 1-11, Barcelona E-08028, Spain; # Professor Serra Húnter, Universitat de Barcelona, Barcelona E-08028, Spain

**Keywords:** iridium metallodrugs, coumarin, COUBPY, ferroptosis, photodynamic therapy, photosensitizer, nanoencapsulation

## Abstract

Ferroptosis, a recently described form of regulated, nonapoptotic cell death mechanism, presents significant potential for cancer treatment, particularly when combined with photodynamic therapy (PDT). In this study, we report the synthesis and biological evaluation of a series of Ir-COUBPY complexes as novel photosensitizers (PSs) for effective cancer phototherapy. These complexes exhibit high stability under both dark and light conditions and are capable of photogenerating Type I and Type II reactive oxygen species (ROS), as well as photo-oxidizing NADH. Electron paramagnetic resonance (EPR) spectroscopy provided direct evidence of light-induced superoxide and singlet oxygen generation, confirming dual ROS pathways. Moreover, the Ir-COUBPY complexes preferentially accumulated in the mitochondria of cancer cells, leading to the photogeneration of hydroxyl radicals and hydrogen peroxide. Photocytotoxicity studies on HeLa and A375 cancer cells underscored the role of the COUBPY ligand in enhancing PDT efficiency upon irradiation with both green and red light. Among the Ir-COUBPY complexes, the most effective PS, **Ir4a**, was encapsulated in polyurethane–polyurea hybrid nanocapsules (**NC-Ir4a**), resulting in a significant increase in phototoxic index values (e.g., from 64 to 179.6 in A375 cells). Mechanistic studies confirmed ferroptosis as the primary cell death pathway induced by **Ir4a**, supported by light-dependent lipid peroxidation, glutathione oxidation and depletion, intracellular ATP photodepletion, and the viability-restoring effect of Fer-1. These effects were more pronounced upon nanoencapsulation. Photobiological studies with 3D tumor spheroids of A375 cells further confirmed higher cellular uptake of **NC-Ir4a**, contributing to improved phototoxic efficiency. Overall, these findings highlight the potential of coumarin-based COUBPY ligands in the design of new Ir­(III)-based PSs that can be activated with light within the phototherapeutic window, operating through nonconventional cell death mechanisms such as ferroptosis.

## Graphical abstract

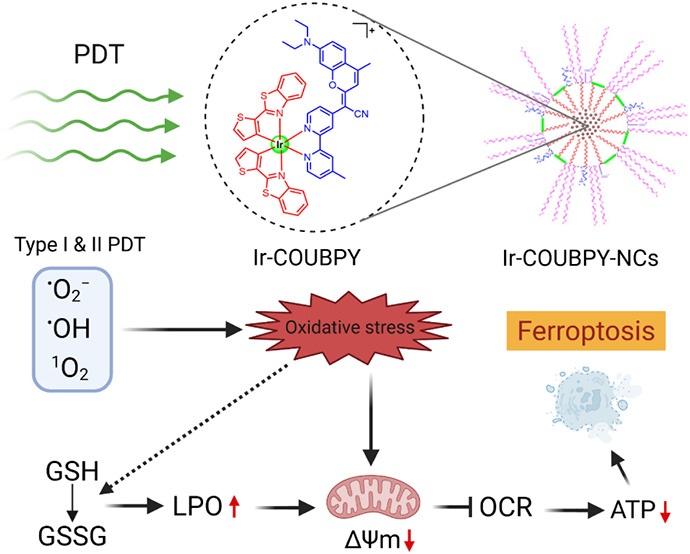

## Introduction

Cancer remains a leading cause of morbidity and mortality worldwide, underscoring the need to develop more effective and selective treatment modalities. Despite advances in conventional therapies such as chemotherapy, radiotherapy and immunotherapy, many types of cancer still exhibit resistance to treatment, often due to the heterogeneity of the tumor microenvironment. Additionally, cancer cells have the ability to evade apoptosis, which allows them to survive and continue growing despite treatment.^1,2^ As a result, alternative cell death mechanisms have garnered increasing attention in the search for novel therapeutic strategies. One such emerging mechanism is ferroptosis, a regulated, iron-dependent, and nonapoptotic form of cell death.

Ferroptosis is mediated by the accumulation of reactive oxygen species (ROS) through the Fenton reaction, which leads to phospholipid peroxidation within cellular membranes.^3,4^ This process is primarily regulated by glutathione peroxidase 4 (GPX4) and glutathione (GSH), which work together to reduce lipid peroxidation, thereby inhibiting ferroptosis and protecting cells from this form of cell death. Inhibition of GPX4 activity leads to the accumulation of lipid peroxides, ultimately compromising membrane integrity and triggering ferroptotic cell death.^5^ Compared to normal cells, cancer cells exhibit a modified intracellular redox state, characterized by elevated levels of antioxidants such as GSH, which can be up to 4–10 times higher. This high reductive intracellular environment allows cancer cells to resist oxidative stress, but it also makes them susceptible to ferroptosis when GSH levels are depleted. Intracellular ferric iron (Fe^3+^) can contribute to the downregulation of GSH, while ferrous iron (Fe^2+^) reacts with hydrogen peroxide via the Fenton reaction, generating hydroxyl radicals (^•^OH) that amplify oxidative damage and promote ferroptosis-mediated cell death.^6–9^ This vulnerability can be exploited by strategies that enhance ROS production, deplete the body’s antioxidant defenses, or disrupt iron homeostasis. Recent studies have demonstrated that iridium complexes can facilitate ferroptosis by promoting ROS accumulation and lipid peroxidation while simultaneously disrupting mitochondrial function.^10–13^

Photodynamic therapy (PDT) is an approved medical treatment modality that offers high spatial and temporal precision with minimal invasiveness,^14–17^ resulting in much milder and more localized side effects compared to conventional treatments. The typical PDT procedure involves activating a nontoxic photosensitive drug, known as a photosensitizer (PS), within the tumor using light of the appropriate wavelength. Upon light excitation followed by a spin flip, the PS is promoted to a triplet excited state (^3^ES) that can participate in electron (Type I PDT) and/or energy (Type II PDT) transfer reactions with O_2_ molecules and other substrates present in the irradiated tissues.^18^ This leads to the production of cytotoxic ROS, including singlet oxygen (^1^O_2_), superoxide anion radical (^•^O_2_^–^), hydrogen peroxide (H_2_O_2_), and hydroxyl radical.^19,20^ ROS can interact with nearby biomolecules (e.g., DNA/RNA, proteins, and lipids), as well as with subcellular organelles, causing damage and destruction of the tumor and/or tumor vasculature. Since PDT offers precise control over ROS production at the tumor site, it enables the selective eradication of malignant tumors while minimizing damage to surrounding normal tissues.^21^ Among the two PDT mechanisms, the Type II mechanism, which relies on the generation of ^1^O_2_, faces an inherent limitation due to its dependence on oxygen as a coreagent. In this context, the photogeneration of Type I ROS not only enhances PDT’s effectiveness under hypoxic conditions but can also induce ferroptosis, which is particularly effective in tumors with imbalanced redox homeostasis.^15,22,23^ Therefore, combining PDT and ferroptosis is a promising strategy in cancer treatment due to PDT’s ability to generate various types of ROS.^24,25^

Organic-based PSs, such as Photofrin, Levulan and Metvix, have been approved for treating various types of cancer and skin indications, including nonmelanoma skin cancer (NMSC), head and neck cancer, and bladder cancer, among others.^26^ However, these PSs, primarily based on tetrapyrrolic scaffolds, exhibit several limitations. These include complex and time-consuming synthesis and purification processes, poor aqueous solubility, rapid photobleaching or photodegradation, photosensitivity, and slow clearance from the body. In contrast to organic-based PSs, light absorption by transition metal complexes (TMCs) typically leads to efficient population of ^3^ES due to the “heavy atom effect”, which promotes intersystem crossing (ISC), i.e., triplet state conversion from the initially populated photoexcited singlet state. Furthermore, the structural versatility and synthetic tunability of TMCs allow for precise control over their photophysical and photochemical properties. This is achieved through careful selection of metal centers and ligands, which also influence their biological activity. These properties have been harnessed to develop light-activated drugs based on TMCs for PDT applications.^27–36^ Among them, Ru­(II) polypyridyl complexes hold significant potential to address some of the limitations of current PSs.^37,38^ A notable example is TLD-1433, which is currently in phase II clinical trials for the intravesical treatment of nonmuscle-invasive bladder cancer (NMIBC) using green light (NCT03945162).^39^

Cyclometalated iridium­(III) complexes with the structure [Ir­(C^N)_2_(N^N)]^+^ (where C^N represents a cyclometalated ligand and N^N a diimine ligand) have also been widely investigated as luminescent and photosensitizing materials.^40–43^ This is due to their remarkable photostability, long triplet excited state lifetimes, and high quantum yields for both emission and ROS production. Although these PSs show great promise for cancer treatment, their relatively low molar absorption coefficients at long wavelengths make them ineffective for treating large solid hypoxic tumors. This limitation significantly hampers their clinical application.^44^ In recent years, considerable efforts have been made to activate Ir­(III)-based PSs using light within the phototherapeutic window. One strategy involves attaching upconversion nanoparticles (UCNPs) to achieve NIR-triggered ROS generation.^45^ However, the low efficiency of UCNPs remains a significant bottleneck, limiting their clinical applications.^46^ Additionally, using NIR two-photon (2P) absorption often proves inefficient, as it requires a 2P excitation laser source with a minimal irradiation volume, reducing its effectiveness for treating large areas.^47^ Other strategies focus on developing deep-red/NIR absorbing iridium­(III)-based PSs through ligand design via π-extension and donor–acceptor interactions, as well as functionalization with organic fluorophores (e.g., BODIPYs, rhodamines, porphyrins, phthalocyanines and coumarins).^48–54^

COUPY fluorophores based on coumarin derivatives incorporating a cyano­(1-alkyl-4-pyridin-1-ium)­methylene group at the 2-position offer great potential in bioimaging applications (Figure 1A).^55^ Their photophysical properties can be easily tuned with minimal structural modifications, making them ideal for fluorescently labeling biomolecules, including peptides and lipids.^56,57^ Additionally, COUPY fluorophores show significant potential as PDT agents, whether in their free form,^58^ nanoencapsulated,^59^ or conjugated to cyclometalated Ir­(III) and Ru­(II) complexes, as shown in Figure 1C and 1D, respectively.^60–63^ Very recently, we have described a family of potent PSs based on Ru­(II) polypyridyl complexes incorporating 2,2′-bipyridyl ligands derived from COUPY coumarins, termed COUBPYs (Figure 1B). Ru-COUBPY complexes (Figure 1E) exhibit exceptional *in vitro* phototoxicity against colon cancer cells (CT-26) upon irradiation with light within the phototherapeutic window, both under normoxic and hypoxic conditions. Additionally, the *in vivo* safety and efficacy of one of the lead compounds were confirmed, showing a potent antitumor effect in a murine subcutaneous tumor model when exposed to deep-red light irradiation.^64^

**Figure 1:**
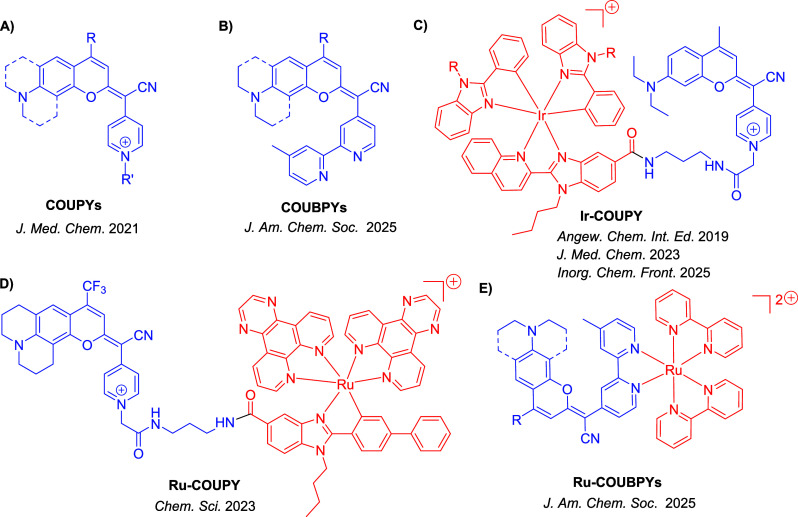
Structures of COUPY and COUBPY ligands, along with selected TMC-PSs containing them.

In this study, we describe for the first time the synthesis and biological evaluation of Ir-COUBPY complexes as novel PSs for efficient cancer phototherapy. Specifically, we developed five new cyclometalated Ir­(III) complexes, **Ir1a–Ir5a**, of the type [Ir­(C^N)_2_(COUBPY]^+^ (Figure 2), where C^N represents five deprotonated cyclometalated ligands, **HL1–HL5**, selected to establish structure–activity relationships (SAR). **HL5** was specifically chosen based on prior evidence demonstrating the chromophoric nature of 2-(5-arylthiophen-2-yl)­benzothiazoles in a series of dppz Ir­(III) PSs, which are capable of inducing oncosis in cancer cells.^43^ The new Ir-COUBPY complexes were assessed for their photophysical and photocatalytic properties, including their ability to photo-oxidate NADH,^65^ photogenerate ^1^O_2_ and ^•^OH, as well as their performance as PSs in 2D and 3D cancer models under red light irradiation. For comparative purposes, the photobiological properties of analogous Ir-bpy complexes **Ir1b–Ir5b** (Figure 2) were also studied. Additionally, we investigated the nanoencapsulation of a representative Ir-COUBPY complex using a polyurethane–polyurea hybrid nanocarrier based on ECOSTRATAR technology.^66–69^ Our findings underscore the potential of Ir-COUBPY complexes as dual inducers of PDT and ferroptosis, paving the way for their application in next-generation cancer treatments.

**Figure 2:**
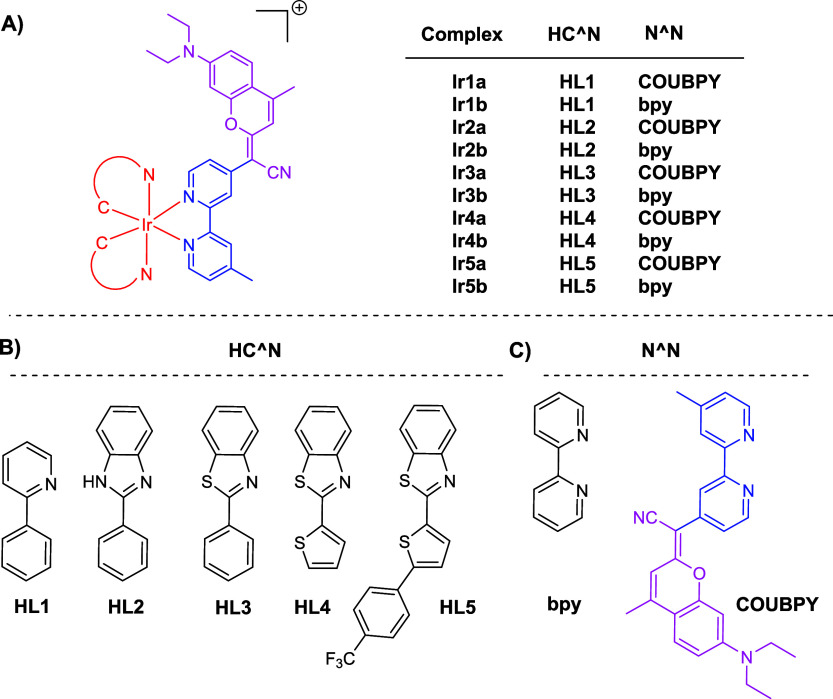
General structures of the iridium­(III) complexes **Ir1a–Ir5a** and **Ir1b–Ir5b** synthesized in this work (A) and of the ligands required to assemble them (B).

## Results and Discussion

### Design, Synthesis, and Chemical Characterization of Ir-COUBPY and Ir-bpy PSs

The required COUBPY ligand (Figure 2C), incorporating 2,2′-bipyridine at position 2 of the coumarin backbone, was synthesized as recently reported^64^ through a condensation reaction between 7-(*N,N*-diethylamino)-4-methyl-2-thiocoumarin^55^ and a 2,2′-bipyridyl acetonitrile precursor (Scheme S1). Conversely, the HC^N proligands **HL4** and **HL5** (Figure 2B) were prepared as illustrated in Scheme S2. **HL4** was synthesized via a condensation reaction between *o*-aminothiophenol and thiophen-2-carbaldehyde in high yield. **HL5** was prepared via Suzuki–Miyaura coupling starting from the bromoderivative **A**, as depicted in Scheme S2 in 60% yield.^43^ Three commercially available proligands—2-phenylpyridine, 2-phenylbenzimidazole, and 2-phenylbenzothiazole (**HL1**-**HL3**)—were also selected with the aim to stablish SAR.

The synthesis of **Ir1a**–**Ir5a** complexes as triflate salts was accomplished through a two-step process following reported standard literature procedures.^70,71^ Initially, the corresponding chloride-bridged dimeric iridium­(III) complexes, [Ir­(C^N)_2_(μ-Cl)]_2_, were reacted with the COUBPY ligand in a 1:2 molar ratio (Scheme S3). The resulting monomeric Ir­(III) complexes were fully characterized by ^1^H and ^13^C NMR spectroscopy (Figures S1–S10). The chloride salts were prepared for further characterization by ESI-MS and elemental analysis, as well as for biological studies. The positive ion ESI-MS spectra displayed the [M – Cl]^+^ peaks with the expected isotopic distribution pattern. The purity of the complexes was confirmed to be at least 95% through both elemental analysis of C, H, N, and S, as well as by reversed-phase HPLC-MS analysis, revealing a single peak in all cases (Figures S20–S25). The ^1^H NMR spectra of **Ir1a–Ir5a** displayed peaks around 1.1, 2.4, 2.55, and 3.45 ppm, corresponding to the aliphatic proton resonances of the COUBPY ligand. A set of peaks for each of the two nonequivalent C^N ligands from 6 to 9 ppm was also observed in the ^1^H NMR spectra due to the asymmetric nature of the coordination environment around the metal. The signal of the CF_3_ group of **Ir5a** was also detected by ^19^F NMR spectroscopy as a singlet at 61.16 ppm (Figure S11).

In addition, **Ir1b–Ir5b** complexes of the type [Ir­(C^N)_2_(bpy)]^+^ (Figure 2) were synthesized using similar procedures (see Experimental Section) to compare their photophysical properties and photobiological performance with those of the new [Ir­(C^N)_2_(COUBPY)]^+^ PSs (see Figures S12–S19 and S26–S27 for chemical characterization). Complexes **Ir1b** and **Ir3b** have been previously reported by other research groups,^4,5^ demonstrating notable electrogenerated chemiluminescence and sensing capabilities, respectively.

### Photophysical Characterization of Ir-COUBPY and Ir-bpy PSs

The photophysical properties of all Ir­(III) complexes were investigated in acetonitrile and water (1% DMSO). Their UV/vis absorption spectra are shown in Figures 3 and S28. All complexes exhibit intense absorption bands below 350 nm, attributed to spin-allowed π–π* transitions centered on the C^N and N^N ligands. The less intense and lower energy absorption bands (λ>350 nm) could be assigned to ligand-centered or charge-transfer bands (LLCT), and/or to spin-allowed (^1^MLCT) or spin-prohibited (^3^MLCT) metal–ligand charge-transfer bands. The latter result from the spin–orbit coupling of the heavy Ir­(III) atom (ζ=3909 cm^–1^).^43^ The UV–vis absorption spectra of the complexes **Ir1a–Ir5a** reveal an intense absorption band centered at approximately 540 nm, with molar extinction coefficients (ε) nearing 40,000 M^–1^ ·cm^–1^ in ACN. Notably, complex **Ir5a** presents the highest ε value, whereas **Ir1a** exhibits the lowest. As illustrated in Figure 3 and Table S2, Ir-bpy complexes containing C^N ligands with a higher degree of π-conjugation (**Ir2b–Ir5b** vs **Ir1b**) show larger molar extinction coefficient values, particularly within the 300–500 nm region. Moreover, the light absorption capability of **Ir4b** and **Ir5b** exceeds that of **Ir1b–3b**, which is attributable to the replacement of the phenyl ring in ligands **HL1**–**HL3** with the electron-rich thiophene core.

**Figure 3:**
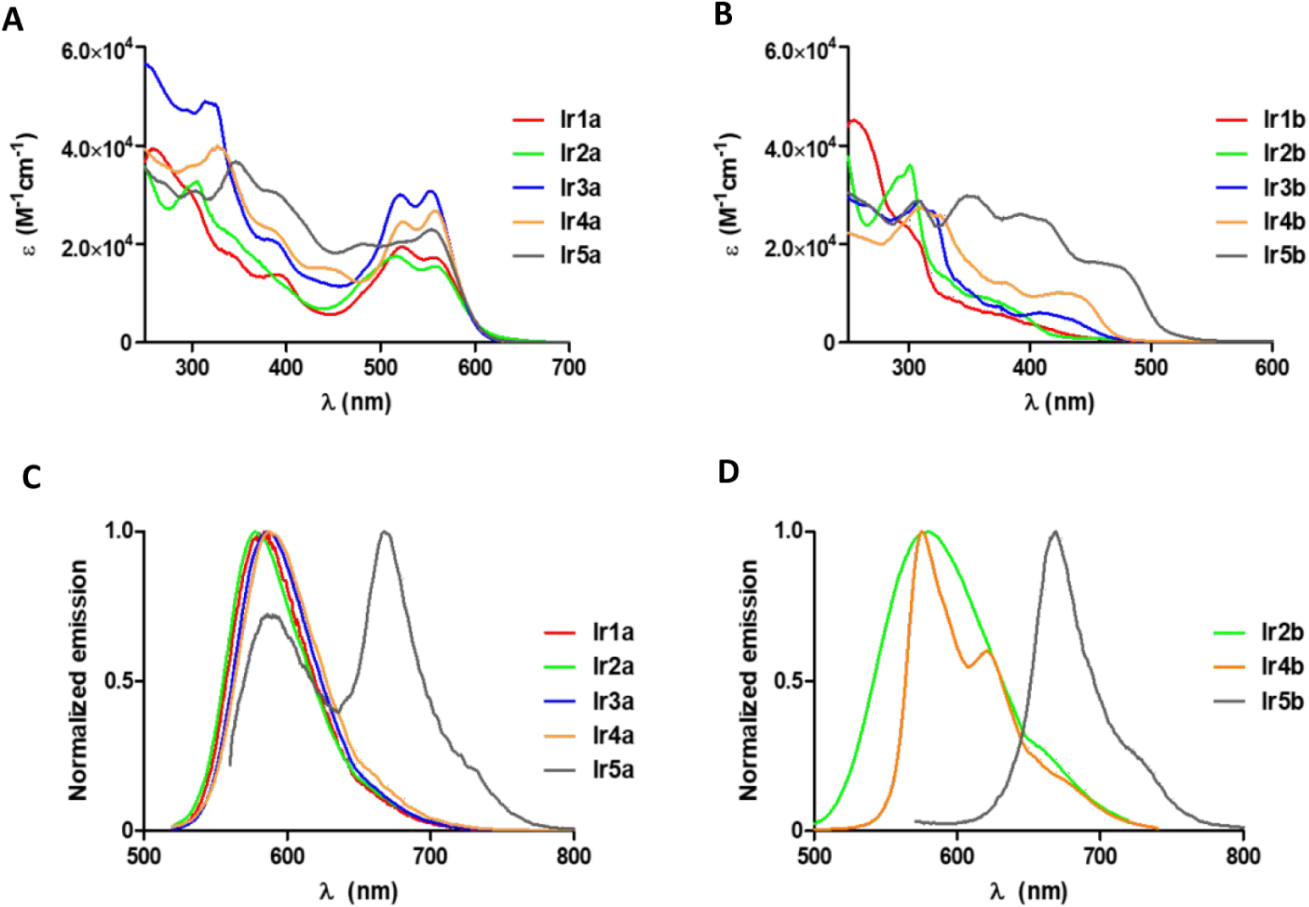
UV/vis spectra of Ir-COUBPY (A) and Ir-bpy complexes (B) in water (1% DMSO) at room temperature and normalized emission spectra of Ir-COUBPY (C) and Ir-bpy complexes (D) in acetonitrile at room temperature (bottom).

Notably, all Ir-COUBPY complexes exhibit two sharp, almost merged bands appearing in the 500–600 nm region that are not present in their bpy-containing counterparts. As shown in Figure S29, a comparative analysis of the absorption spectra of proligand **HL5**, **COUBPY** ligand, and **Ir5a** reveals that the absorption bands around 505 and 540 nm in the Ir-COUBPY complex correspond to metal–ligand charge transfer bands, attributable to the coumarin ligand. Similarly, all compounds containing the **COUBPY** ligand exhibit similar bands with molar extinction coefficient values ranging between 35,000 and 40,000 M^–1^ cm^–1^.

As shown in Figure 3, all Ir-COUBPY complexes exhibit an emission band in the far-red to NIR region with a maximum located around 585 nm in acetonitrile, regardless of the C^N ligand. However, the lifetimes vary significantly, from 266 ns (**Ir5a**) to 2.36 μs (**Ir3a**). **Ir5a** also exhibits an additional emission band at 668 nm, which closely aligns with the emission observed in the bpy-containing complex (**Ir5b**). Both complexes share similar characteristics in terms of emission maximum wavelength (∼669 nm), emission lifetime (∼1 μs), and emission quantum yield (<0.01), attributable to the presence of the C^N ligand. To further investigate the nature of the emission bands of the complexes with the **COUBPY** ligand, their emission spectra were also recorded in water (1% DMSO) at a concentration of 10 μM. As illustrated in Figure S30 and Table S3, the use of a more polar solvent induces a bathochromic shift of the emission band by up to 100 nm in the case of **Ir2a**. These findings suggest that the emission band exhibits a ^3^MLLCT character in the lower triplet state.^61^

In summary, the structural modifications implemented in the iridium complexes via the C^N ligands had a moderate influence on their photophysical properties. However, the incorporation of the coumarin fragment into the N^N ligand markedly altered the absorption characteristics of the Ir­(III) complexes, extending the absorption of the resulting Ir-COUBPY complexes into the green and red regions of the electromagnetic spectrum. These findings are consistent with previous results observed in Ru-COUBPY complexes^64^ and underscore the pivotal role of COUBPY ligands in designing PSs that operate within the phototherapeutic window.^54^

### Dark and Light Stability of Ir-COUBPY Complexes in DMSO and Biological Media

The stability of Ir-COUBPY complexes under dark conditions was evaluated in DMSO and RPMI culture medium (5% DMSO) using ^1^H NMR and UV/vis spectroscopy, respectively. No significant spectral changes were observed before and after 48 h of incubation (Figures S31–S42). To further assess their dark stability under biologically relevant conditions, complexes **Ir1a–5a** as well as their bpy-containing counterparts (**Ir1b–5b**) were incubated in RPMI culture medium supplemented with 10% fetal bovine serum (FBS), and 10% DMSO at 37 °C for 4 h. Reversed-phase HPLC analysis revealed minimal degradation of all complexes over time (Figures S43–S46), confirming their chemical stability and suitability for *in vitro* applications. Moreover, all Ir-COUBPY complexes exhibited high photostability when irradiated with green (λ= 520 nm, 1.8 mW/cm^2^) or blue (λ= 465 nm, 4.0 mW/cm^2^) light for 2 h (Figures S47–S48). Both dark and light stability are essential parameters to consider prior to evaluating the biological activity of a new PS.

### Photooxidation of NADH and Evaluation for ROS Photogeneration in Cell-Free Media

Subsequently, we investigated the impact of structural modifications on ROS photogeneration by Ir-COUBPY complexes. First, ROS generation was assessed through the photooxidation of NADH.^10^ NADH is crucial for maintaining intracellular redox balance and plays an essential role in the mitochondrial electron transport chain, where it is converted to NAD^+^. Disruptions in the NADH/NAD^+^ ratio can inhibit ATP synthesis, thereby depriving cancer cells of the energy required for proliferation. This ratio is therefore critical for cellular metabolism.^11,12^

To evaluate the ability of the compounds to induce photocatalytic oxidation of the coenzyme in aerated solutions, iridium complexes (5 μM) were incubated with NADH (100 μM) in PBS (5% DMF). As shown in Figures S49 and S50, the absorbance of NADH gradually decreased in the presence of all Ir-COUBPY complexes after irradiation with both green (520 nm) and red (620 nm) light. In contrast, among the bpy-containing analogues, only the **Ir5b** complex exhibited a slight ability to oxidize NADH, while the others remained unchanged (Figure S51). The most notable differences were observed when the C^N ligand was 2-phenylpyridine, as the turnover frequency (TOF) value, which measures the efficiency of the catalytic process, increased significantly from 0 to 292 h^–1^ upon the incorporation of the coumarin fragment into the N^N ligand (Table S4). This indicates that the structural modification of adding the coumarin fragment substantially enhances the photocatalytic activity of the iridium complexes.

Spectroscopic studies indicate that NADH photooxidation proceeds via single-electron transfer from NADH to the photosensitizer in its triplet state, producing reduced photosensitizer radicals that are rapidly reoxidized by molecular oxygen to generate ^•^O_2_^–^.^72^ UV–vis assays using nitroblue tetrazolium, which yields the blue formazan dye with λ_abs_ = 590 nm upon reduction, showed a marked increase in absorbance at 590 nm when **Ir1a–Ir5a** were irradiated in the presence of NADH (Figure S52) strongly supporting the involvement of superoxide and a Type I triplet-state mediated mechanism. Compound **Ir2a** is the one that leads to the highest generation of ^•^O_2_^–^.

Next, we further investigated the ability of Ir-COUBPY complexes to photogenerate type I and type II ROS in a cell-free medium. Initially, the singlet oxygen quantum yields of **Ir1a–Ir5a** complexes were determined in acetonitrile after green light irradiation (520 nm, 0.5 mW/cm^2^), using 1,3-diphenylisobenzofuran (DPBF) as a chemical trap. DPBF decomposes into a colorless product upon reaction with singlet oxygen.^13,14^ The UV/vis spectra of DPBF (50 μM) were recorded in the presence of Ir-COUBPY complexes (1–5 μM) at various time intervals postirradiation (Figure S53). As illustrated in Figure 4A and detailed in Table S4, **Ir4a** and **Ir5a** exhibit moderately higher singlet oxygen quantum yields (Φ_Δ_ = 0.46 and 0.79, respectively) compared to the other Ir-COUBPY complexes, which can be attributed to the incorporation of the thienyl fragment within their structures.

**Figure 4:**
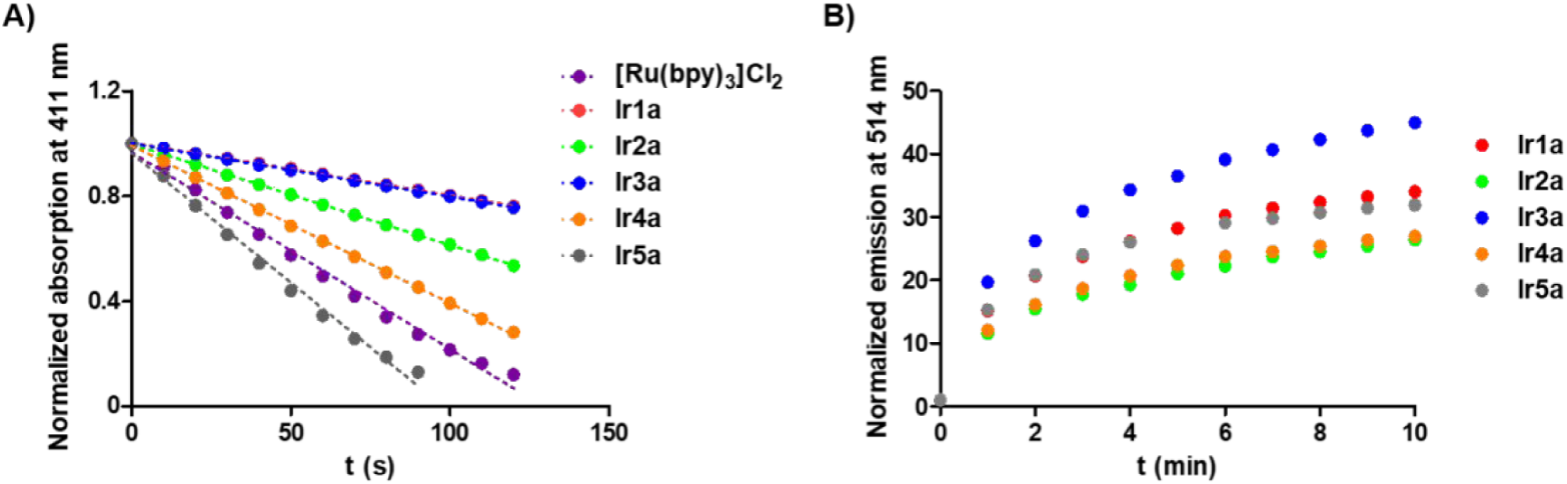
A) Normalized absorptivity of DPBF at 411 nm upon photoirradiation of **Ir1a–Ir5a** complexes at 520 nm (0.5 mW/cm^2^) in acetonitrile. [Ru­(bpy)_3_]­Cl_2_, with a reported singlet oxygen quantum yield of 0.57 (λ_ex_ = 520 nm) was used as a reference.^43^ B) Normalized emission spectra of HPF at 514 nm upon photoirradiation of **Ir1a–Ir5a** complexes at 520 nm (2.0 mW/cm^2^) in PBS (5% DMF).

**Table 1:** (Photo)­cytotoxicity of Ir-COUBPY Complexes Towards A375 Cancer Cells Expressed as IC_50_ Values (μM)^a^

	IC_50_ Values (μM)^a^			PI^b^	Normalized IC_50_ Values (μM)^a^,^c^		
Compound	Dark	Green	Red	Green	Dark	Green	Red
Ir1a	0.103 ± 0.006	0.009 ± 0.003	0.028 ± 0.003	11.4	0.103	0.009	0.028
Ir2a	>100	0.5 ± 0.2	7.7 ± 08	>200	>13	0.065	0.91
Ir3a	3.3 ± 1.0	0.097 ± 0.017	0.588 ± 0.049	34.0	2.15	0.063	0.382
Ir4a	4.8 ± 0.5	0.075 ± 0.007	0.123 ± 0.007	64.0	1.49	0.023	0.038
Ir5a	>100	9.3 ± 0.9	10.9 ± 2.4	>10.8	>5.4	0.502	0.589
NC-Ir4a	17.6 ± 4.7	0.098 ± 0.025	0.290 ± 0.031	179.6	7.4	0.041	0.122

a Experimental conditions: cells were incubated for 1 h at 37 °C, followed by either 1 h in the dark or irradiation under green (520 nm, 1.5 mW/cm^2^) or red (620 nm, 15 mW/cm^2^) light. Cell viability was determined after 48 h using the MTT assay.

b Phototherapeutic index (PI) = IC_50_(dark)/IC_50_(light).

c IC_50_ values normalized by Ir accumulation of **Ir1a** according to ICP-MS analysis (Figure 7).

Once it was demonstrated that **Ir1a–Ir5a** complexes generate singlet oxygen, we investigated their capacity to produce hydroxyl radicals using a spectroscopic method based on the oxidation of the nonfluorescent hydroxyphenyl fluorescein (HPF).^1,15,16^ Upon green light irradiation, all complexes (10 μM) were able to oxidize HPF (10 μM), resulting in bright green fluorescence. As shown in Figures 4B and S54, the incorporation of the benzothiazole fragment, in contrast to pyridine and benzimidazole derivatives, significantly enhances ^•^OH production under green light irradiation. Conversely, replacing the phenyl fragment with a thienyl ring or its derivatives leads to a marked reduction in hydroxyl radical production under the same conditions.

To gain deeper insight into the ROS generation, electron paramagnetic resonance (EPR) spectroscopy was employed, providing direct evidence of both Type I and Type II light-induced ROS formation. Complex **Ir4a** was selected as a representative example for these studies. The spin trap TEMP-NH_2_ was used to detect ^1^O_2_ under green-light irradiation (520 nm), while DMPO was employed to trap the photogenerated ^•^O_2_^–^ under orange-light irradiation (580 nm), both in methanol. As shown in Figure S55, irradiation of **Ir4a** at 520 nm produced the characteristic EPR triplet signal (1:1:1 ratio) associated with the TEMPO-^1^O_2_ adduct, confirming Type II ROS generation. Under orange-light irradiation, a four-line EPR signal (1:1:1:1 ratio) corresponding to the DMPO-•O_2_^–^ adduct was observed, demonstrating efficient Type I photogeneration of superoxide (Figure S56). Importantly, no paramagnetic signals were detected in the dark, indicating that both Type I and Type II ROS formation are exclusively phototriggered processes. Furthermore, the superoxide quantum yield of **Ir4a** (Φ^•^O_2_^–^ = 0.048) was determined using Rose Bengal (RB) (Φ^•^O_2_^–^ = 0.20) as a reference,^73^ following a standard comparative method described in the SI (Figure S57). Solutions of **Ir4a** and RB were adjusted to comparable absorbance at 580 nm and irradiated in the presence of DMPO. EPR spectra were recorded at different time points, and the second integral of each spectrum was plotted as a function of irradiation time. The slope of the linear fit was used to calculate Φ^•^O_2_^–^, confirming significant superoxide photogeneration by **Ir4a** under light irradiation.

Overall, these results confirm the capacity of Ir-COUBPY complexes to photogenerate both type I and type II ROS, mirroring the behavior of Ru-COUBPY complexes.^64^

### Cellular Uptake Studies by Confocal Microscopy and ICP-MS

Given that cellular uptake and subcellular accumulation are critical for a drug’s pharmacological effects, we next investigated the intracellular accumulation of the Ir-COUBPY complexes in cancer cells, comparing them with Ir-bpy complexes lacking the coumarin moiety.

We initially examined the cellular uptake of the compounds in living cervix adenocarcinoma (HeLa) cells using confocal laser imaging microscopy (CLSM), leveraging the intrinsic complexes luminescent properties of the iridium complexes for visualization. Upon excitation with violet light (405 nm), all Ir-bpy complexes exhibited a clear luminescent signal after just 30 min of incubation (Figure 5, top panels). A concentration of 5 μM was sufficient for **Ir1b**, **Ir3b**, and **Ir4b**, while a higher concentration of 20 μM was required to visualize **Ir2b** and **Ir5b** (Figure S58). Similarly, Ir-COUBPY complexes **Ir1a**, **Ir3a**, and **Ir4a** were easily visualized after 30 min of incubation at a concentration of 5 μM (Figure 5, bottom panels), following irradiation with green or yellow light (514 nm for **Ir4a** and 561 nm for **Ir1a** and **Ir3a**). This highlights the role of the coumarin fragment in enabling visualization with longer wavelength light excitation. Unfortunately, complexes **Ir2a** and **Ir5a** remained undetectable at all tested excitation wavelengths (405, 488, 514, and 561 nm), even after 2 h of incubation at a concentration of 20 μM, likely due to their poor cell permeability. Notably, all detected compounds exhibited a luminescent signal with a distinctive filamentous staining pattern, suggesting mitochondrial accumulation.

**Figure 5:**
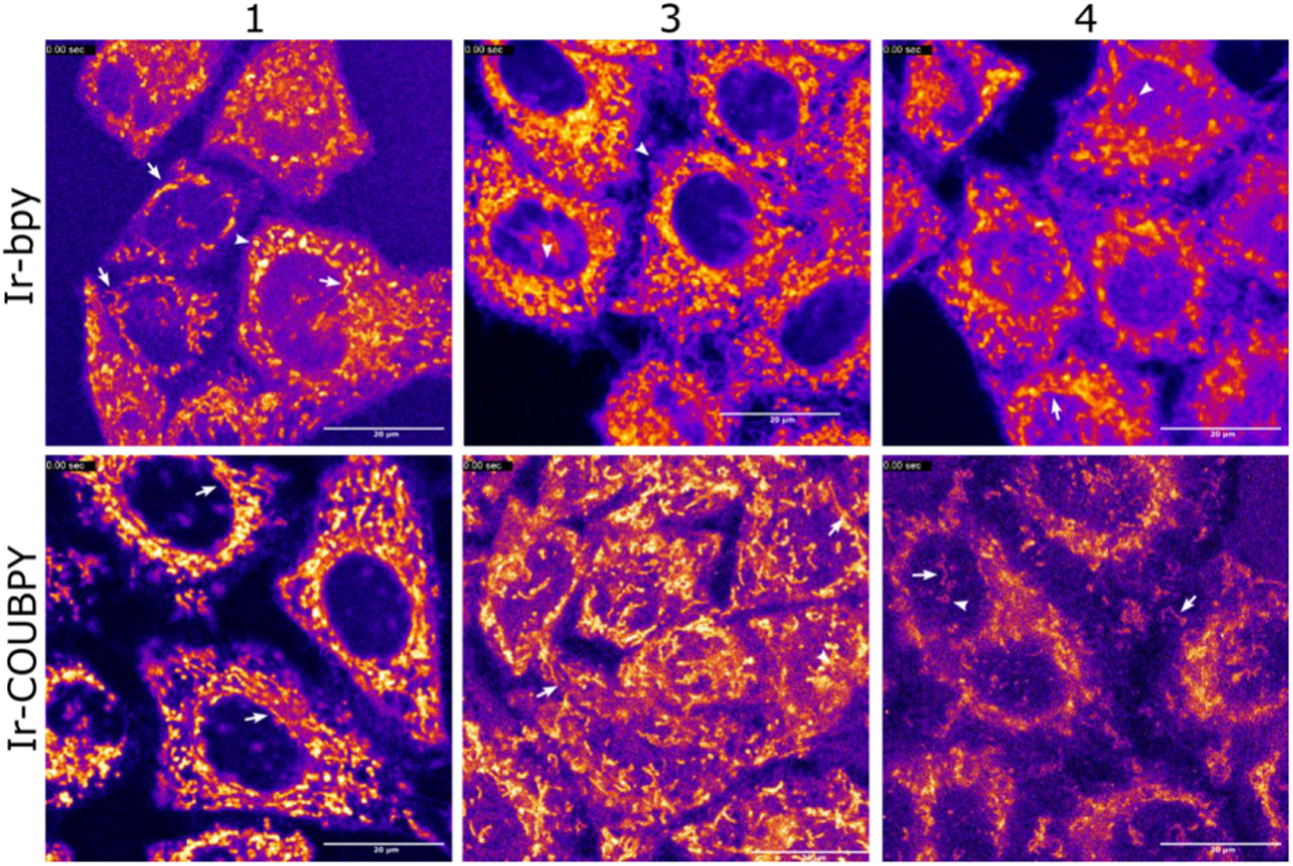
Cellular uptake of Ir-bpy complexes **Ir1b**, **Ir3b**, and **Ir4b** (top panels) and Ir-COUBPY complexes **Ir1a**, **Ir3a**, and **Ir4a** (bottom panels) in living HeLa cells, visualized by confocal light scanning microscopy. Images show single confocal planes of HeLa cells incubated with the compounds (5 μM) for 30 min at 37 °C. Ir-bpy complexes were excited at 405 nm, with emission detected between 500–625 nm. For Ir-COUBPY complexes, **Ir1a** and **Ir3a** were excited at 561 nm with emission detected between 570–640 nm, while complex **Ir4a** was excited at 514 nm with emission detected between 520–640 nm. Fluorescence images use the Fire LUT. White arrows indicate mitochondria, and white arrowheads highlight doughnut-shaped mitochondria. Scale bar: 20 μm.

To verify the subcellular localization of the compounds, a series of colocalization experiments using the mitochondria-specific fluorescent marker MitoView 650 were conducted. As shown in Figures 6 and S59, the luminescent signal of the Ir-bpy and Ir-COUBPY complexes strongly overlapped with that of MitoView 650. Additional evidence supporting the colocalization of the Ir complexes with MitoView 650 was provided by the high values obtained for both Pearson’s correlation coefficient (PCC) and Mander’s colocalization coefficient (M1, indicating the colocalization of the complexes within the MitoView 650 channel, and M2, indicating the colocalization of MitoView 650 within the complexes’ channel) (Tables S5 and S6). Collectively, these findings confirm the preferential accumulation of the complexes in the mitochondria.

**Figure 6:**
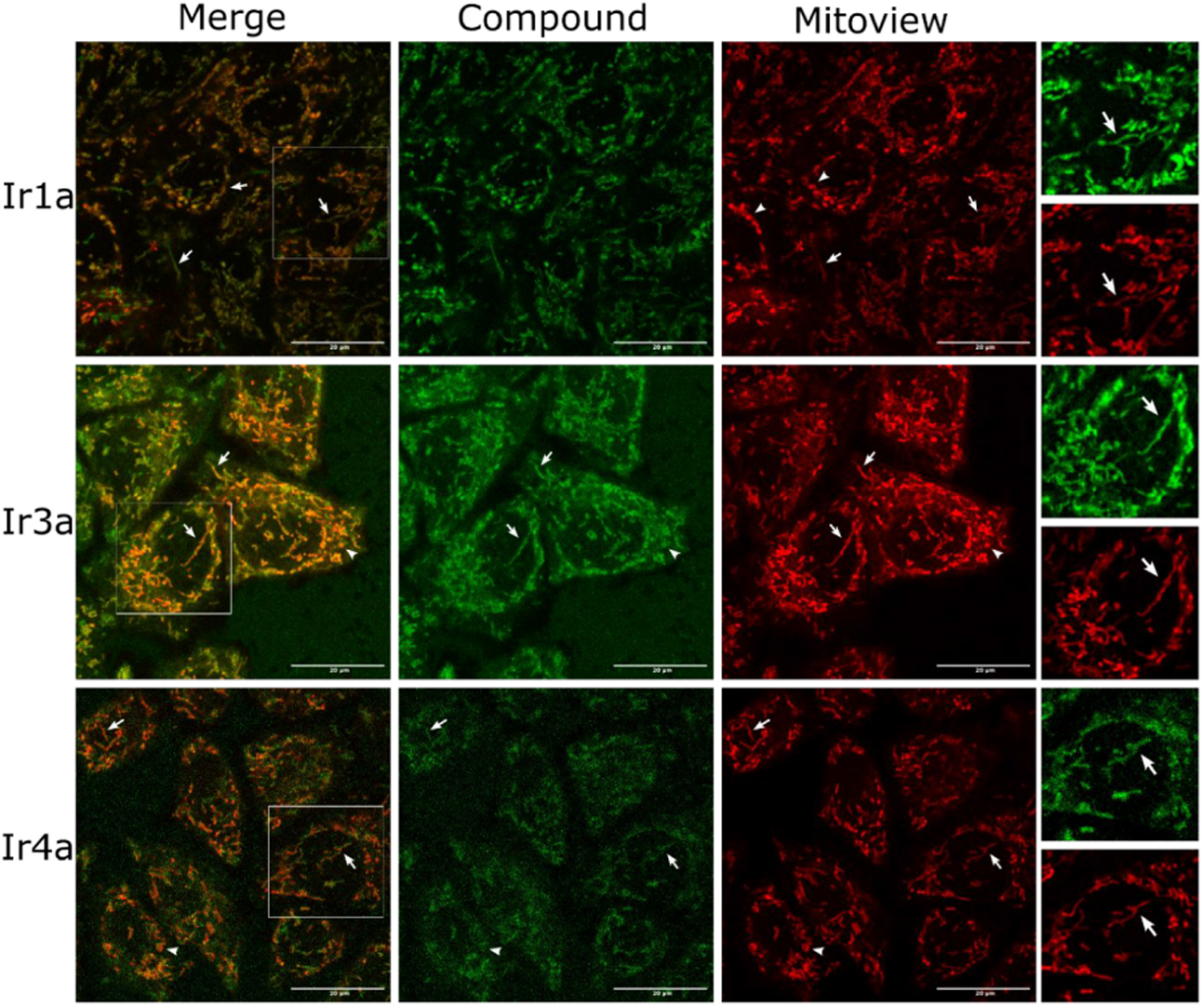
Co-localization of Ir-COUBPY complexes **Ir1a**, **Ir3a**, and **Ir4a** with MitoView 650 in HeLa cells. Images show single confocal planes of cells incubated with the compounds (5 or 20 μM, green) and MitoView 650 (0.1 μM, red). Panels: Left, overlay of both signals; center-left, Ir complex fluorescence; center-right, MitoView 650 fluorescence; right, insets of white squares in left panels. Inset images were processed to remove the background and enhance the intensity of both channels. White arrows and arrowheads indicate colocalization of both signals in mitochondria and in doughnut-shaped mitochondria, respectively. Scale bar: 20 μm.

To gain further insights into the cellular uptake of the compounds, the intracellular iridium content was quantified by ICP-MS in malignant melanoma A375 cells, which was one of the cell models used in *in vitro* (photo)­cytotoxicity studies (*vide infra*). Notably, some of the Ir-COUBPY complexes showed higher intracellular accumulation compared to their Ir-bpy counterparts after incubation of the cells with the compounds for 1 h at a concentration of 10 μM (Figure 7), which indicates that the coumarin fragment can also improve the cellular uptake of the complexes. Furthermore, a significant difference in intracellular accumulation was observed depending on the C^N ligand, with complexes containing 2-phenylpyridine and 2-phenylbenzothiazole ligands showing the highest accumulation efficiency. In summary, the intracellular accumulation of Ir-COUBPY complexes followed the order: **Ir1a** > **Ir3a** > **Ir4a** ≫ **Ir2a** ≈ **Ir5a**, with a comparable trend also observed for the Ir-bpy counterparts. These results are consistent with those from confocal microscopy studies that revealed that Ir-COUBPY complexes **Ir2a** and **Ir5a** could not be observed despite using long incubation times and high concentrations.

**Figure 7:**
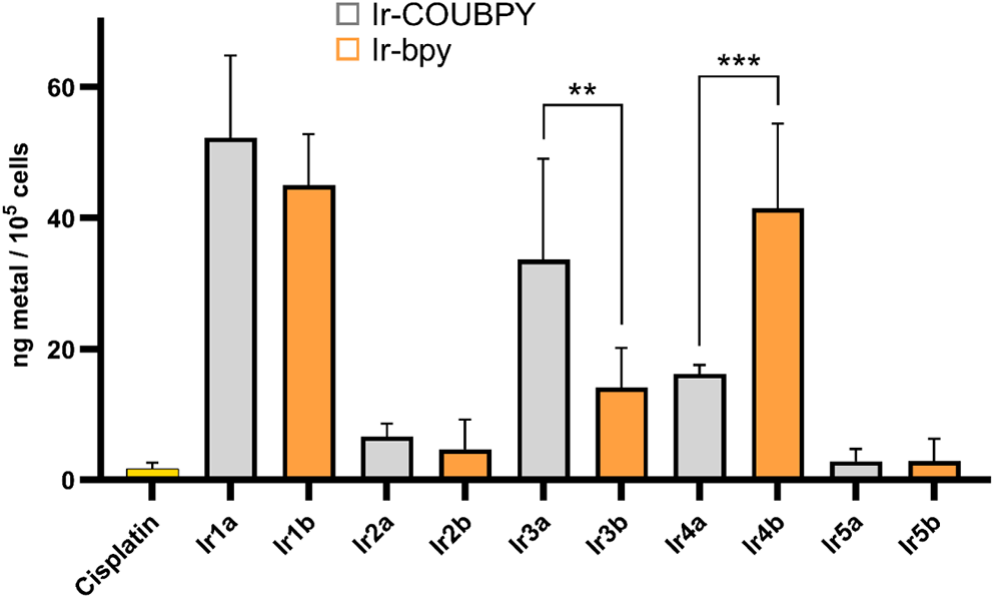
Intracellular accumulation of iridium content was quantified by ICP-MS in A375 cells treated with **Ir1a–Ir5a** and **Ir1b–Ir5b** complexes (10 μM) for 1 h. The uptake of Pt (yellow bar) is set as the positive control. Data for intracellular Ir concentration presented as the mean ± SD from two independent tests. Statistical significance was determined using a two-tailed Student’s test (*p < 0.05, **p ≤ 0.01 or ***p ≤ 0.001).

Since lipophilicity plays a crucial role in the ability of drugs to cross biological membranes, the partition coefficients (log *P*) of the Ir­(III) complexes were measured to determine whether differences in cellular uptake could be attributed to variations in lipophilicity. As shown in Table S7, the Ir-bpy series (**1b–5b**) displayed notable differences in log *P* values, with complex **1b** being the most hydrophilic and complexes **2b** and **5b** the most hydrophobic (Lipophilicity: **Ir1b** ≪ **Ir3b** ≈ **Ir4b** ≪ **Ir2b** ≪ **Ir5b**). This trend accounts for the lower intracellular accumulation of the most hydrophobic complexes, possibly due to reduced water solubility. In the Ir-COUBPY series (**1a–5a**), a general increase in lipophilicity was observed compared to their bpy counterparts. Although the relative order of lipophilicity was maintained, the differences were more subtle, suggesting that the COUBPY ligand exerts a stronger influence over lipophilicity than the C^N ligands.

### Nanoencapsulation of Ir4a in Polyurethane–Polyurea Hybrid Nanocapsules

Based on cellular uptake studies conducted using confocal microscopy and ICP-MS, we examined the nanoencapsulation of a representative Ir-COUBPY complex (**Ir4a**) within polyurethane–polyurea hybrid nanocapsules. Our aim was to evaluate the impact on the cellular uptake of the PS and its photobiological properties. This type of nanoparticle has previously been described as an effective carrier for delivering hydrophobic compounds,^68,69^ including coumarin-based PSs^59^ and Ir-based anticancer agents, enhancing their stability, bioavailability and biological performance.^49,74^

The nanoencapsulation of **Ir4a** involved two main processes, as depicted in Figure S61: the synthesis of an amphiphilic polyurethane–polyurea amino terminal-reactive prepolymer (steps I–III) and the Ir­(III) complex nanoencapsulation (steps IV–VII). First, the polymerization between diol and the diisocyanate monomers afforded an NCO-terminal prepolymer (step I, Table S8). Upon reaching the theoretical percentage of reacted isocyanate groups, the prepolymer was dissolved in THF and added dropwise to a slight excess of a hydrophobic diamine solution, also dissolved in THF (step II). The reaction was completed by addition of primary amines (step III).

For the nanoencapsulation process, the NH_2_-terminal polymer was reactivated with an excess of isophorone diisocyanate (IPDI) in the presence of **Ir4a** (step IV, Table S9). Once the presence of isocyanate groups was confirmed by FT-IR, a basic solution of l-Lysine was added dropwise under mechanical stirring, forming a water-in-oil nanoemulsion (step V). After 5 min, phase inversion was promoted by the addition of water (step VI), and upon addition of diethylenetriamine (DETA) (step VII), the cross-linked Ir-loaded nanocapsules (**NC-Ir4a**) were obtained. **NC-Ir4a** were purified by dialysis using a molecular porous membrane tubing with 12–14 kDa MWCO.

As illustrated in Figure 8A, the presence of PEGylated and ionomeric groups, positioned opposite to the core-oriented lipophilic tails, enhances aqueous solubility and stabilizes the metallodrug through hydrophobic interactions, promoting the stratification of the nanocapsule wall. The size of the Ir­(III)-loaded nanocapsules was studied using dynamic light scattering (DLS), which revealed an average hydrodynamic diameter of 11.69 ± 0.27 nm (Figure 8B and Table S10). These results are consistent with the particle size distribution observed by transmission electron microscopy (TEM), which confirmed a roughly spherical shape and homogeneous morphology (Figures 8C and S62).

**Figure 8:**
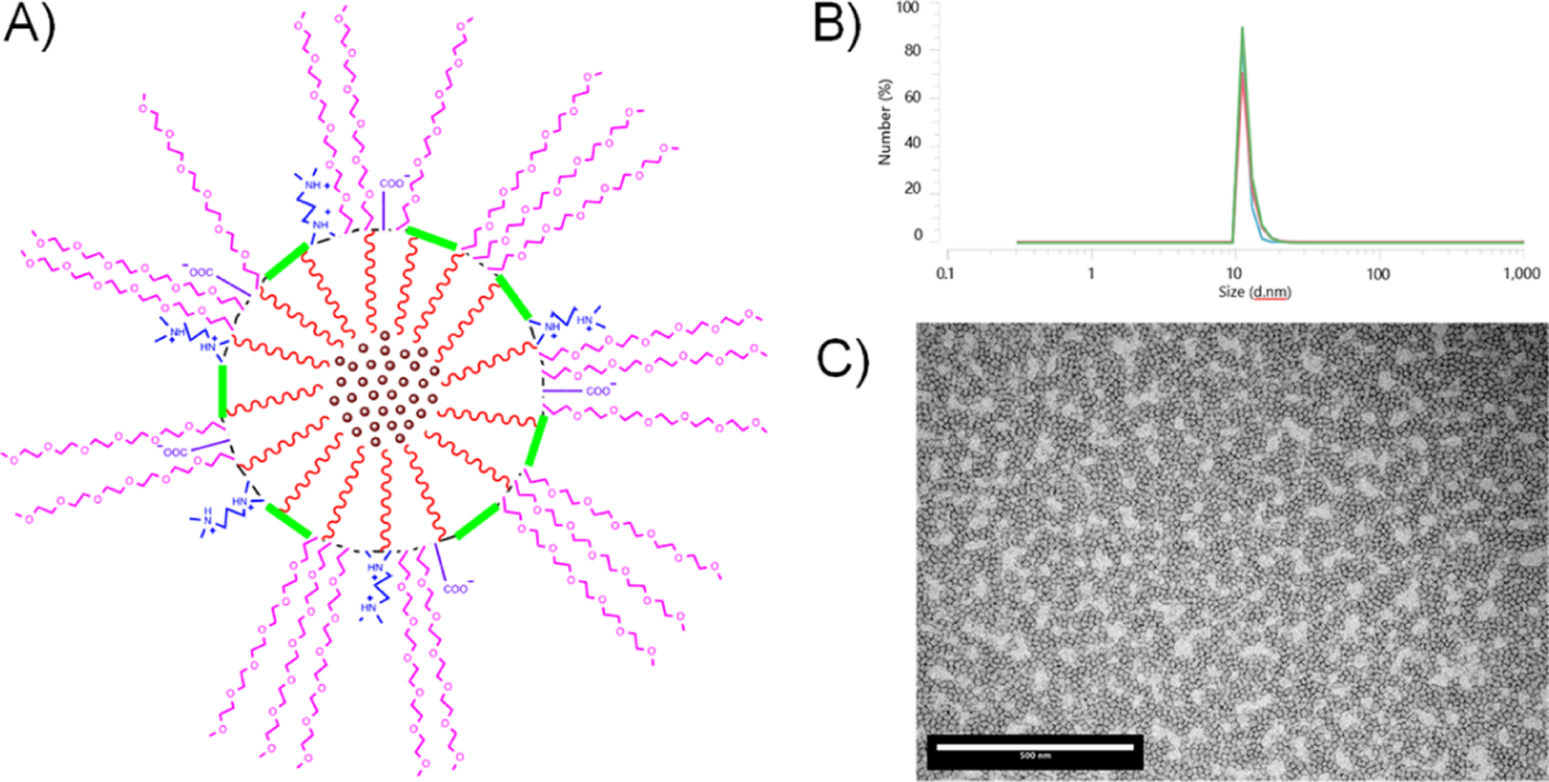
A) Schematic representation of the distribution of the hydrophilic and hydrophobic tails around the lipophilic core of an Ir-loaded nanocapsule. B) Hydrodynamic diameter average of **NC-Ir4a** measured by DLS. C) Particle size distribution measured by TEM micrography of **NC-Ir4a** (scale bar: 500 nm).

To further characterize the nanocarrier, the zeta potential of **NC-Ir4a** was measured at different pH values (6.0, 6.5, 7.0, and 7.5) to assess the amphoteric behavior of the polymeric shell. As shown in Table S11 and Figure S63, the nanocapsules exhibit a slightly less cationic surface charge at physiological pH (7.4) compared to more acidic conditions. This property is particularly relevant, as it contributes to prolonged circulation time in the bloodstream and facilitates gradual accumulation in abnormally vascularized tumor tissues. In such mildly acidic environments (pH 5.8–6.9), the increased cationic character enhances cellular internalization.

Additionally, the degradability of the Ir­(III)-loaded nanocapsules was evaluated by TEM in the presence of glutathione (GSH), simulating the intracellular reductive environment typical of cancer cells. As shown in Figure S64, clear morphological differences were observed between the untreated control and samples incubated in GSH-supplemented PBS for 24 and 48 h at 40 °C. After 24 h, initial signs of morphological disruption were evident, which became more pronounced after 48 h, confirming the degradability of the nanocapsules under reductive conditions.

As demonstrated in Figure S65, the nanoformulation enabled the detection of **Ir4a** inside the cells by confocal microscopy after 30 min of incubation at 20 μM, upon excitation with 514 nm light. Similar to observations with the free Ir-COUBPY complex **Ir4a**, a filamentous staining pattern indicative of mitochondrial accumulation was noted after incubating the cells with the nanoformulation. This suggests that the nanocapsules disaggregate readily inside cells postinternalization, facilitating the release of the Ir-COUBPY complex. Colocalization studies with MitoView 650 further confirmed the accumulation of **Ir4a** in mitochondria (Figure S66 and Table S12). Notably, nanoencapsulation significantly enhanced the intracellular accumulation of **Ir4a** compared to its nonformulated counterpart (Figure S67). This increased accumulation likely contributes to the improved phototoxic efficiency observed for **NC-Ir4a** (*vide infra*).

### Evaluation of Phototoxic Activity of Ir-COUBPY and Ir-bpy Complexes

The antiproliferative activity of Ir-bpy and Ir-COUBPY complexes was initially assessed against A375 and HeLa cancer cell lines, both in the dark and under green light irradiation (520 nm, 1.5 mW/cm^2^) (Tables 1 and S13–S15). The IC_50_ values obtained for both cell lines revealed distinct trends in cytotoxic activity under dark and light conditions, depending on the nature of the C^N and N^N ligands. Normalization of IC_50_ values in the A375 cell line, using data from ICP-MS cellular uptake studies, allowed for an accurate SAR analysis by accounting for differences in intracellular accumulation of the compounds.

Ir-COUBPY complexes **Ir1a–Ir4a** exhibited strong nanomolar cytotoxic activity after green light irradiation, regardless of the C^N ligand **HN1–4** (e.g., IC_50_ = 23 nM, PI = 64 for **Ir4a** in A375 cells; Table 1). In contrast, Ir complexes containing the **HL5** ligand showed submicromolar phototoxic activity in both series (IC_50_ = 0.50 μM for **Ir5a** and IC_50_ = 0.22 μM for **Ir5b** in A375 cells; Table S15). Similarly, **Ir2b–Ir4b** also demonstrated potent phototoxicity upon green light irradiation (e.g., IC_50_ = 74 nM, PI = 17 for **Ir4b** in A375 cells; Table S15). The reduced dark cytotoxicity of Ir-COUBPY complexes, compared to their bpy counterparts, resulted in significantly higher phototherapeutic indexes (PI) (e.g., PI = 64 for **Ir4a** vs PI = 5.4 for **Ir4b** in A375 cells), underscoring the crucial role of the coumarin ligand in enhancing the photodynamic efficiency of the compounds. Notably, as shown in Table S13, encapsulation of **Ir4a** into polyurethane–polyurea hybrid nanocapsules (**NC-Ir4a**) led to a remarkable improvement in PI values, increasing from 64 to 179.6 in A375 cells and from 22.1 to 154.5 in HeLa cells. This enhancement is primarily attributed to a decrease in dark cytotoxicity while maintaining relatively low IC_50_ values under green light irradiation, consistent with previous findings for COUPY-based PSs.^54^

To investigate the influence of light dose on photodynamic efficacy, we varied the irradiation time at a fixed irradiance (520 nm, 1.5 mW/cm^2^), applying exposure durations of 30, 60, and 90 min (Figure S68). A clear time-dependent response was observed, with differences depending on the formulation. For free **Ir4a**, photocytotoxicity at 30 and 60 min was comparable, with a further increase at 90 min. In contrast, **NC-Ir4a** showed a significantly enhanced effect at 60 min, which closely matched the response at 90 min and was markedly greater than at 30 min. These results suggest that the nanoformulation reaches near-maximal activity by 60 min under the tested conditions. This trend was consistent across the Ir-COUBPY series, where photocytotoxicity generally increased with irradiation time under otherwise identical conditions, while dark controls remained unaffected.

By leveraging the extended absorption of Ir-COUBPY complexes in the visible region, their PDT efficacy could be evaluated under red light irradiation (620 nm, 15 mW/cm^2^). As shown in Tables 1 and S13, all compounds retained high phototoxicity, with **Ir4a** and its nanoformulation being again the best performers.

### Intracellular ROS Photogeneration and Cell Death Study

After confirming the high photocytotoxicity of Ir-COUBPY complexes, we examined the ability of **Ir1a**, **Ir4a** and **Ir5a**, and **NC-Ir4a** to induce oxidative stress in A375 cells using the ROS probe 2,7-dichlorodihydrofluorescein diacetate (DCFH-DA) under green light irradiation. As shown in Figure S69, **NC-Ir4a** generated significantly higher ROS levels compared to **Ir4a**, reinforcing its enhanced photodynamic activity. Conversely, **Ir5a** produced much lower ROS levels relative to both **Ir4a** and **NC-Ir4a**, which correlates with its lower phototoxicity and underscores the critical role of ROS in effective cell death induction.

To identify the specific ROS species involved in the phototoxic process, we employed ROS scavengers. Figures S70A and S70B demonstrate that the addition of terephthalic acid (TPA), a hydroxyl radical scavenger, significantly reduced ROS levels, confirming the involvement of hydroxyl radicals in oxidative stress. This observation is consistent with cell-free experiments using the HPF probe. Sodium pyruvate (NaPyr), a hydrogen peroxide scavenger, moderately reduced ROS levels, suggesting that H_2_O_2_ plays a lesser role compared to hydroxyl radicals. Given the presence of ^•^OH and H_2_O_2_, which are key mediators of lipid peroxidation and ferroptosis, we investigated ferroptosis involvement using cell viability assays with the ferroptosis inhibitor ferrostatin-1 (Fer-1) and the pan-caspase inhibitor Z-VAD-FMK. As shown in Figure S70C, Fer-1 significantly rescued cell viability in **Ir4a**- and **NC-Ir4a**-treated cells, whereas Z-VAD-FMK had minimal effect, indicating that caspase-dependent apoptosis is not the primary mode of cell death Ir-COUBPY complex **Ir4a**. The stronger protective effect of Fer-1 against **NC-Ir4a** further supports the enhancement of ferroptotic cell death of **Ir4a** through nanoencapsulation. Furthermore, the absence of DNA damage suggests that apoptosis is not a major cell death pathway. Phosphorylation of H2AX (γH2AX), a marker of DNA double-strand breaks, was significantly increased in cisplatin-treated cells but remained comparable to control levels in **Ir4a**- and **NC-Ir4a**-treated cells (Figure S70D). The low γH2AX levels indicate that these compounds do not cause substantial genotoxic stress, which is a hallmark of apoptosis.^75^

### Redox Collapse and Photoinduced Lipid Peroxidation

Following the confirmation of hydroxyl radical involvement and ferroptosis in the phototoxic mechanism of **Ir4a** and **NC-Ir4a**, we further investigated redox disruption and lipid peroxidation (LPO) as downstream events of ROS generation. Upon green-light irradiation, **NC-Ir4a** triggered a pronounced oxidation of the BODIPY 581/591 C11 probe in A375 cells, as evidenced by CLSM imaging (Figure 9A). This was observed as an irradiation-dependent increase in the green fluorescence channel, corresponding to the oxidized form of the probe. Flow cytometry analysis (FL-1 channel, 488 nm excitation) confirmed this trend, showing elevated mean fluorescence intensity for oxidized BODIPY upon light exposure (Figure 9B).

**Figure 9:**
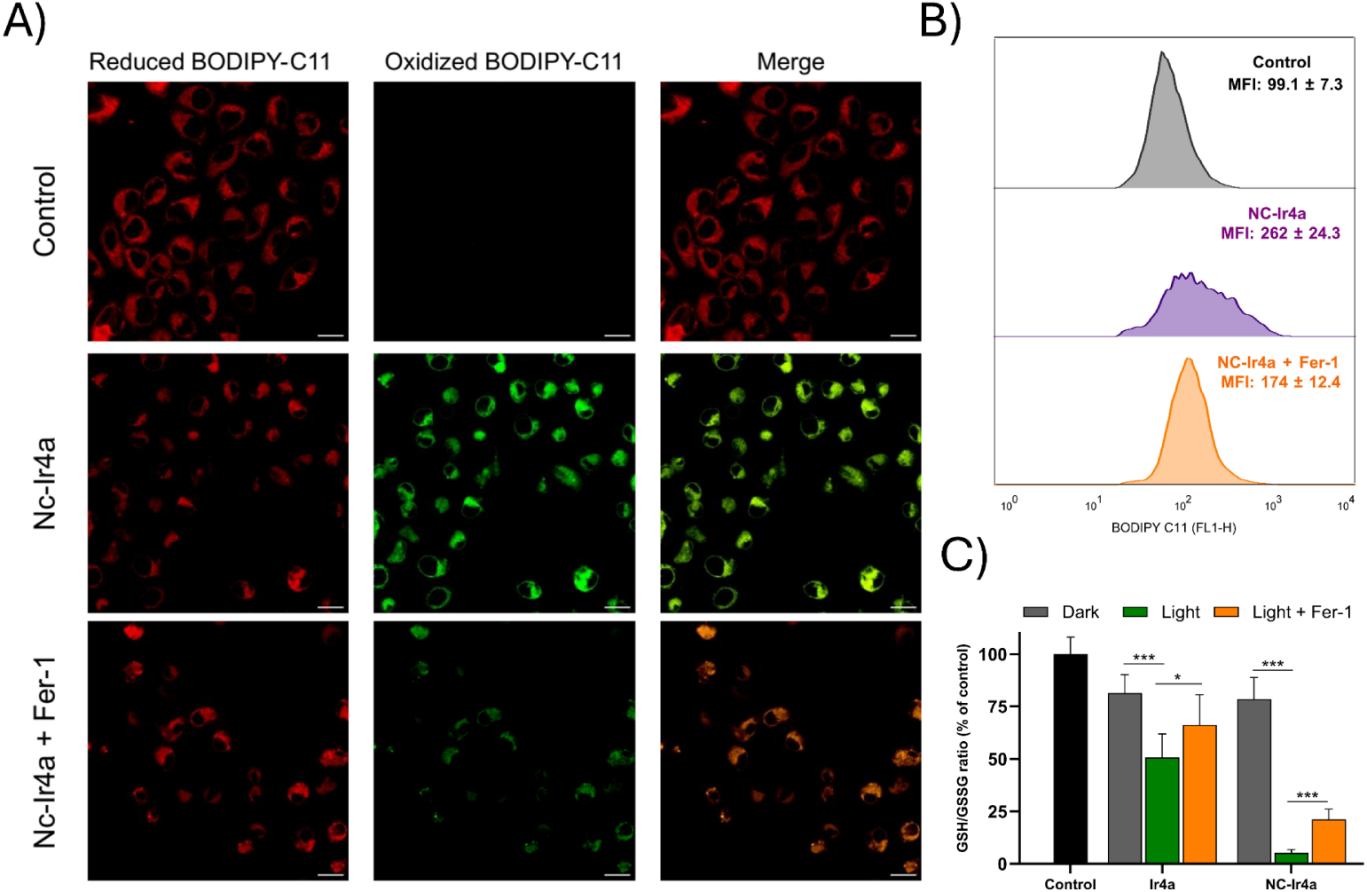
(A) Representative CLSM images of A375 cells stained with BODIPY 581/591 C11 following the indicated treatments under green-light irradiation. The green channel shows the oxidized probe (lipid peroxidation) and the red channel shows the reduced form. Scale bar: 20 μm. (B) Flow cytometric quantification of oxidized BODIPY C11 in A375 cells under light irradiation. The green channel reports the oxidized probe; values represent mean fluorescence intensity (MFI) for each treatment. (C) GSH/GSSG ratio measured at the same postirradiation time point, normalized to the irradiated untreated control. Bars represent mean ± SD (*n* = 3 independent experiments). Statistical significance was assessed by one-way ANOVA (*p < 0.05; **p < 0.01; ***p < 0.001).

In parallel assays performed at the same postirradiation time point, both **Ir4a-** and **NC-Ir4a**-treated cells exhibited a reduced GSH/GSSG ratio compared to the irradiated untreated control, and **NC-Ir4a** also caused a decrease in the absolute GSH levels, indicating a shift of the intracellular glutathione pool toward oxidation (Figures 9C and S71). This simultaneous increase in membrane LPO and glutathione oxidation indicates a collapse of the cellular redox buffering system, facilitating lipid peroxide accumulation. These results align with the ROS profiling and scavenger assays described above, and further support a hydroxyl-radical–biased Type I mechanism. Taken together, the redox imbalance, LPO, and inhibitor data reinforce ferroptosis—rather than caspase-dependent apoptosis—as the predominant cell death pathway under our irradiation conditions.

### Microscopy Imaging Analysis

Supporting the ferroptosis mechanism, widefield fluorescence microscopy images revealed significant ROS accumulation in A375 cells treated with **Ir4a** and **NC-Ir4a**, as indicated by dihydroethidium (DHE) staining, a ^•^O_2_^–^ probe.^8,9^ As shown in Figure S72, cells treated with **Ir4a** and **NC-Ir4a** exhibited similar fluorescence intensity, both much higher than in cells treated with light alone, where fluorescence intensity was negligible. Further evidence of ferroptosis induction by **Ir4a** and **NC-Ir4a** was obtained through field emission scanning electron microscopy (FE-SEM, Figure 10 and Figure S73). In contrast to apoptosis-induced cells treated with cisplatin, which showed distinct membrane blebbing—a characteristic feature of apoptosis- cells treated with **Ir4a** and **NC-Ir4a** displayed severe membrane damage without blebbing. This morphological change strongly correlates with the previously observed ROS generation and supports the induction of ferroptosis over apoptosis in cancer cells.^76^

**Figure 10:**
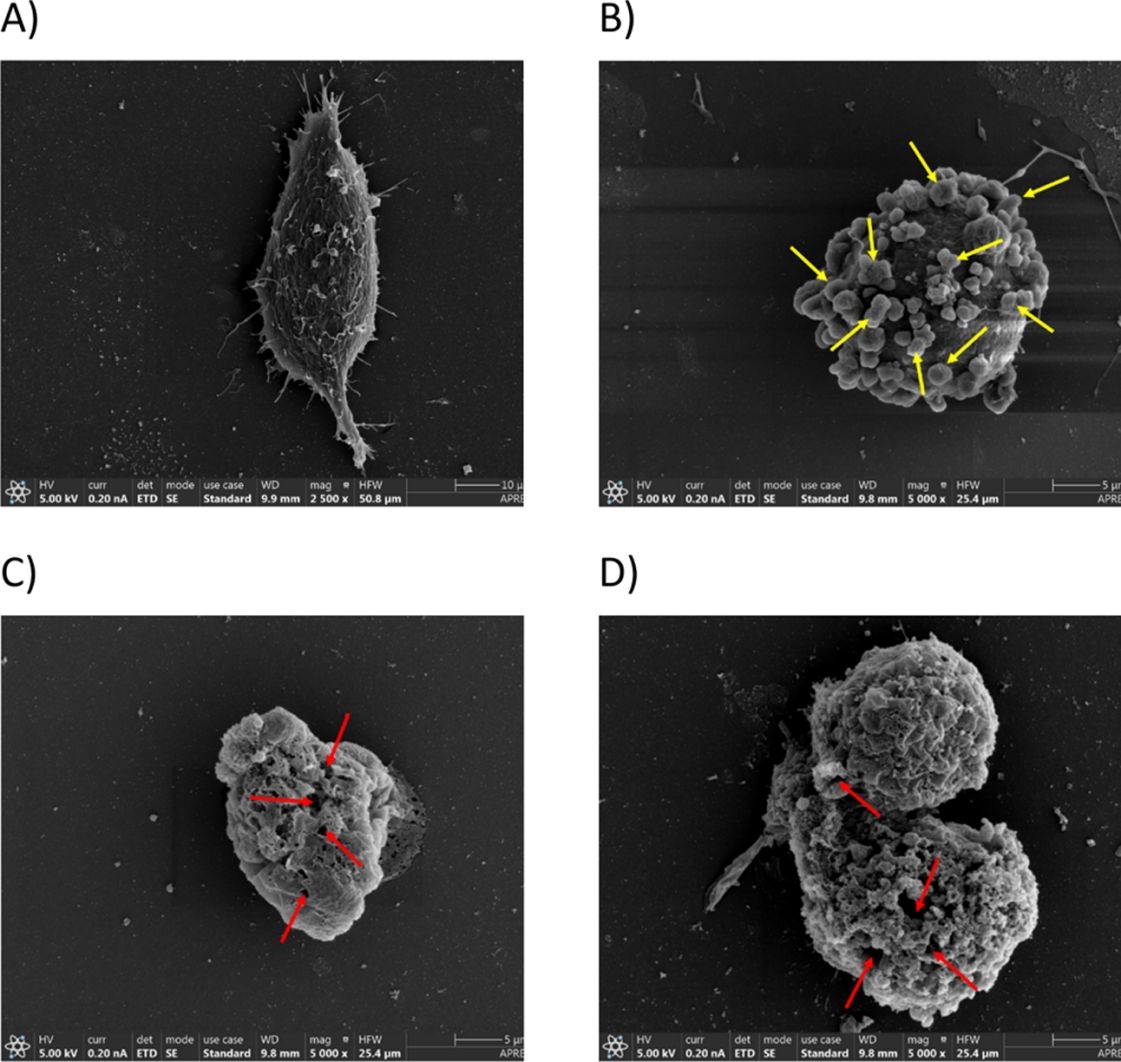
(A) Control, (B) cisplatin, (C) **Ir4a** and (D) **NC-Ir4a**-treated A375 cells showing morphological changes observed under FE-SEM. Yellow arrows indicate cell blebbing, while red arrows indicate membrane damage. Magnification: 2500× (control) and 5000× (treated cells).

### Mitochondrial Dysfunction, OCR Suppression and ATP Depletion

To further characterize the bioenergetic states and modes of cell death induced by **Ir4a** and **NC-Ir4a**, we employed the Seahorse XFe96 extracellular flux analyzer glycolytic rate assay. As shown in Figure 11A, photoactivation of **Ir4a** caused a notable suppression of oxygen consumption rate (OCR). This effect was even more pronounced in the photoactivated **NC-Ir4a**-treated cells compared to their dark counterparts, indicating significant mitochondrial respiratory inhibition. Importantly, both **Ir4a** and **NC-Ir4a**, in the absence of light, displayed OCR profiles similar to the untreated control, confirming that photodynamic activation is essential for mitochondrial disruption. Cisplatin, used as positive control for apoptosis, caused only moderate OCR reduction, distinguishing the mechanism of the photoactivated Ir-COUBPY compounds.

**Figure 11:**
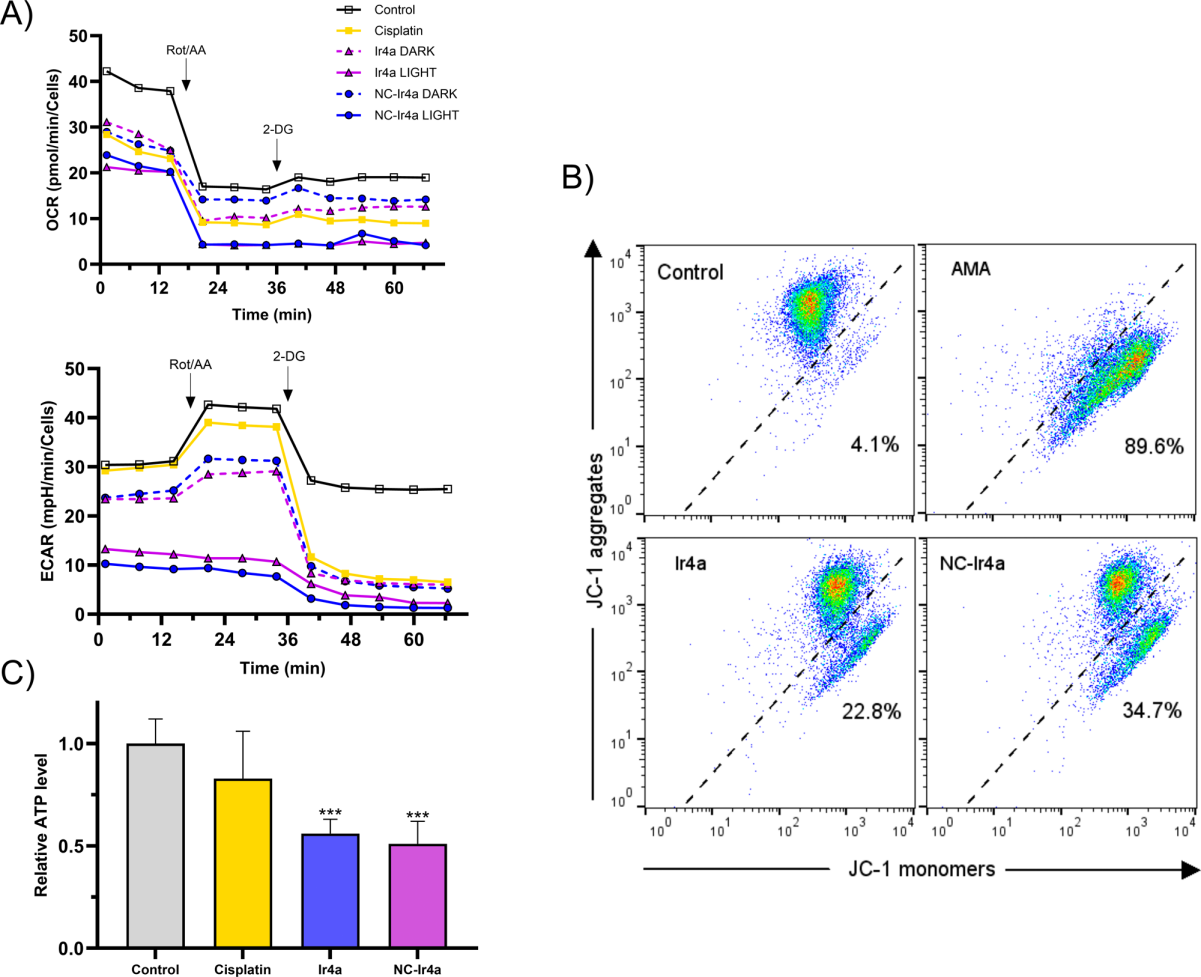
(A) Mitochondrial oxidative phosphorylation based on the OCR and ECAR after 2 h of treatment with cisplatin (10 μM, dark) and **Ir4a** and **NC-Ir4a** (dark and photoirradiated, 250 nm), measured using the Seahorse XFe analyzer. All data are represented as mean ± SD from three independent experiments. Statistical significance was determined via one-way ANOVA (*p < 0.05, **p < 0.01, **p < 0.001). (B) Impact of free and nanoencapsulated **Ir4a** on MMP, measured by JC-1 staining. Cells were treated with **Ir4a** and **NC-Ir4a** for 1 h, followed by 1 h of irradiation and a 6-h recovery period, stained with JC-1 for 30 min and analyzed by flow cytometry. λ_ex_ = 488 nm, λ_em_ = 525 ± 30 nm (JC-1 monomers) or 585 ± 30 nm (JC-1 aggregates). Antimycin A (AMA, 50 μM) was used as a positive control. (C) Relative ATP levels in A375 cells after 6 h of treatment with cisplatin (10 μM) and the indicated and **Ir4a** and **NC-Ir4a** (photoirradiated, 250 nm). Statistical significance (control vs treatment) was determined using Student’s *t* test (*p < 0.05, **p < 0.01, ***p < 0.001).

In parallel, extracellular acidification rate (ECAR) analysis revealed a decrease in glycolytic activity in light-treated cells with both **Ir4a** and **NC-Ir4a**. This suggests that cells failed to activate compensatory glycolysis in response to impaired mitochondrial oxidative phosphorylation (OXPHOS). This glycolytic suppression was absent in the dark-treated and cisplatin-treated cells, reinforcing the idea that photoactivated compounds induce a unique form of bioenergetic collapse. These OCR/ECAR profiles collectively indicate that **Ir4a** and **NC-Ir4a** exert the most severe disruption of mitochondrial and glycolytic energy pathways, suggesting a severe collapse of cellular energy metabolism.

Accordingly, the JC-1 staining assay (Figure 11B) demonstrated a significant reduction in mitochondrial membrane potential (MMP) upon treatment with photoactivated **Ir4a** and **NC-Ir4a**, with **NC-Ir4a** exerting a more pronounced effect. MMP is a key indicator of mitochondrial health, reflecting the electrochemical gradient essential for ATP synthesis.^77^ This disruption of mitochondrial stability suggests impaired electron transport chain (ETC) activity, leading to a loss of energy homeostasis. The mitochondrial ETC inhibitor antimycin A (AMA) induced similar MMP dissipation, reinforcing the notion that **Ir4a** and **NC-Ir4a**-mediated phototherapy severely compromises mitochondrial function.

The suppression of OCR aligns with lipid peroxidation-associated mitochondrial damage, a key feature of ferroptosis.^78,79^ This is further supported by intracellular ATP depletion shown in Figure 11C, which underscores a metabolic collapse characteristic of nonapoptotic cell death. Unlike apoptosis, which requires ATP for caspase activation and membrane blebbing, ferroptosis is driven by oxidative mitochondrial damage and iron-dependent lipid peroxidation. Consistently, cisplatin-treated cells—serving as an apoptotic control—exhibited only a moderate ATP reduction, highlighting the distinct metabolic dysfunction induced by **Ir4a** and **NC-Ir4a**-mediated PDT.

Overall, this pattern of profound OCR suppression, glycolytic failure, MMP loss and ATP depletion aligns well with the metabolic features of ferroptosis. Unlike apoptosis, which maintains mitochondrial structure and relies on ATP for caspase activity, ferroptosis is marked by mitochondrial damage, lipid peroxidation, and energetic failure. The enhanced potency of **NC-Ir4a** suggests that nanoencapsulation amplifies mitochondrial damage and oxidative stress propagation of **Ir4a**, further reinforcing its role in ferroptosis induction.

### Phototoxicity of Ir-COUBPY Complexes in 3D Tumor Spheroids

The photocytotoxic activity of **Ir4a** and its nanoencapsulated form (**NC-Ir4a**) was evaluated using three-dimensional multicellular tumor spheroids (MCTS), a model that more accurately mimics the structural and physiological complexity of solid tumors.^80^ The effects of **Ir4a** and **NC-Ir4a** on the growth of A375 melanoma spheroids were assessed under both dark conditions and green-light irradiation. Notably, light exposure significantly reduced spheroid volume in both **Ir4a**- and **NC-Ir4a**-treated groups compared to untreated controls (Figure 12A). This inhibitory effect persisted throughout the observation period, with pronounced spheroid shrinkage evident by day 8 (Figure 12B), indicating robust suppression of tumor growth.

**Figure 12:**
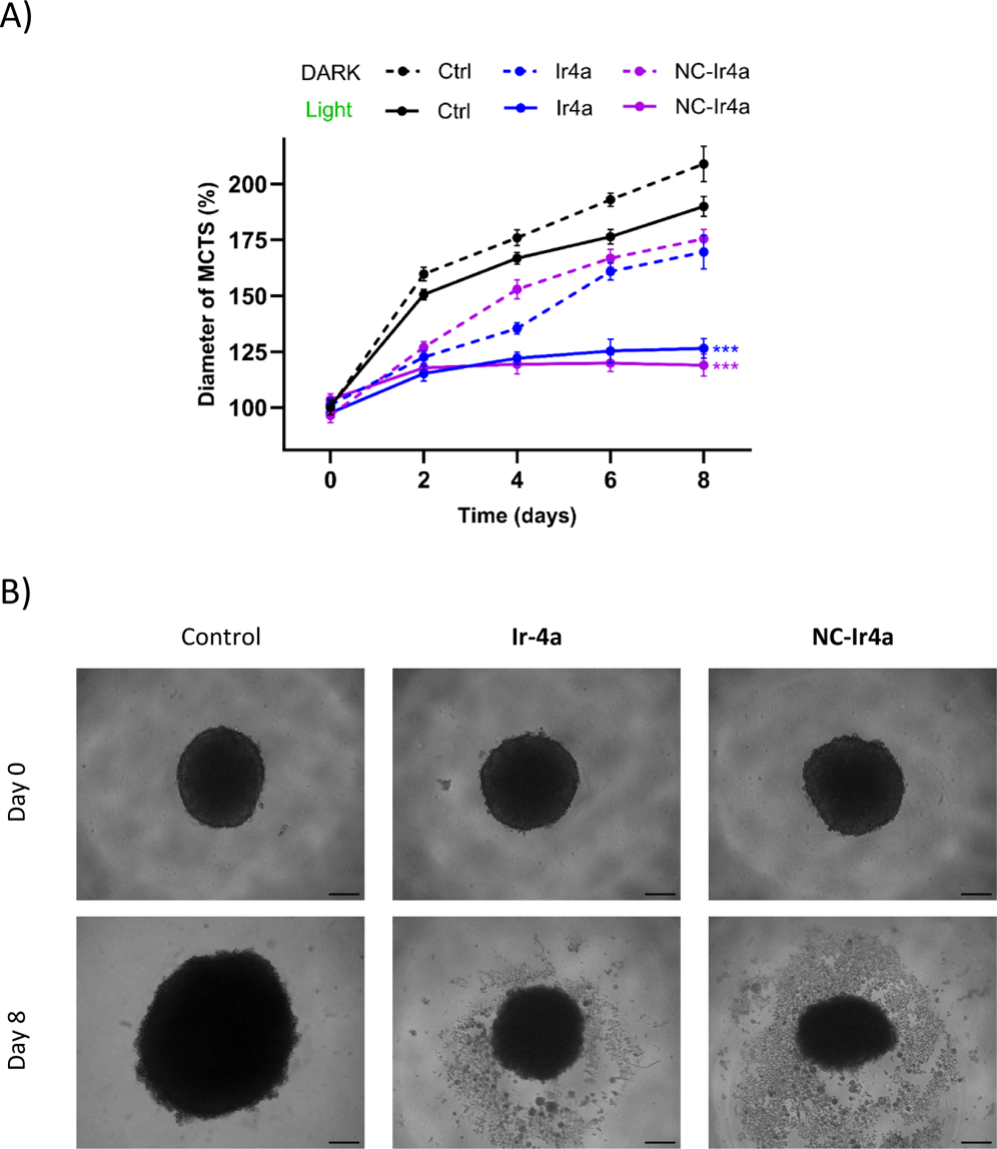
(A) Normalized diameter of A375 multicellular tumor spheroids (MCTS) treated with **Ir4a** or **NC-Ir4a** (2.5 μM, treated every 2 days) under dark or green light irradiation over an 8-day period. Data are mean ± SD (*n* = 4 spheroids per treatment). Statistical analysis was performed at day 8 using one-way ANOVA versus the respective control (dark or light) (*p < 0.05; **p < 0.01; ***p < 0.001). (B) Morphology of irradiated spheroids observed on days 0 (before treatment) and 8. Scale bar: 200 μm.

To further investigate cell death mechanism, a subset of spheroids was subjected to dual staining with calcein-AM and propidium iodide on day 4. As shown in Figure 13, treatment with **Ir4a** and **NC-Ir4a** under green light irradiation led to a substantial decrease in calcein fluorescence, indicating a loss of viable cells. Concurrently, a notable increase in propidium iodide fluorescence was observed, indicating loss of membrane integrity consistent with ferroptotic or similar lytic forms of cell death. These fluorescence shifts provide strong evidence of enhanced phototoxicity mediated by Ir-COUBPY complex **Ir4a**, corroborating the observed tumor growth inhibition.

**Figure 13:**
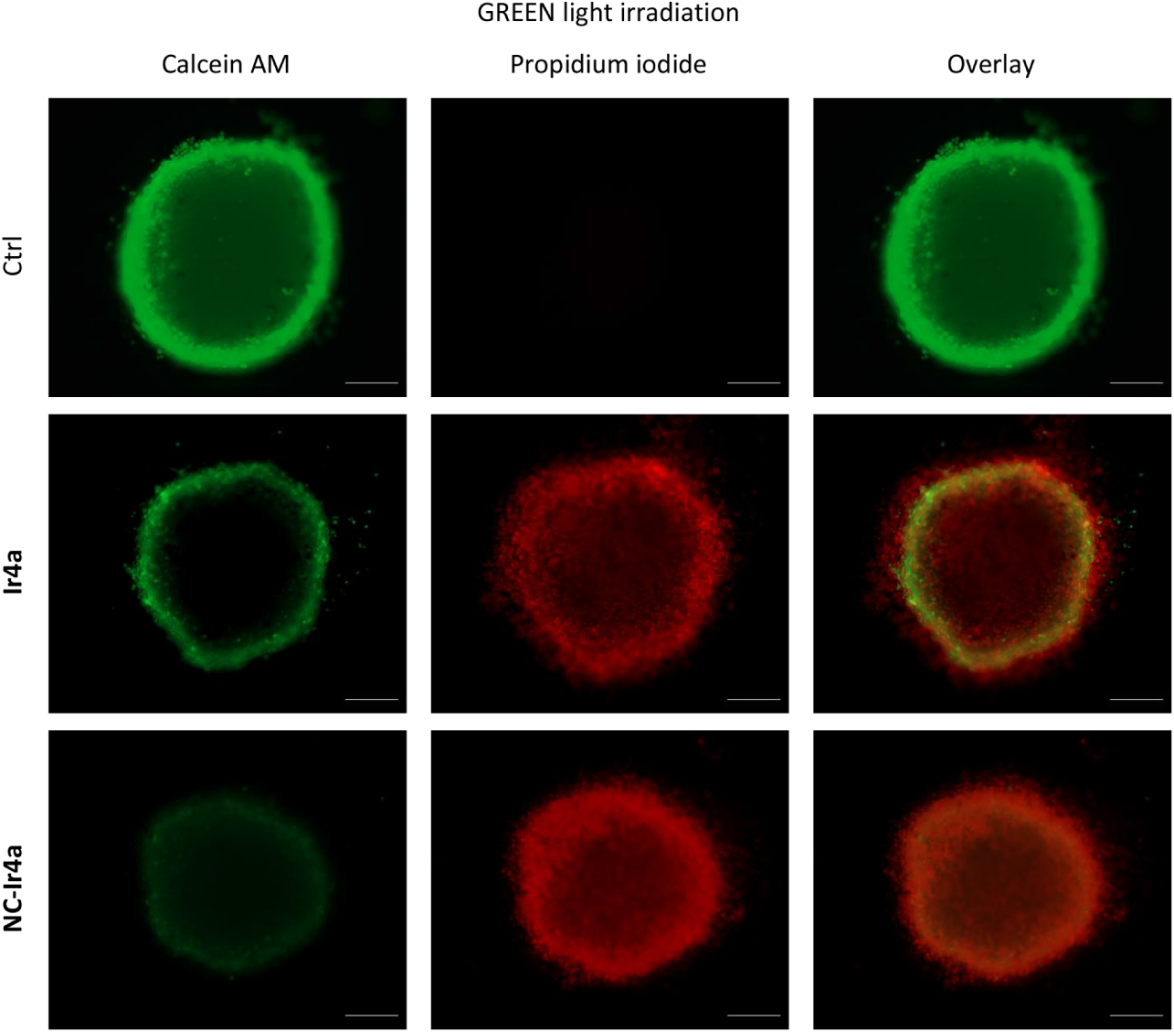
Fluorescence microscopy analysis of A375 MCTS treated with **Ir4a** or **NC-Ir4a** (2.5 μM) for 1 h, followed by 1 h of green light irradiation and a 4-day incubation. A second treatment was administered on day 2. Spheroids were stained with calcein-AM (10 μM) and propidium iodide (2 μg/mL) to assess cell viability and membrane integrity. Identical treatments were performed under dark conditions as controls. Scale bar: 200 μm.

Finally, to evaluate ROS generation, spheroids were stained with DHE,^8,9^ under both dark and light conditions. As shown in Figure S74, DHE fluorescence was significantly elevated in light-irradiated spheroids treated with either **Ir4a** or **NC-Ir4a**, compared to their respective dark controls. These results confirm the light-triggered production of ROS within the 3D tumor spheroids, which closely mimic the *in vivo* tumor microenvironment.

## Conclusions

In summary, a series of novel heteroleptic cyclometalated Ir­(III) complexes, combining five different C^N ligands and an N^N COUBPY ligand, **Ir1a–Ir5a**, were synthesized to develop new visible-light-sensitive PSs. Ir-COUBPY complexes exhibited significant red-shifted absorption and emission compared to their Ir-bpy counterparts, **Ir1b–Ir5b**, and demonstrated chemical stability in the dark and photostability under physiological-like conditions. Upon visible light irradiation, Ir-COUBPY PSs generated both Type I and Type II ROS as demonstrated using spectroscopic techniques, including EPR, and were capable of photooxidize NADH.

Photocytotoxicity studies against HeLa and A375 cancer cells confirmed the crucial role of the COUBPY ligand in enhancing PDT efficiency. Specifically, Ir-COUBPY complexes showed reduced dark cytotoxicity compared to their bpy analogues, resulting in significantly higher PI values and nanomolar activity under green light irradiation (e.g., PI = 64 for **Ir4a** vs PI = 5.4 for **Ir4b** in A375 cells). Notably, Ir-COUBPY complexes retained high phototoxicity under red light irradiation (620 nm), offering promising potential for treating large solid tumors. The most effective PS, **Ir4a**, was encapsulated into polyurethane–polyurea hybrid nanocapsules (**NC-Ir4a**), leading to a remarkable increase in PI values (from 64 to 179.6 in A375 cells). Ir-COUBPY complexes preferentially accumulated in the mitochondria of cancer cells, inducing the photogeneration of hydroxyl radicals and, to a lesser extent, hydrogen peroxide.

Mechanistic studies confirmed ferroptosis as the primary cell death pathway induced by **Ir4a**, with enhanced effects observed upon nanoencapsulation in polyurethane–polyurea nanocapsules. This was supported by light-dependent lipid peroxidation (BODIPY-C11), glutathione oxidation and depletion (GSH/GSSG ratio and GSH quantification), intracellular ATP photodepletion, and the viability-restoring effect of Fer-1. Additionally, **Ir4a** and **NC-Ir4a** induced photosuppression of the oxygen consumption rate, mitochondrial membrane potential loss, and bioenergetic collapse, further supporting ferroptotic cell death. The absence of apoptotic markers such as γH2AX phosphorylation and membrane blebbing reinforced the nonapoptotic nature of the response. Notably, photobiological studies with 3D tumor spheroids of A375 cells, which better mimic the *in vivo* tumor microenvironment, confirmed enhanced cellular uptake of **NC-Ir4a** compared to **Ir4a**, likely contributing to the improved phototoxic efficiency observed. Overall, these findings support the potential of coumarin-based COUBPY ligands for the rational design of new Ir­(III)-based PSs activatable with light within the phototherapeutic window and operating via nonconventional cell death mechanisms, such as ferroptosis.

## Supplementary Material



## Data Availability

The data supporting this article have been included as part of the Supporting Information.

## Notes

Full experimental procedures: synthesis, photophysical and photochemical characterization of the compounds, cellular uptake and *in vitro* photobiological studies; copies of HPLC traces, NMR and HRMS spectra; schemes S1–S3, figures S1–S74 and tables S1–S15 (PDF)
