# Supplementary material for: Novel Heteroleptic Iridium(III) Complexes Containing COUBPY Ligands for Effective Photoinduction of Ferroptosis for Cancer Therapy
Source: JACS Au. 2025 Dec 20;6(3):1585–601. doi: 10.1021/jacsau.5c01441 (PMC13014250; doi:10.1021/jacsau.5c01441)
Supplement: Supplementary file 1 [file au5c01441_si_001.pdf]

Supporting Information

Novel Heteroleptic Iridium(III) Complexes Containing COUBPY Ligands for Effective Photoinduction of Ferroptosis for Cancer Therapy

Pezhman Ashoo,[a,#] Alba Hernández-García,[a,#] Eduardo Izquierdo-García,[b] Neus Santiago,[b,c] Rebeca Mondaray-Marín,[a] Diego Abad-Montero,[b] Manel Bosch,[d] Neus Isidro,[c] Valentin V. Novikov,[e] Josep Rocas,[c] María Dolores Santana,[a,*] Jose Ruiz,[a,*] and Vicente Marchán[b,e*]

[a]*Departamento de Química Inorgánica, Universidad de Murcia, and Murcia BioHealth Research Institute (IMIB-Arrixaca), E-30100 Murcia, Spain. E-mail: jruiz@um.es, dsl@um.es*

[b]*Departament de Química Inorgànica i Orgànica, Secció de Química Orgànica, Universitat de Barcelona (UB), Institut de Biomedicina de la Universitat de Barcelona (IBUB), E-08028 Barcelona, Spain. E-mail: vmarchan@ub.edu*

[c]*Ecopol Tech S.L., Nanobiotechnological Polymers Division, R&D Department, E-43720 L'Arboç del Penedès, Tarragona, Spain*

[d]*Unitat de Microscòpia Òptica Avançada, Centres Científics i Tecnològics, Universitat de Barcelona, E-08028 Barcelona, Spain*

[e]*Departament de Química Inorgànica i Orgànica, Secció de Química Inorgànica, Universitat de Barcelona (UB), and Institute of Nanoscience and Nanotechnology of the University of Barcelona (IN2UB), Martí i Franquès 1-11, E-08028 Barcelona, Spain.*

[f]*Professor Serra Húnter, Universitat de Barcelona, E-08028 Barcelona, Spain*

# These authors contributed equally to this work.

Table of contents

1. Experimental section S3
2. Synthetic procedures S15
3. NMR spectroscopy S16
4. High Performance Liquid Cromatography-Mass spectrometry analysis S26
5. Photophysical properties..S34
6. Dark and light stability studies..S37
7. NADH photo-oxidation and evaluation for $^1O_2$ and/or •OH/ $^•O_2^-$ generation in cell free media S48
8. Cellular uptake studies by confocal microscopy of Ir-COUBPY and Ir-bpy complexes S57
9. Partition coefficients (log P) S60
10. Synthesis and characterization of NC-Ir4a..S62
11. Biological assays S67
12. References S75

1. Experimental section

Reagents

2,2'-bipyridine, 2-phenylpyridine, 2-phenylbenzimidazole, 2-phenylbenzothiazole, thiophene-2-carboxaldehyde, potassium triflate, dimethylsulfoxide (DMSO), 2-ethoxyethanol, 4-trifluoromethylphenylboronic acid, 2-aminothiophenol, 2-thiophenecarboxaldehyde, sodium bisulfite, tetrakis(triphenylophosphine)palladium(0) were obtained from Merck (Madrid, Spain). $IrCl_3 \cdot H_2O$ was obtained from Johnson Matthey. Deuterated solvents were obtained from Eurisotop. The purities ≥95 % of the synthesized complexes used for biological evaluation were determined by ^1H NMR spectroscopy and by RP-HPLC and elemental analysis after changing triflate to chloride due to solubility criteria.

Synthetic procedures

COUBPY ligand was synthesized following a reported procedure [Scheme S1].[1] **HL4** and **HL5** were synthesized following previously described procedures with slight modifications (see Scheme 2 and Supporting Information for further details).

Synthesis of dimer complexes $[Ir(C^\wedge N)_2(\mu\text{-}Cl)]_2$

The dimeric iridium(III) precursors were synthesized as previously described (Scheme S3),[2, 3] by reacting $IrCl_3 \cdot H_2O$ (50 mg, 0.16 mmol) with the corresponding proligand (**HL1**-**HL5**) (0.35 mmol) in 8 mL of 2-etoxiethanol/H_2O (3:1 v:v) mixture at 110 ºC under a nitrogen atmosphere for 24 h. The reaction mixture was then cooled to room temperature, filtered, and washed with water. The resulting products were used in subsequent reactions without further purification.

Synthesis of iridium complexes $[Ir(C^\wedge N)_2(N^\wedge N)]OTf$

The corresponding dimeric iridium(III) precursor (1 mol eq), the corresponding N^N ligand (bpy or COUBPY, 2 mol eq) and potassium triflate (2.5 mol eq) were dissolved in 10 mL of MeOH/DCM (3:2 v:v) and stirred at 58 ºC for 24 h. Upon completion of the reaction, the mixture was cooled to room temperature, and the solvent was removed under reduced pressure. The pure products were isolated via alumina column chromatography, employing a 1:1 mixture of DCM and CH_3CN as the eluent. Finally, Ir-bpy and Ir-COUBPY complexes were recrystallized with DCM and hexane, followed by multiple washes with hexane to yield the final pure iridium complex.

Ir1a. Red solid. Isolated yield: 40 %. ^1H NMR (401 MHz, DMSO-d_6) δ (ppm): 8.77-8.69 (m, 1H), 8.65-8.58 (m, 1H), 8.34-8.22 (m, 2H), 8.06-7.99 (m, 1H), 7.99-7.88 (m, 4H), 7.85 (d, J = 6.2 Hz, 1H), 7.77 (d, J = 5.8 Hz. 1H), 7.70 (d, J = 5.6 Hz, 1H), 7.64 (d, J = 5.9 Hz, 1H), 7.60-7.48 (m, 2H), 7.26-7.14 (m, 2H), 7,01 (t, J = 7.5 Hz, 2H), 6.96 – 6.86 (m, 3H), 6.86 – 6.74 (m, 2H), 6.19 (dd, J = 11.0, 7.4 Hz, 2H), 3.47 (q, J = 7.7 Hz, 4H), 2.52 (s, 3H), 2.42 (s, 3H), 1.12 (t, J = 7.0 Hz, 6H). ^{13}C RMN (101 MHz, DMSO-d_6) δ (ppm): 167.0, 166.9, 165.0, 155.1, 154.9, 154.4, 151.4, 151.2, 151.1, 150.7, 149.8, 149.0, 148.8, 143.9, 143.7, 138.7, 138.6, 131.2, 131.0, 130.2, 129.3, 126.4, 125.3, 125.1, 125.0, 124.0, 123.9, 123.5, 122.2, 122.1, 120.0, 119.8, 119.1, 118.9, 110.6, 110.5, 109.6, 97.2, 78.6, 43.9, 20.9, 18.2, 12.5. HPLC-MS (pos. ion. mode): calc.. $[M-Cl]^+$= 923.3049 m/z; exp: 923.3084 $[M-Cl]^+$ m/z. Anal. Calc. for $C_{49}H_{42}ClIrN_6O$: %C. 61.40; %H. 4.42; %N. 8.77.

Found: %C. 61.10; %H. 4.50; %N. 8.80.

Ir2a: 37 %. ^{1}H NMR (401 MHz, DMSO-*d*$_6$) δ (ppm): 13.89 (s, 2H), 8.78-8.72 (m, 1H), 8.65-8.60 (m, 1H), 8.10 (d, *J* = 6.2 Hz, 1H), 8.06 (s, 1H), 8.02 (dd, *J* = 6.1, 2.1 Hz, 1H), 7.96 (d, *J* = 5.6 Hz, 1H), 7.90-7.84 (m, 2H), 7.64 – 7.58 (m, 1H), 7.58 – 7.54 (m, 1H), 7.53-7.48 (m, 1H), 7.27-7.19 (m, 2H), 7.07 – 6.89 (m, 4H), 6.87 – 6.73 (m, 5H), 6.24 (dd, *J* = 14.9, 7.5 Hz, 2H), 5.95 (d, *J* = 8.3 Hz, 1H), 5.72 (d, *J* = 8.3 Hz, 1H), 3.47 – 3.40 (m, 4H), 2.55 (s, 3H), 2.40 (s, 3H), 1.09 (t, *J* = 7.0 Hz, 6H). ^{13}C RMN (101 MHz, DMSO-*d*$_6$) δ (ppm): 164.9, 164.3, 164.2, 156.2, 156.1, 154.3, 151.2, 151.1, 150.8, 150.7, 150.1, 148.6, 143.5, 139.4, 139.3, 134.2, 133.2, 133.2, 132.7, 132.6, 130.4, 128.9, 126.3, 124.7, 124.3, 123.6, 123.5, 123.2, 123.2, 122.3, 121.9, 121.9, 119.1, 118.8, 113.1, 112.9, 110.5, 110.5, 109.6, 97.1, 79.0, 78.6, 54.9, 43.9, 20.9, 18.1, 12.4. HPLC-MS (pos. ion. mode): calc., [M-Cl]$^+$= 1001.3267 m/z; exp: 1001.3290 [M-Cl]$^+$ m/z. Anal. Calc. for $C_{53}H_{44}ClIrN_8O$: %C, 61.41; %H, 4.28; %N, 10.81. Found: %C, 61.14; %H, 4.43; %N, 10.90.

Ir3a: 32 %. ^{1}H NMR (401 MHz, DMSO-*d*$_6$) δ (ppm): 8.74-8.69 (m, 1H), 8.67-8.62 (m, 1H), 8.24 (d, *J* = 8.1 Hz, 2H), 8.08 (dd, *J* = 6.2, 2.1 Hz, 1H), 8.04 – 7.95 (m, 3H), 7.86 (d, *J* = 5,7 Hz, 1H), 7.64 – 7.58 (m, 1H), 7.54 (d, *J* = 8.7 Hz, 1H), 7.48 – 7.39 (m, 2H), 7.32-7.25 (m, 1H), 7.23-7.16 (m, 1H), 7.15-7.07 (t, *J* = 7.6, 2H), 6.92 (t, *J* = 7.6 Hz, 2H), 6.84 – 6.75 (m, 3H), 6.45 (d, *J* = 8.4 Hz, 1H), 6.29 (dd, *J* = 13.8, 7.6 Hz, 2H), 6.19 (d, *J* = 8.4 Hz, 1H), 3.51 – 3.40 (m, 4H), 2.55 (s, 3H), 2.42 (s, 3H), 1.10 (t, *J* = 7.0 Hz, 6H). ^{13}C RMN (151 MHz, DMSO-*d*$_6$) δ (ppm): 181.2, 181.1, 165.2, 155.8, 155.6, 154.4, 152.2, 151.2, 150.8, 150.0, 149.4, 149.1, 148.7, 144.3, 140.1, 132.7, 132.6, 132.1, 131.3, 131.3, 129.6, 128.3, 128.1, 127.1, 127.0, 126.4, 126.1, 126.0, 125.2, 124.7, 123.6, 123.0, 123.0, 119.4, 118.7, 117.2, 116.9, 110.7, 110.5, 109.7, 97.1, 78.5, 43.9, 21.0, 18.2, 12.4. HPLC-MS (pos. ion. mode): calc., [M-Cl]$^+$= 1035.2491 m/z; exp: 1035.2533 [M-Cl]$^+$ m/z. Anal. Calc. for $C_{53}H_{42}ClIrN_6OS_2$: %C, 59.45; %H, 3.95; %N, 7.85; %S, 5.99. Found: %C, 59.14; %H, 4.05; %N, 7.75; %S, 5.89.

Ir4a: 30 %. ^{1}H NMR (600 MHz, DMSO-*d*$_6$) δ (ppm): 8.74-8.70 (m, 1H), 8.65 – 8.62 (m, 1H), 8.15 – 8.11 (m, 2H), 8.08 (dd, *J* = 6.3, 2.2 Hz, 1H), 7.92 (d, *J* = 6.3 Hz, 1H), 7.82 (dd, *J* = 4.7, 2.2 Hz, 2H), 7.79 (d, *J* = 5.7 Hz, 1H), 7.66 – 7.62 (m, 1H), 7.53 (d, *J* = 9.0 Hz, 1H), 7.38-7.32 (m, 2H), 7.26-7.21 (m, 1H), 7.17-7.12 (m, 1H), 6.84-6.75 (m, 3H), 6.37 – 6.33 (m, 1H), 6.15 (dd, *J* = 13.5, 4.7 Hz, 2H), 6.11 – 6.07 (m, 1H), 3.49 – 3.43 (m, 4H), 2.57 (s, 3H), 2.41 (s, 3H), 1.10 (t, *J* = 7.0 Hz, 6H). ^{13}C NMR (151 MHz, DMSO-*d*$_6$) δ (ppm): 174.0, 173.9, 165.2, 160.0, 159.6, 155.8, 155,6, 154.4, 152.4, 151.2, 150.3, 149.8, 149.2, 148.9, 144.4, 136.2, 132.1, 132.0, 131.9, 131.8, 131.2, 131.2, 129.6, 128.2, 128.0, 126.4, 125.2, 125.1, 125.0, 124.6, 123.5, 121.8, 119.6, 119.3, 118.7, 116.0, 115.7, 110.7, 110.5, 109.7, 97.1, 78.5, 43.9, 21.0, 18.2, 12.4. HPLC-MS (pos. ion. mode): calc., [M-Cl]$^+$= 1047.1619 m/z; exp: 1047.1643 [M-Cl]$^+$ m/z. Anal. Calc. for $C_{49}H_{38}ClIrN_6OS_4$: %C, 54.35; %H, 3.54; %N, 7.76; %S, 11.85. Found: %C, 54.24; %H, 3.58; %N, 7.72; %S, 11.81.

Ir5a: 35 % ^{1}H NMR (401 MHz, DMSO-*d*$_6$) δ (ppm): 8.77-8.74 (m, 1H), 8.67-8.62 (m, 1H), 8.21-8.14 (m, 2H), 8.08-8.05 (m, 2H), 8.00 (d, *J* = 5.7 Hz, 1H), 7.82-7.75 (m, 4H), 7.74 – 7.68 (m, 4H), 7.67 – 7.62 (m, 1H), 7.59 (d, *J* = 9.0 Hz, 1H), 7.37 (t, *J* = 7.7 Hz, 2H), 7.24 (ddd, *J* = 8.6, 7.4, 1.3 Hz, 1H), 7.14 (ddd, *J* = 8.5, 7.2, 1.3 Hz, 1H), 6.89 – 6.74 (m, 5H), 6.34 (d, *J* = 8.3 Hz, 1H), 6.08

(d, J = 8.4 Hz, 1H), 3.46 (q, J = 7.6 Hz, 4H), 2.57 (s, 3H), 2.45 (s, 3H), 1.10 (t, J = 7.0 Hz, 6H). ^{13}C NMR (151 MHz, DMSO-d_6) δ (ppm): 173.3, 173.2, 165.3, 160.6, 160.2, 155.8, 155.6, 154.3, 152.6, 151.2, 150.7, 150.6, 150.3, 149.9, 149.4, 148.9, 144.6, 136.3, 133.4, 131.7, 131.6, 130.0, 129.3, 129.7, 128.8, 128.5, 128.2, 128.0, 126.8, 126.4, 126.1, 125.2, 124.9, 124.7, 123.7, 123.1, 119.3, 118.6, 116.0, 115.7, 110.8, 110.5, 109.7, 97.1, 78.4, 43.9, 21.0, 18.2, 12.4. HPLC-MS (pos. ion. mode): calc., [M-Cl]$^+$= 1335.1993 m/z; exp: 1335.2033 [M-Cl]$^+$ m/z. Anal. Calc. for $C_{63}H_{44}ClF_6IrN_6OS_4$: %C, 55.19; %H, 3.23; %N, 6.13; %S, 9.36. Found: %C, 55.27; %H, 3.31; %N, 6.02; %S, 9.30.

Ir2b: 42 %. ^{1}H NMR (401 MHz, DMSO-d_6) δ (ppm) 13.92 (s, 2H), 8.85 (d, J = 8.2 Hz, 2H), 8.33-8.24 (m, 2H), 8.15 (d, J = 5.4 Hz, 2H), 7.88 (d, J = 7.6 Hz, 2H), 7.75 (t, J = 6.6 Hz, 2H), 7.60 (d, J = 8.1 Hz, 2H), 7.23 (t, J = 7.7 Hz, 2H), 7.04 (t, J = 7.5 Hz, 2H), 6.92 (t, J = 7.8 Hz, 2H), 6.88 – 6.80 (m, 2H), 6.24 (d, J = 7.6 Hz, 2H), 5.60 (d, J= 8.2 Hz, 2H). ^{13}C NMR (101 MHz, DMSO-d_6) δ (ppm): 164.3, 156.5, 151.0 150.4, 139.5, 139.2, 134.1, 133.2, 132.6, 130.5, 128.4, 124.3, 123.6, 123.3, 122.1, 113.2, 112.7. HPLC-MS (pos. ion. mode.): calc., [M-Cl]$^+$= 735.1848 m/z; exp: 735.1859 [M-Cl]$^+$ m/z. Anal. Calc. for $C_{36}H_{26}ClIrN_6$: %C, 56.13; %H, 3.40; %N, 10.91. Found: %C, 56.08; %H, 3.55; %N, 10.62.

Ir4b: 23 %. ^{1}H NMR (401 MHz, DMSO-d_6) δ (ppm): 8.88-8.82 (m, 2H), 8.33 (dt, J = 7.9, 1.6 Hz, 2H), 8.17 – 8.10 (m, 2H), 8.01-7.96 (m, 2H), 7.87 – 7.78 (m, 4H), 7.35 (ddd, J = 8.3, 7.3, 1.1 Hz, 2H), 7.13 (ddd, J = 8.5, 7.3, 1.3 Hz, 2H), 6.17 (d, J = 4.7 Hz, 2H), 6.01-5.96 (m, 2H).^{13}C NMR (101 MHz, DMSO-d_6) δ (ppm): 174.1, 159.2, 156.0, 150.8, 148.7, 140.6, 136.3, 132.1, 131.8, 131.2, 129.1, 128.0, 125.1, 124.8, 124.7, 115.5. HPLC-MS (pos. ion. mode): calc., [M-Cl]$^+$= 781.0200 m/z; exp: 781.0211 [M-Cl]$^+$ m/z. Anal. Calc. for $C_{32}H_{20}ClIrN_4S_4$: %C, 47.08; %H, 2.47; %N, 6.86; %S, 15.71. Found: %C, 46.74; %H, 2.62; %N, 6.69; %S, 15.82.

Ir5b: 20 %. ^{1}H NMR (600 MHz, $CDCl_3$) δ (ppm): 9.12 – 9.01 (m, 2H), 8.30 (dt, J = 7.9, 1.6 Hz, 2H), 8.15 – 8.11 (m, 2H), 7.85 – 7.79 (m, 2H), 7.61 – 7.51 (m, 10H), 7.36-7.31 (m, 2H), 7.12-7.07 (m, 2H), 6.49 (s, 2H), 6.03 (d, J = 8,4 Hz, 2H). ^{13}C NMR (151 MHz, $CDCl_3$) δ (ppm): 173.3, 160.0, 157.0, 152.6, 150.7, 149.2, 141.2, 136.5, 133.7, 131.4, 130.9, 130.7, 129.4, 128.8, 128.4, 126.8, 126.5, 126.1, 126.1, 125.8, 123.7, 116.5. HPLC-MS (pos. ion. mode): calc., [M-Cl]$^+$= 1069.0568 m/z; exp: 1069.0559 [M-Cl]$^+$ m/z. Anal. Calc. for $C_{46}H_{26}ClF_6IrN_4S_4$: %C, 50.02; %H, 2.37; %N, 5.07; %S, 11.61. Found: %C, 50.08; %H, 2.39; %N, 5.12; %S, 11.50.

Methods and Instrumentation

Nuclear Magnetic Resonance (NMR) Spectroscopy

The ^{1}H-NMR and ^{13}C-NMR spectra were recorded in Bruker NMR spectrometers AV400 and AV600, and chemical shifts were determined by reference to the residual ^{1}H and ^{13}C{^{1}H} solvent peaks.

Elemental Analysis

Elemental analysis of C, H, N and S was determined using a Carlo Erba Model EA 1108 microanalyzer equipped with EAGER 200 sofware.

High Performance Liquid Chromatography-Mass Spectrometry

HPLC-MS (positive mode) analysis were carried out in an Agilent HPLC 1290 Series II coupled with an Agilent iFunnel 6550 QTOF AJS Dual ESI, using a Zorbax Eclipse Plus C18 column (2.1 x 100 mm, 1.8 μm). The flow rate was set at 0.4 mL/min. The isotopic distribution of the heaviest set of peaks matched very closely to that calculated for formulating the complex cation in every case.

Photophysical Characterization

UV/vis spectroscopy was performed on a PerkinElmer Lambda 750 S spectrometer with operating software. Solutions of all complexes were prepared in acetonitrile and water (1% DMSO) at 10 μM. The emission spectra were obtained with a Horiba Jobin Yvon Fluorolog 3-22 modular spectrofluorometer with a 450 W xenon lamp. Measurements were performed in a right-angled configuration using 10 mm quartz fluorescence cells for solutions at 298 K. Emission lifetimes were measured using an IBH FluoroHub TCSPC controller and a NanoLED (372 nm) pulse diode excitation source (τ <10 μs); the estimated uncertainty is ±10% or better. Emission quantum yields (Φ) were determined using a Hamamatsu C11347 absolute PL quantum yield spectrometer; the estimated uncertainty is ± 10% or better. For lifetimes and quantum yield measurements, the samples in acetonitrile were previously degassed by bubbling argon for 30 min.

Stability in biological medium

Solutions of the Ir complexes (30 μM) were prepared in RPMI culture medium supplemented with 10% fetal bovine serum (FBS), 100 U·mL^{-1} penicillin-streptomycin, and 10% DMSO. The solutions also contained 2,3,6,7-tetrahydro-1,1,7,7,9-pentamethyl-1H,5H,11H-[1]benzopyrano[6,7,8-ij]quinolizin-11-one (30 μM) as an internal standard. Samples were stirred in a thermomixer at 600 rpm and 37 °C for 4 h. Aliquots (50 μL) were collected at the indicated time points (0, 1, and 4 h) and analyzed by reversed-phase HPLC. The degradation of the photosensitizer was assessed by comparing the ratio of the Ir complex peak area to that of the internal standard at each time point.

The HPLC analysis was performed with a Waters alliance 2695 Separations Module, comprised of a quaternary pump solvent delivery module, online degasser, auto sampler and a Waters 2996 photodiode array detector. HPLC separation was carried out using a Jupiter Proteo C12 column

(50 x 4.6 mm, 90 Å, 4 µm) from Phenomenex. Samples were eluted using a linear gradient from 90:10 to 0:100 (v/v) A/B over 7 min at 1.5 mL·min^{-1} (A: 0.1% formic acid in H_2O; B: 0.1% formic acid in ACN). For complex **5a**, a 40:60 to 0:100 (v/v) A/B gradient was used. The injection volume was 20 µL. Control of the HPLC instrument, as well as processing of the chromatogram output (annotation of retention times, integration of peaks, calculation of peak areas) was carried out with MassLynx V4.1 software.

NADH photooxidation

The reaction between the iridium complexes and NADH was monitored using UV/Vis absorption spectroscopy in the dark and after irradiation with green light (520 nm, 2.0 mW/cm^2), or red light (620 nm, 15 mW/cm^2). For this purpose, a solution of NADH (100 µM) in PBS (5% DMF) was prepared in the presence of the respective compound (5 µM), and the UV/Vis absorption spectrum was recorded at different times. The turnover number (TOF) was calculated as the ratio of the concentration of oxidized NADH to the product of the concentration of the compound used and the reaction time. The concentration of NADH was determined using the molar extinction coefficient at 339 nm (ε_{339}= 6220 M^{-1}cm^{-1}).

Superoxide radical generation

The formation of superoxide ($^{\bullet}O_2^-$) radical anion during the catalytic photooxidation of NADH was evaluated by a colorimetric assay with nitroblue tetrazolium chloride (NBT) probe. All compounds (5 µM), NADH (100 µM), and NBT (50 µM) were solved in a H_2O/DMF (95:5) mixture. The absorption of the progressively formed formazan blue (λ_{abs} = 590 nm) was monitored upon short irradiation intervals with green light (520 nm, 1.0 mW/cm^2).

Singlet oxygen quantum yield

Singlet oxygen quantum yields were determined in aerated acetonitrile solution using DPBF as a chemical trap upon green light irradiation. DPBF solutions (50 µM) in acetonitrile were prepared in the presence of the complexes at known concentrations or the reference [Ru(bpy)$_3$]Cl$_2$. The photolysis of DPBF was monitored using UV/Vis absorption spectroscopy (350-500 nm) after irradiation with green light (520 nm, 0.5 mW/cm^2). Finally, the absorbance of DPBF at 411 nm was plotted against irradiation times and the singlet oxygen quantum yields were calculated by using the following equation:

$$\Phi\Delta s = \Phi\Delta \mathrm{r}\, \frac{m_s}{m_r} \frac{(1-10^{\mathrm{A\lambda r}})}{(1-10^{\mathrm{A\lambda s}})}$$

where $\Phi\Delta_r$ is the singlet oxygen quantum yield of the reference, as said [Ru(bpy)$_3$]Cl$_2$ ($\Phi_{\Delta r}$ = 0.57 in acetonitrile), m are the slopes of complexes and the reference and $A_{\lambda s}$ and $A_{\lambda r}$ are the absorbance of the compounds and of the reference at the irradiation wavelength, respectively.

Hydroxyl radical generation

All compounds (10 µM) and the non-fluorescent probe HPF (10 µM) were prepared in PBS (5% DMF). The samples were then irradiated with green light (520 nm, 2.0 mW/cm^2) and the evolution

of the HPF emission spectra (λ_{ex}= 490 nm) was recorded over time. The excitation and emission slit widths were set at 3 nm, and the fluorescence emission spectra were recorded in the wavelength range of λ= 500-600 nm. Fluorescence spectra were obtained with a Horiba Jobin Yvon Fluorolog 3-22 modular spectrofluorometer with a 450 W xenon lamp. Measurements were performed in a right-angled configuration using 10 mm quartz fluorescence cells for solutions at 298 K.

Electron paramagnetic resonance (EPR).

EPR experiments were carried out using the **Ir4a** complex (250 μM), which was dissolved in MeOH containing either 120 mM 4-amino-TEMP (4-amino-2,2,6,6-tetramethylpiperidine) as a spin trap for singlet oxygen ($^1O_2$), or 360 mM DMPO (5,5-dimethyl-1-pyrroline-N-oxide) as a spin trap for superoxide ($^{\bullet}O_2^{-}$). The resulting samples were loaded into Hirschmann 50 μL capillary tubes (both ends open) and sealed with Critoseal®.

EPR measurements were performed on a Bruker Elexsys 580 spectrometer operating in the X-band at room temperature, using a Bruker ER4122 SHQE super-high-Q cylindrical resonator in continuous-wave (CW) mode. Spectra were recorded both in the dark and after white-light irradiation with an Asahi Spectra MAX-303 xenon light source (300 W) equipped with 520 nm or 580 nm narrow-pass filter. The light was delivered to the EPR cavity via a quartz light guide through the optical window of the ER4122 SHQE resonator. The microwave frequency was 9.858 GHz, with a modulation amplitude of 0.03 mT and a microwave power of 4.7 mW. These parameters were selected to avoid signal distortion or saturation.

The superoxide quantum yield ($\Phi^{\bullet}O_2^{-}$) was determined relative to Rose Bengal (Φ = 0.20). Two solutions containing either **Ir4a** or Rose Bengal (RB) were prepared, adjusting their absorbance to approximately 0.35 at 580 nm. DMPO was then added to a final concentration of 360 mM, and the solutions were transferred to capillary tubes as described above. The EPR signal was monitored over time as its intensity increased. For each compound, the second integral of the EPR signal was calculated at different time points and plotted as a function of time. The slope of the linear regression was determined and subsequently used in the following equation to calculate the superoxide quantum yield.

$$\Phi\ \text{comp}\ = \Phi\ ref\ \mathrm{X}\frac{\Delta comp}{\Delta ref}\mathrm{X}\frac{Abs\ ref}{Abs\ comp}$$

Determination of logP values.

The partition coefficients (log P) of the various Ir(III) complexes were determined using the shake-flask method, adapted from[4] n-Octanol-saturated Milli-Q water and water-saturated n-octanol were prepared in advance by vigorous mixing and phase separation.

For **Ir1b**, an aqueous solution (4 mL, 30 μM) was prepared by diluting a 10 mM DMSO stock into n-octanol-saturated Milli-Q water. The solution was sonicated and vortexed to ensure complete dissolution. A 2 mL aliquot was reserved as a reference, while the remaining 2 mL was mixed with

2 mL of water-saturated n-octanol. The biphasic mixture was shaken at 400 rpm for 1 h in an orbital mixer to allow equilibration, then centrifuged at 7800 rpm for 5 min to separate the phases. UV-Vis absorption spectra of the aqueous phase (after partitioning) and the reference aliquot were recorded using a Jasco V-550 spectrophotometer. The log P value was calculated as:

$$\log P = \log(K_{O/W}) = \log\left(\frac{A_0 - A}{A}\right)$$

where A_0 is the absorbance of the initial aqueous solution and A is the absorbance of the aqueous phase after partitioning, both measured at the compound's maximum absorption wavelength.

For the remaining compounds, a similar procedure was followed using n-octanol as the starting phase. A 4 mL solution (30 µM) was prepared by diluting the corresponding 10 mM DMSO stock into water-saturated n-octanol, followed by sonication and vortexing. After reserving a 2 mL aliquot as a reference, the remaining 2 mL was mixed with an equal volume of n-octanol-saturated Milli-Q water, shaken for 1 h at 400 rpm, and centrifuged. Absorption spectra of the organic phase (after partitioning) and the reference aliquot were acquired as above. Log P was calculated using:

$$\log P = \log(K_{O/W}) = \log\left(\frac{A}{A_0 - A}\right)$$

where A_0 is the absorbance of the initial octanol solution and A is the absorbance of the octanol phase after partitioning, both measured at the compound's maximum absorption wavelength. All measurements were performed in triplicate.

Nanoencapsulation of Ir4a in polyurethane-polyurea hybrid nanocapsules

Synthesis of Redox-Responsive Amphiphilic Cationic Prepolymer (P1)

2,2'-dihydroxyethyl disulfide (DEDS) (3.31 g, 21.44 mmol, 42.88 meq) and *N*-(3-dimethlaminopropyl)-*N,N'*-diisopropanolamine (Jeffcat DPA) (3.57 g, 16.35 mmol, 32.71 meq) were added into a 100 mL three-necked round-bottom flask equipped with a mechanical stirring at room temperature and purged with a N_2 stream. Subsequently YMER N120 (43.83 g, 43.36 mmol, 86.72 meq) was added hot to the mixture. When the mixture was homogeneous, isophorone diisocyanate (IPDI) (30.25 g, 136.11 mmol, 272.21 meq) was added into the reaction flask under light mechanical stirring. The polyaddition reaction was kept at 45 ºC under N_2 stream conditions until the NCO stretching band, monitored by IR spectroscopy, was stabilized. Once the NCO stretching band, did not change, the solution was cooled until room temperature. At this stage to fluidify the polymer, dry THF (32 mL) was added to the mixture. Simultaneously, 1,3-diamino-N-octadecylpropane (Genamin TAP 100D) (20.99 g, 63.98 mmol, 127.96 meq) was dissolved with dry THF (30 mL) in a 250 mL three-necked round-bottom flask purged with N_2 stream. The polymer mixture was added dropwise onto the Genamin TAP 100D solution under 80 rpm mechanical stirring immersed in a container of cold water. The reaction was monitored by IR spectroscopy until the completed disappearance of the NCO stretching band.

Synthesis of Ir-COUBPY-Loaded NCs (NC-Ir4a)

Ir4a (8.4 mg, 7.79 μmol), Neobee 1053 (23.3 mg, 46.20 μmol), polymer P1 (938.9 mg, 0.07 meq) and dry THF (1.6 mL) were mixed in a vial protected from light. At the same time, IPDI (27.9 mg, 0.13 mmol, 0.25 meq) was added into a 100 mL three-necked round-bottom flask equipped with mechanical stirring at room temperature, purged with a N_2 stream and protected from light. The mixture was added into the flask and homogeneized for 5 min at 80 rpm protected from light. At this point, an alkaline aqueous solution of L-Lysine previously prepared at total L-Lysine concentration 7,56% by weight at adjusted pH to 11.5 with 3M and 1M NaOH solutions (10.6 mg of L-Lysine, 0.06 mmol, 0.12 meq) was added at 250 rpm. The polyaddition reaction was monitored after 5 min by IR spectroscopy. Subsequently, the organic phase was emulsified at 300 rpm with cold Mili-Q water (8.0 g). Finally, a 10% w/w aqueous solution of diethylentriamine (DETA) (2.0 mg of DETA, 0.02 mmol, 0.06 meq) was added to generate crosslinked NCs and monitored by IR. Once the NCs were formed, the THF was removed from the reactor under N_2 stream and purified by dialysis purification.

The NCs were characterized by different analytical techniques. Firstly, the synthetic process was monitored by Infrared Spectroscopy (FT-IR) and pH measurements to confirm that the final polyamine crosslink the remaining reactive sites along the nanoparticle shell. Afterwards, **NC-Ir4a** were purified by dialysis against Milli-Q water for 38 h, and characterized by DLS and TEM.

To determine the amount of Ir-COUBPY complex incorporated in the NCs, iridium was quantified by ICP-MS according to the following procedure. First, a fixed volume of NC emulsion (previously dialyzed) was diluted in 500 µL of concentrated 72% (v/v) nitric acid into Wheaton v-vials (Sigma-Aldrich) and heated in an oven at 373 K for 18 h. The vials were then allowed to cool, and each sample solution was transferred into a volumetric tube and combined with Milli-Q water washings (1.5 mL). Digested samples were diluted 4 times with Milli-Q to obtain a final HNO_3 concentration of approximately 18% (v/v). Iridium content was analyzed on a Nexion350D PerkinElmer instrument at the Centres Científics i Tecnològics of the Universitat de Barcelona. The solvent used for all ICP-MS experiments was 1% HNO_3-containing Milli-Q water. Iridium standards was freshly prepared in Milli-Q water with 1% HNO_3 before each experiment. The concentrations used for the calibration curve were, in all cases, 0, 0.2, 0.4, 1, and 2 ppb. Isotopes detected was ^{193}Ir. Readings were performed in triplicate for each sample. Rhodium was added as an internal standard at a concentration of 10 ppb in all samples. The loading was 326.58 ± 0.06 μM.

Dynamic light scattering (DLS).

DLS measurements were performed using a BeNano 90 Zeta (Nane Equipamientos S.L.). The Ir(III)-loaded nanocapsules were diluted in ultrapure water (Mili-Q) to a final concentration of 0.05% (w/v) and homogenized 1 min with vortex. The measurements were carried out in disponsable polystyrol/polystyrene cuvettes, and each sample was measured in triplicate to ensure reproducibility.

Transmission electron microscopy (TEM).

TEM analysis was performed using a microscope model JEOL JEM1010 operating at an

accelerating voltage of 80 kV. Samples were prepared by placing on the top of a drop (10 mL) of the nanoparticle dispersion diluted at 0.4 % (w/v) a Formvar/carbon-coated copper grid (200 mesh, Electron Microscopy Sciences). After 30 min, the excess was gently removed using filter paper. After, the samples were negatively stained with 2% (w/v) uranyl acetate for 30 seconds, followed by an excess removal and drying for 2 4h minimum. Images were captured using a camera, and the nanoparticle size and morphology studied were analyzed using ImageJ software.

Cell Culture

A375 and HeLa cells were obtained from a certified cell bank and maintained under appropriate culture conditions. The cells were cultured in a humidified incubator at 37°C with 5% CO_2. HeLa cells were grown in Dulbecco's Modified Eagle's Medium (DMEM) containing 1 g/L glucose and 2 mM glutamine, while A375 cells were maintained in DMEM supplemented with 4.5 g/L glucose and 4 mM glutamine. Both media were further enriched with 10% fetal bovine serum (FBS) and 1% penicillin-streptomycin. Cells were routinely subcultured upon reaching 70–80% confluency using trypsin-EDTA solution. To ensure experimental validity, all cell lines were periodically tested for mycoplasma contamination.

Cellular uptake by confocal microscopy

Cell Culture and Treatments. HeLa cells were maintained DMEM (1966021 Gibco) containing GlutaMAX and high glucose (4.5 g/L) supplemented with 10% (FBS) and 50 U/mL penicillin-streptomycin. For cellular uptake experiments and posterior observation under the microscope, cells were seeded on glass-bottom dishes (P35G-1.5-14-C, Mattek). 24 h after cell seeding, cells were incubated for 30 min at 37 °C with the compounds (5 or 20 µM) in supplemented DMEM. Then cells were washed two times with Dulbecco's Phosphate-Buffered Saline (DPBS, pH 7.0-7.3) to remove the excess of the compounds and kept in low glucose DMEM with HEPES (10mM, Corning) and without phenol red for fluorescence imaging.

For colocalization experiments with Mitoview 650, HeLa cells were treated with the compounds (5 or 20 µM) for 30 min at 37 °C. Then cells were washed with DPBS and incubated with Mitoview 650 (0.1 µM) for 30 min at 37 °C in non-supplemented DMEM. After removal of the medium and washing two times with DPBS, cells were kept in low glucose DMEM with HEPES (10mM, Corning) and without phenol red for fluorescence imaging.

Fluorescence Imaging. All microscopy observations were performed using a Zeiss LSM 880 confocal microscope equipped with a 405-nm diode laser, an argon-ion laser for excitation at 488, and 514 nm, and solid-state lasers for excitation at 561 nm and 633 nm. The microscope was also equipped with a Heating Insert P S (Pecon). Cells were observed at 37ºC using a 63× 1.4 oil immersion objective. All Ir-bpy complexes were excited at 405 nm, with emission detected between 500–625 nm. For Ir-COUBPY complexes, **Ir1a** and **Ir3a** were excited at 561 nm with emission detected between 570–640 nm, while free complex **Ir4a** and nanoformulations **NC-Ir4a** were excited at 514 nm with emission detected between 520–640 nm. Mitoview 650 was excited at 633 nm, with emission detected between 670–750 nm. In all observations a stack of images

was acquired with a voxel size of 0.13 x 0.13 x 0.5 µm (xyz). Image processing and analysis were performed using Fiji.[5]

Image analysis. The Mitoview and the compound channels were processed by median filtering (radius = 1), Gaussian filtering (sigma = 1), and background subtraction (rolling ball radius = 30). In all experiments colocalization coefficients were measured in 3D using the JaCoP plugin on the stacks of images acquired. For each compound more than 90 cells on average were analysed.

Cellular uptake by ICP-MS

A375 cells were seeded in 12-well plates at a density of 4 × 10^5 cells per well and incubated for 24 h prior to treatment. Then, the cells were treated with 10 µM of the Ir complexes and cisplatin (control) for 1 h at 37°C. After trypsinization, the A375 cells were collected, counted and subjected to digestion in 30% HNO_3 at room temperature overnight. The intracellular iridium content was then quantified using inductively coupled plasma mass spectrometry (ICP-MS). The experiment was conducted in three independent experiments (n = 2).

Assessment of light-induced cytotoxicity

To evaluate the photocytotoxicity of the Ir-COUBPY and Ir-bpy complexes, A375 and HeLa cells were seeded into 96-well plates at a density of 5 × 10^3 cells per well in DMEM supplemented with 10% FBS and 1% penicillin-streptomycin. The plates were incubated at 37°C in a humidified 5% CO_2 atmosphere for 24 h before treatment. Stock solutions of the tested iridium complexes were prepared in DMSO and diluted in DMEM to final concentrations ranging from 0 to 100 µM. The final DMSO concentration was kept below 0.4% to minimize solvent-induced cytotoxicity. Following medium replacement, cells were incubated for 1 h in the dark with the compounds' solutions. After incubation, one set of plates remained in darkness, while another was subjected to green (λ = 520 nm) or red (λ = 620 nm) light irradiation using an LED-based illumination system at a power density of 1.5 and 15 mW/cm^2, respectively, for 1 h. The plates were then returned to standard culture conditions for a drug-free recovery period of 48 h. Cell viability was assessed using the MTT assay. Briefly, after the recovery period, 50 µL of MTT reagent (1 mg/mL) was added to each well and plates were incubated at 37°C for 2 h. Formazan crystals were subsequently dissolved in 50 µL of DMSO, and absorbance was measured at 570 nm using a FLUOstar Omega microplate reader. The IC_{50} values under dark and light conditions were determined from dose-response curves using GraphPad Prism 10 with non-linear regression analysis. The phototoxicity index (PI) was obtained by calculating the ratio of IC_{50} in the dark to IC_{50} under irradiation, providing a quantitative measure of light-induced cytotoxicity. Each experimental condition was conducted in three independent experiments with triplicate wells per concentration (n = 3) to ensure statistical reliability and reproducibility.

Photo-induced ROS generation

A375 cells were seeded in 12-well plate at the density of 3 x 10^5 cells/well and incubated overnight. Cells were then treated with 250 nM of the **Ir4a** and **NC-Ir4a**. TPA (20 µM) and NaPyr (10 mM) were used to scavenge •OH and H_2O_2, respectively. Treatments were followed with 1 h

incubation in the dark and then 1 h irradiation with green light. After irradiation, the media was removed and cells were trypsinized, collected and stained with 2′,7′-dichlorofluorescein diacetate (DCFH-DA, 10 μM) for 30 min in the dark. Flow cytometry was then performed using a blue laser (488 nm) for excitation and detection at 530 nm. The assay was conducted in at least two independent experiments (n = 2 per replicate).

DNA damage assessment

DNA damage was evaluated in A375 cells using flow cytometry. Briefly, cells were seeded in 12-well plates at a density of 3 × 10^5 cells/well and incubated under appropriate growth conditions for 24 h. Cells were then treated with 250 nM **Ir4a** or **NC-Ir4a**, followed by green light irradiation. Cisplatin (10 μM) was used as a positive control for DNA damage induction. After treatment, cells were trypsinized, washed with PBS, and fixed in 200 μL of 0.2% paraformaldehyde (PFA) for 5 min. Following fixation, cells were pelleted and resuspended in a 3% FBS solution containing an anti-phospho-H2AX (Ser139) FITC-conjugated monoclonal antibody (CR55T33, eBioscience™) at 0.6 μg/mL, then incubated for 2 h at room temperature in the dark. Stained cells were analyzed using a Becton Dickinson FACSCalibur flow cytometer (λ exc = 488 nm). The experiment was conducted in two independent replicates (n = 2 per experiment).

Microscopy imaging analyses

Widefield Fluorescence Microscopy. Fluorescence microscopy experiments were conducted using a Thunder Leica DMi8 inverted microscope. A375 cells were cultured on ibidi μ-slide plates for 2-3 days and treated with the selected Ir(III) complexes under light irradiation, stained with DHE and captured using a DFC 9000 sCMOS camera (Leica, Weztlar).

Field emission scanning electron microscopy. A375 cells were treated with the selected Ir(III) complexes and cisplatin. Cells then were fixed with 4% glutaraldehyde fix solution for 30 min and washed with PBS. Sample were dehydrated through a graded series of ethanol (30, 50, 70, 95 and 100%) and critical point drying was performed.. FESEM samples were mounted on aluminium stubs and platinum sputter-coated with 5.0 nm thin layer (Leica EM ACE 600). FESEM analyses were performed with a FE-SEM (ApreoS Lovac IML Thermofisher. Sample analyses were performed at 20 kV.

Mitochondrial membrane potential assay

A375 cells were seeded in a 12-well plate at a density of 2 × 10^6 cells per well and treated with **Ir4a**, **NC-Ir4a** (photoirradiated, 250 nm) or Antimycin A (as a positive control) for 6 h. Following the treatment, the cells were harvested and resuspended in prewarmed PBS containing JC-1 dye (1 μM). The cell suspension was then incubated at 37°C for 30 min. After incubation, the cells were washed twice with PBS and immediately analyzed using a FACSCAlibur flow cytometer (Becton Dickinson). Fluorescence was measured by detecting both the monomeric form of JC-1 (green fluorescence, emission at 525 ± 30 nm) and the aggregated form (red fluorescence, emission at 585 ± 30 nm) upon excitation at 488 nm. The collected data were analyzed using FlowJo™ v10 software.

Cell metabolism measurements

Mitochondrial oxidative phosphorylation (OXPHOS) in A375 cells was assessed by measuring the oxygen consumption rate (OCR) using a Seahorse XFe96 extracellular flux analyzer. Briefly, A375 cells were seeded at a density of 2 × 10^4 cells per well in XFe96-well culture microplates (Seahorse Agilent) one day prior to the experiment. The sensor cartridge was hydrated overnight by immersing it in calibration buffer at 37°C in a non-CO_2 incubator. For the assay, buffered DMEM (Seahorse Bioscience) was used. Cells were treated with **Ir4a**, **NC-Ir4a**, or cisplatin for comparison. Cellular metabolism was analyzed using the XF Glycolytic Rate Test Kit. OCR and extracellular acidification rate (ECAR) were monitored in real time, and respiration rates were averaged before and after the injection of a mixture of complex III electron transport chain inhibitors (Rotenone/Antimycin A, 1 μM) to disrupt OXPHOS and a glycolysis inhibitor (2-deoxyglucose, 50 mM) to block glucose metabolism. All experiments were performed in triplicate.

ATP determination

Intracellular ATP levels were measured using a recombinant firefly luciferase bioluminescence assay following the manufacturer's instructions (Invitrogen™). Briefly, A375 cells (2 × 10^5 cells per well) were seeded in a 12-well plate and incubated for 24 h in complete medium. The cells were then treated with varying concentrations of **Ir4a**, **NC-Ir4a**, and cisplatin for 6 h. After treatment, the cells were trypsinized, and the resulting pellets were lysed using chilled cell lysis buffer (eBioscience™). The intracellular extracts were then combined with a buffered reaction solution containing D-luciferin and luciferase as per the protocol. Luminescence was measured at 560 nm in white 96-well plates using a FLUOstar Omega microplate reader. All experiments were conducted in triplicate (n = 3).

3D Multicellular tumor spheroid (MCTS) viability assay

A375 multicellular tumor spheroids (MCTS) were treated with **Ir4a** or **NC-Ir4a** at a concentration of 2.5 μM for 1 h, followed by 1 h of green light irradiation. Cisplatin (25 μM, dark conditions) was used as a positive control. After irradiation, the treatment solutions were replaced with fresh culture medium, and the spheroids were incubated in the dark. The treatment cycle was repeated after two days. Following a 24-h recovery period, spheroids were stained with calcein-AM (2 μM) and propidium iodide (2 μg/mL) for 30 min at 37 °C in a 5% CO_2 atmosphere. Fluorescence images were acquired using a Zeiss Axio Observer 7 inverted fluorescence microscope.

2. Synthetic procedures

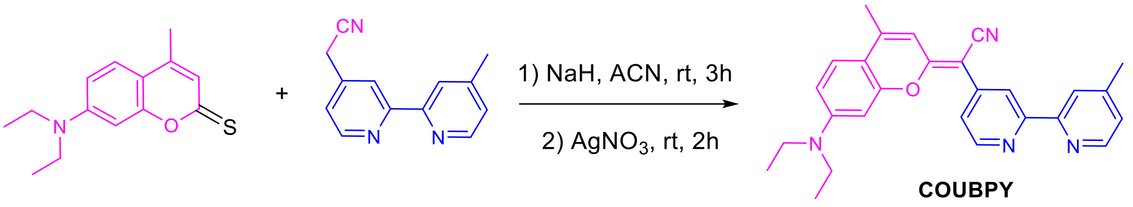

Scheme S1. Synthetic route for the preparation of **COUBPY** ligand.

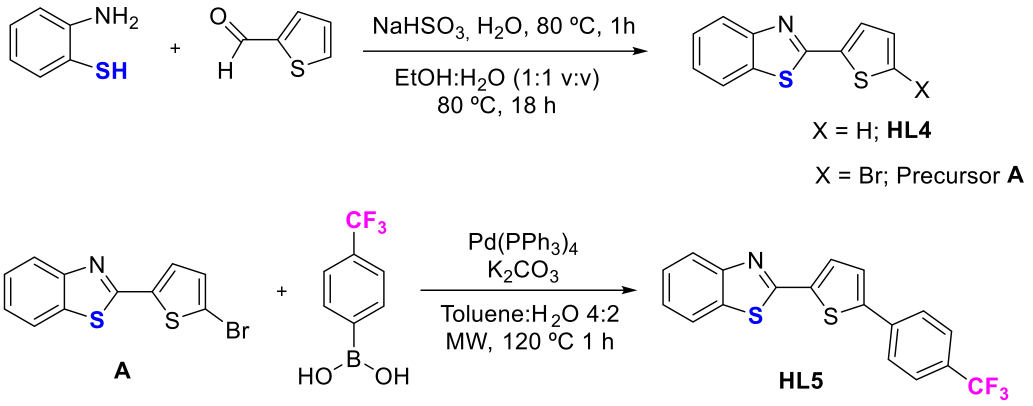

Scheme S2. Synthetic procedure for **HL4** and **HL5.**[1]

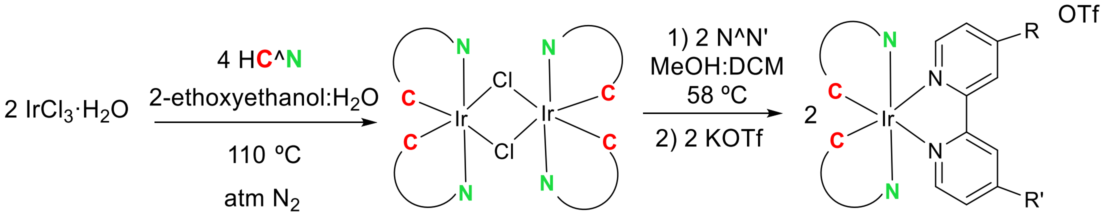

Ir1a-Ir5a; N^N = COUBPY; R = Me, R' = coumarin

Ir1b-Ir5b; N^N = bpy; R = R' = H

Scheme S3. Synthetic procedure for Ir-COUBPY (**Ir1a–Ir5a**) and Ir-bpy (**Ir1b–Ir5b**) complexes.

3. NMR spectroscopy

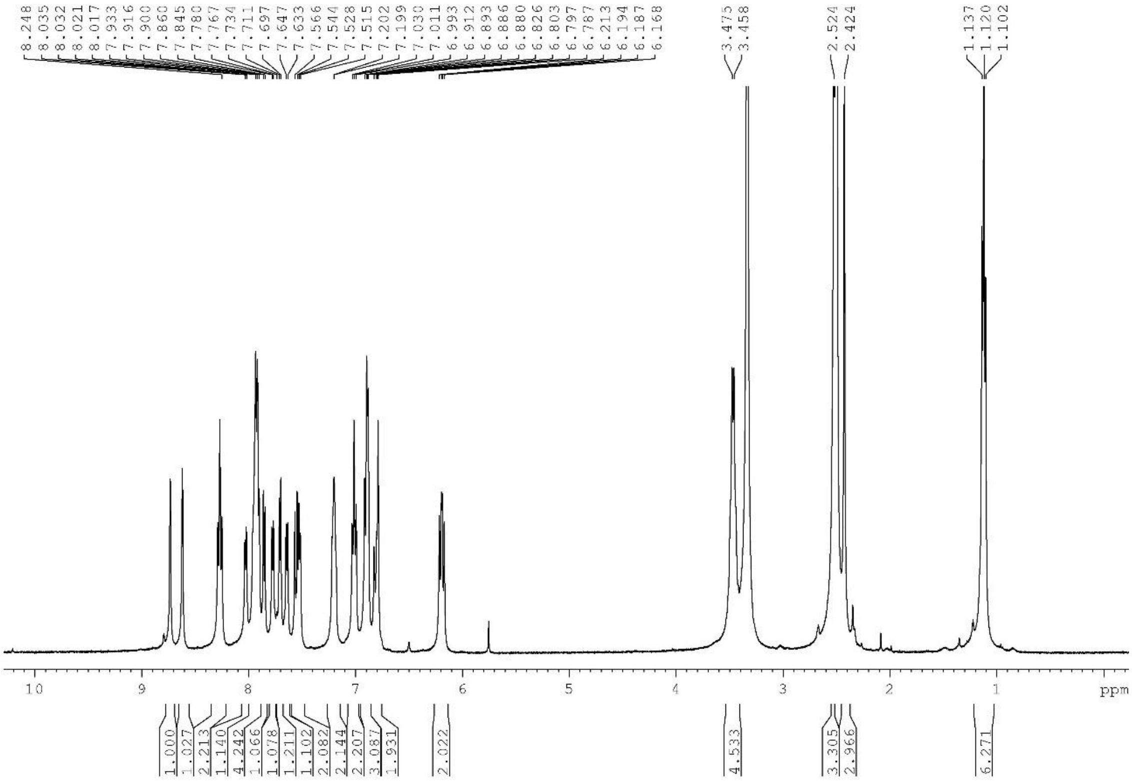

Figure S1. ^{1}H NMR spectrum of **Ir1a** in DMSO-d_6.

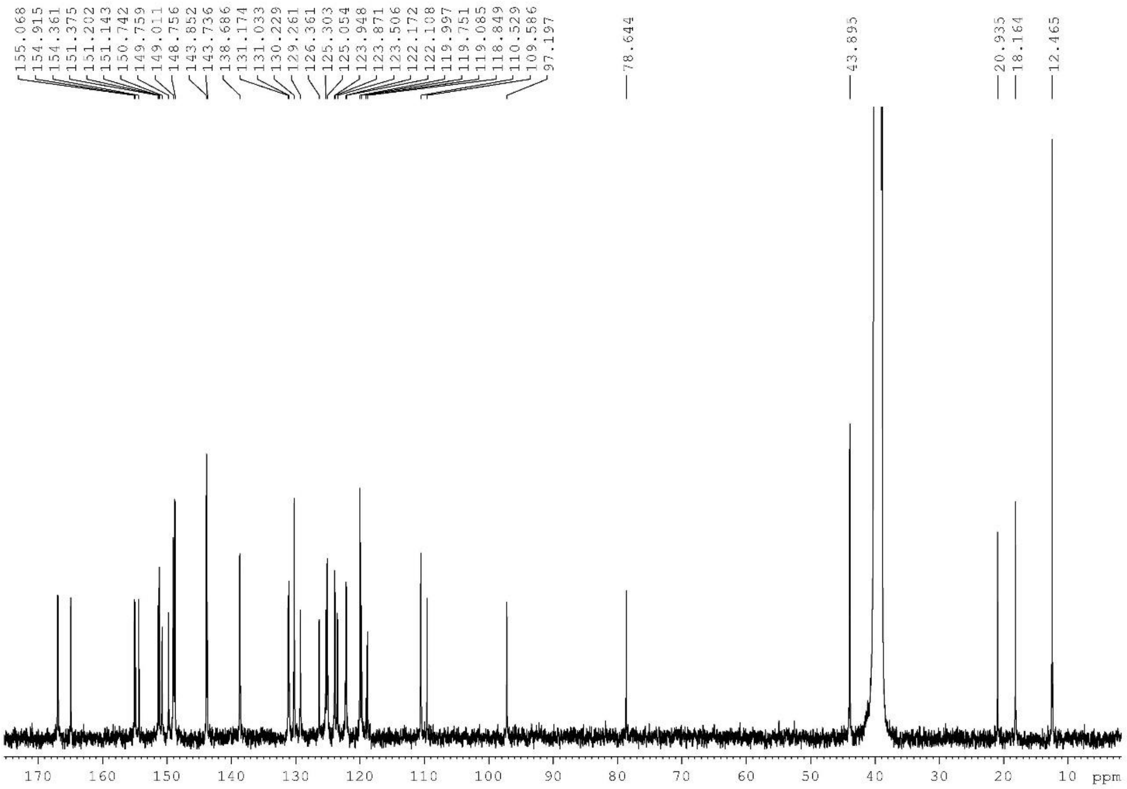

Figure S2. ^{13}C NMR spectrum of **Ir1a** in DMSO-d_6.

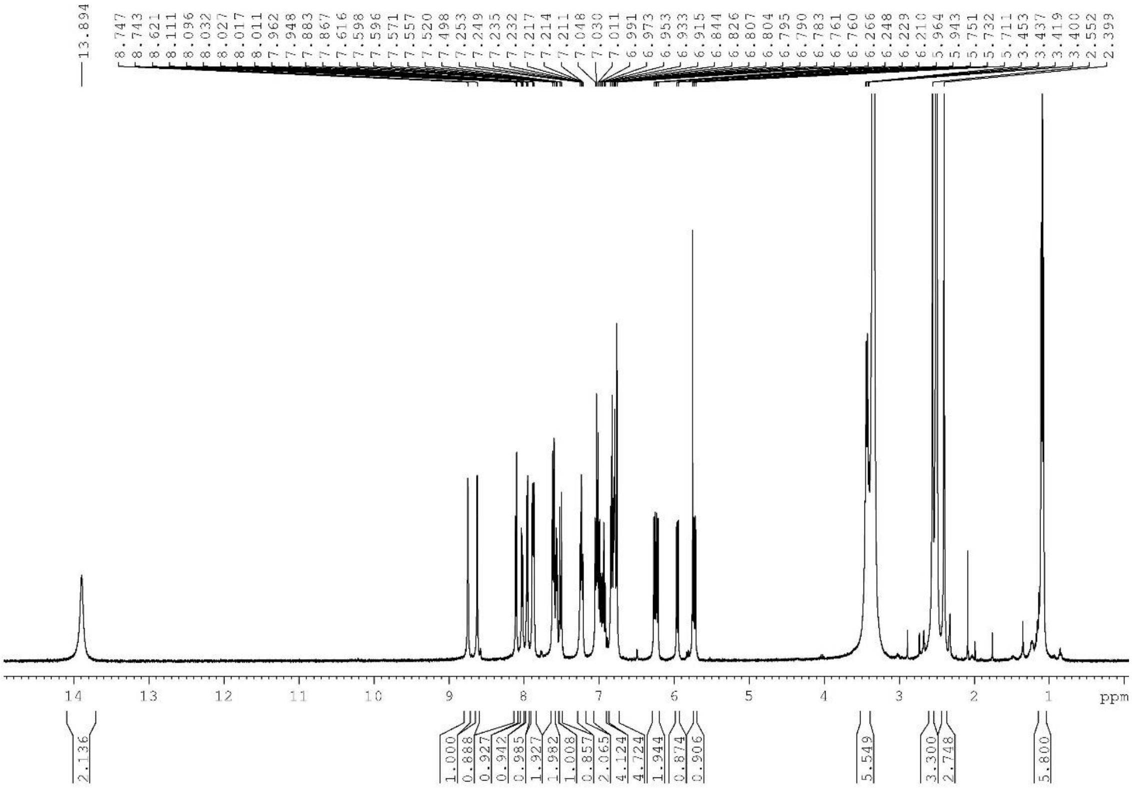

Figure S3. ^{1}H NMR spectrum of **Ir2a** in DMSO-*d*$_6$.

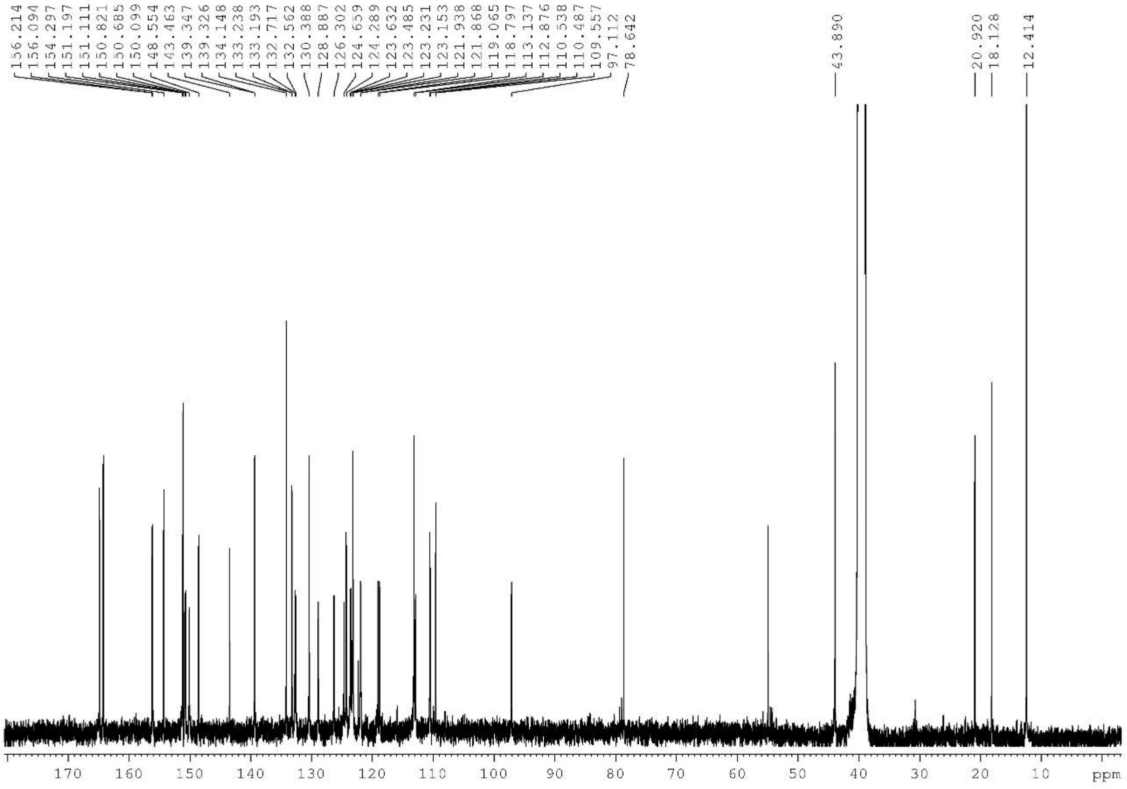

Figure S4. ^{13}C NMR spectrum of **Ir2a** in DMSO-*d*$_6$.

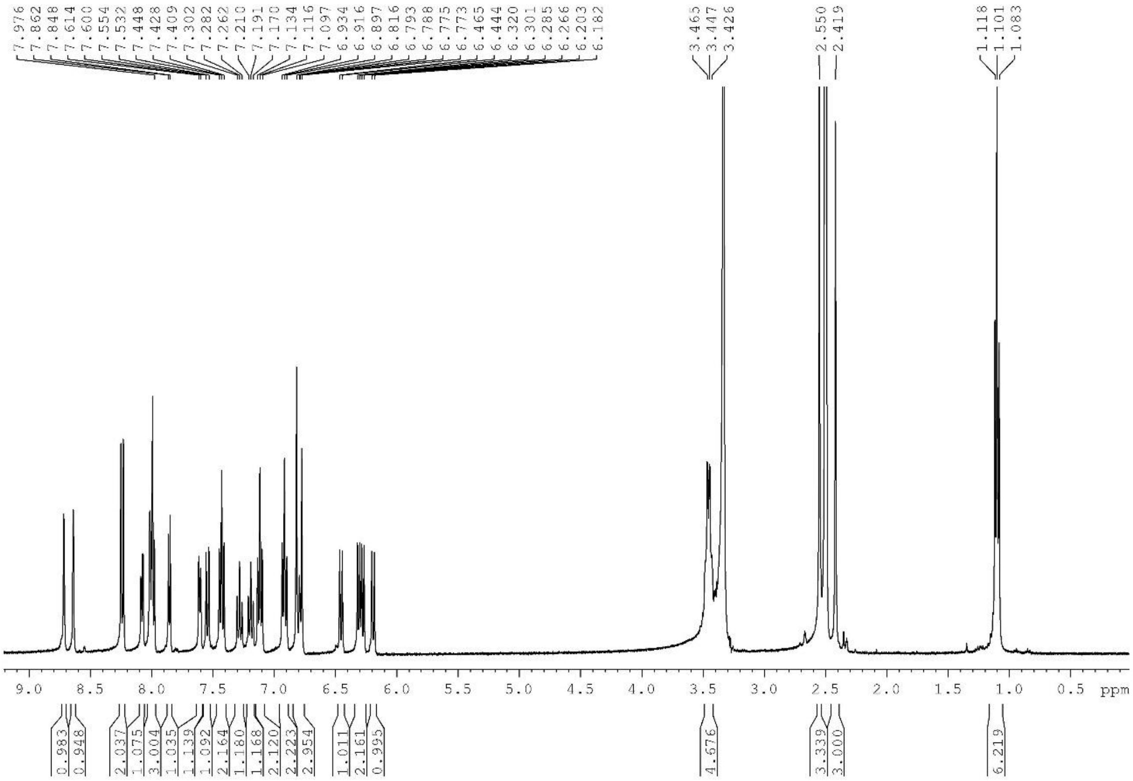

Figure S5. ^{1}H NMR spectrum of **Ir3a** in DMSO-d_6.

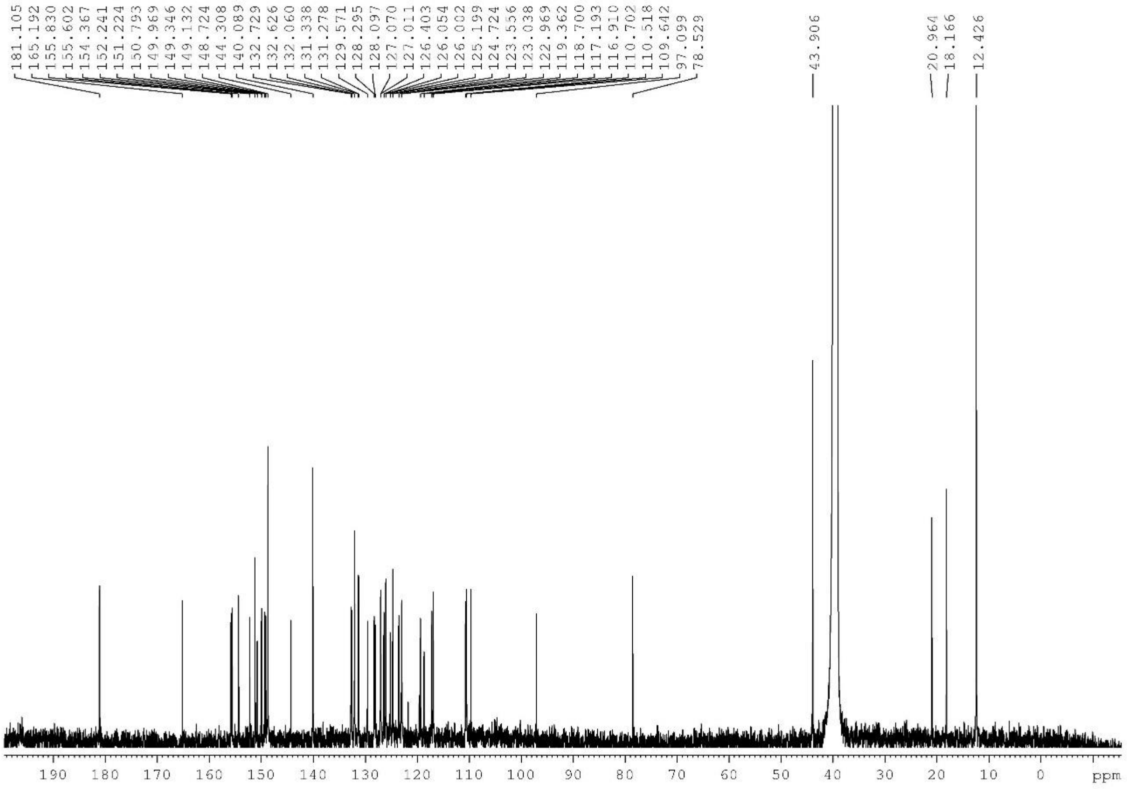

Figure S6. ^{13}C NMR spectrum of **Ir3a** in DMSO-d_6.

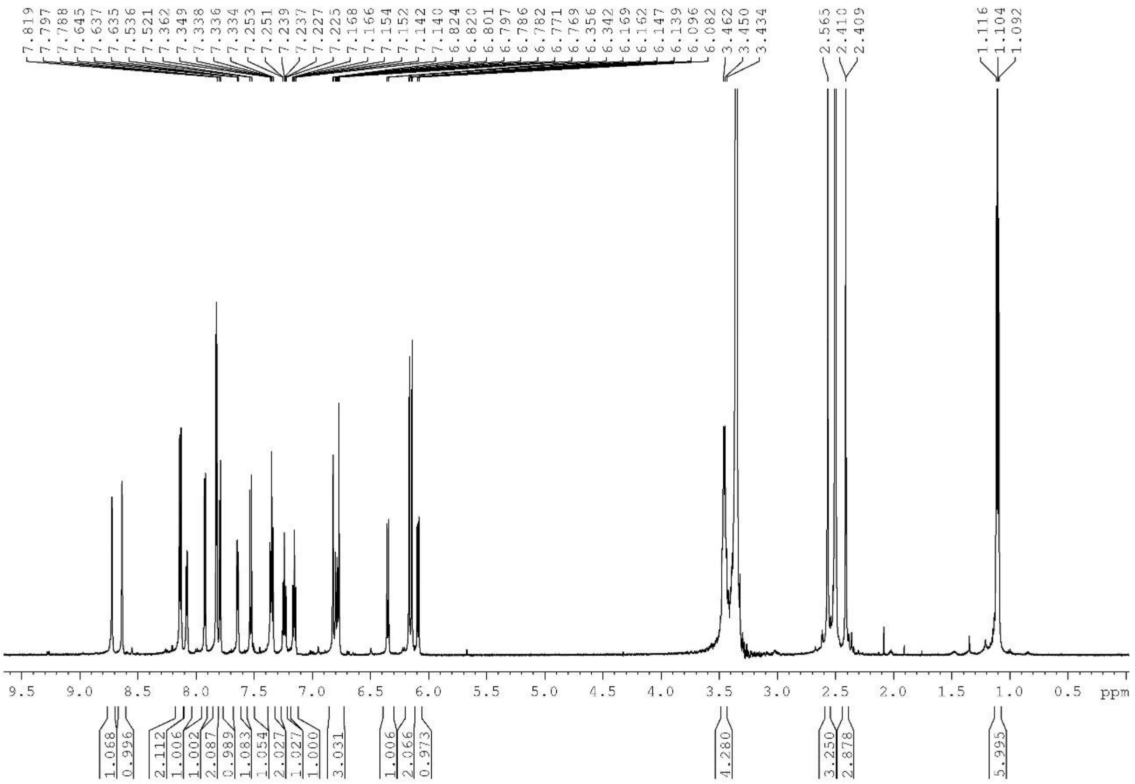

Figure S7. ^{1}H NMR spectrum of **Ir4a** in DMSO-d_6.

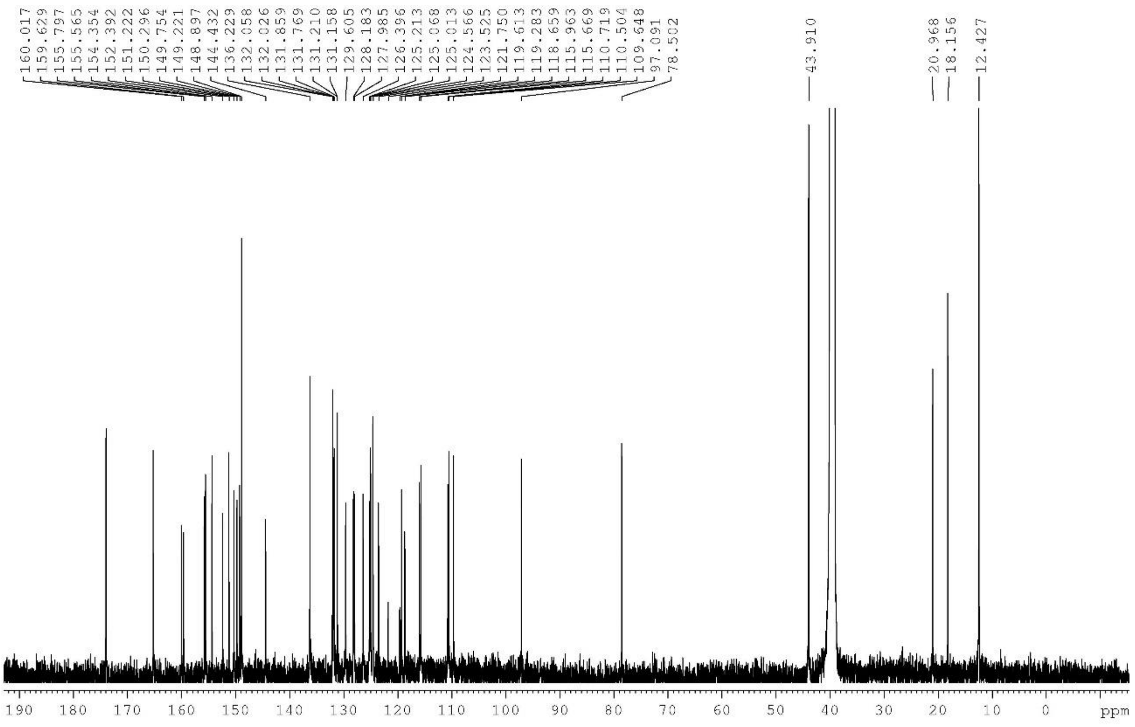

Figure S8. ^{13}C NMR spectrum of **Ir4a** in DMSO-d_6.

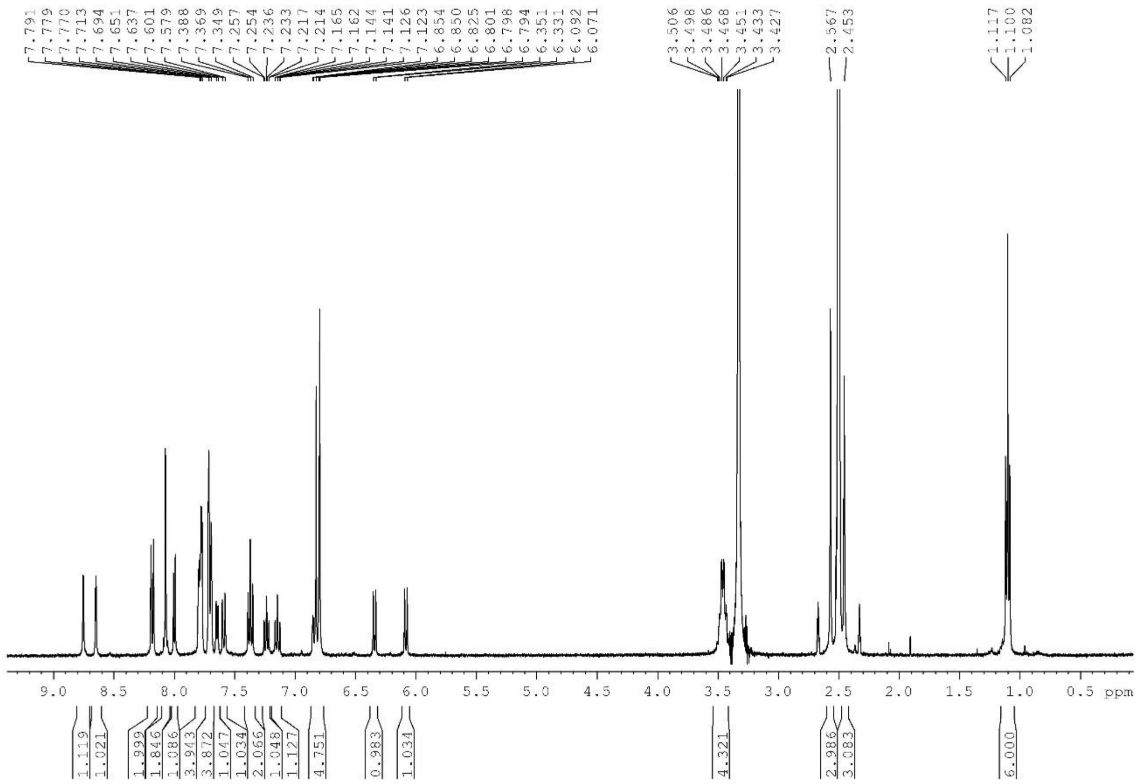

Figure S9. ^{1}H NMR spectrum of **Ir5a** in DMSO-d_6.

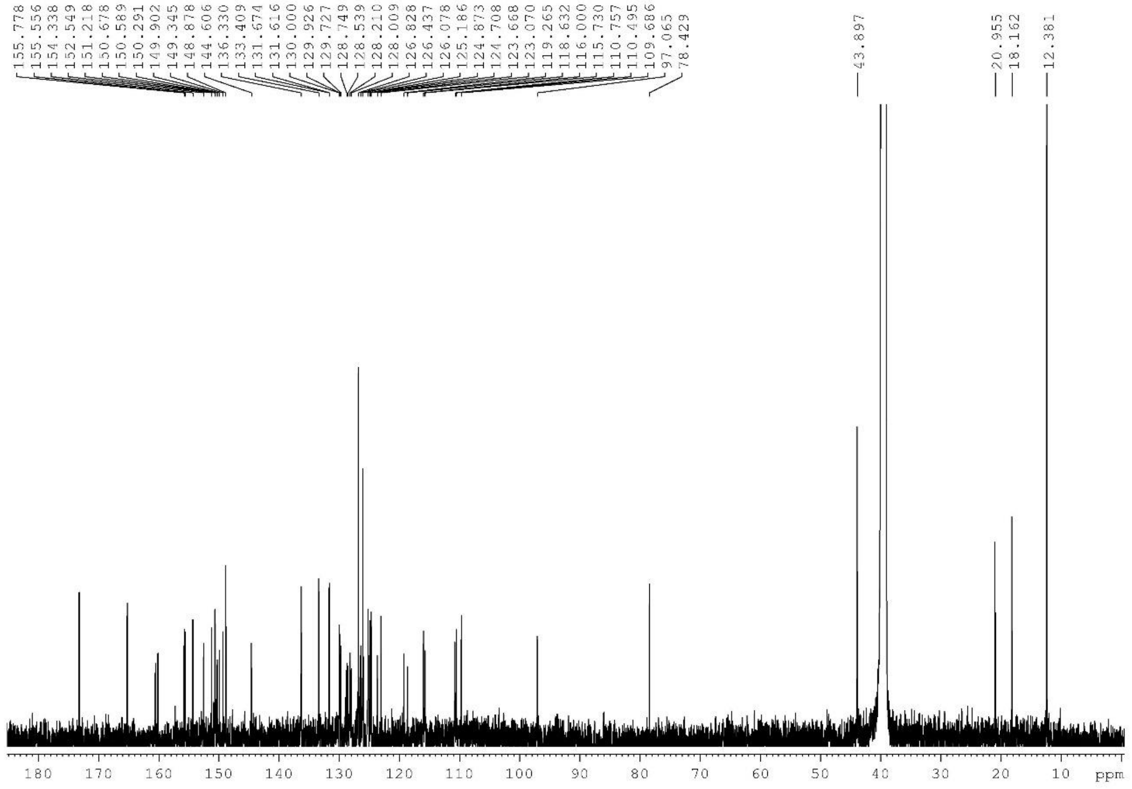

Figure S10. ^{13}C NMR spectrum of **Ir5a** in DMSO-d_6.

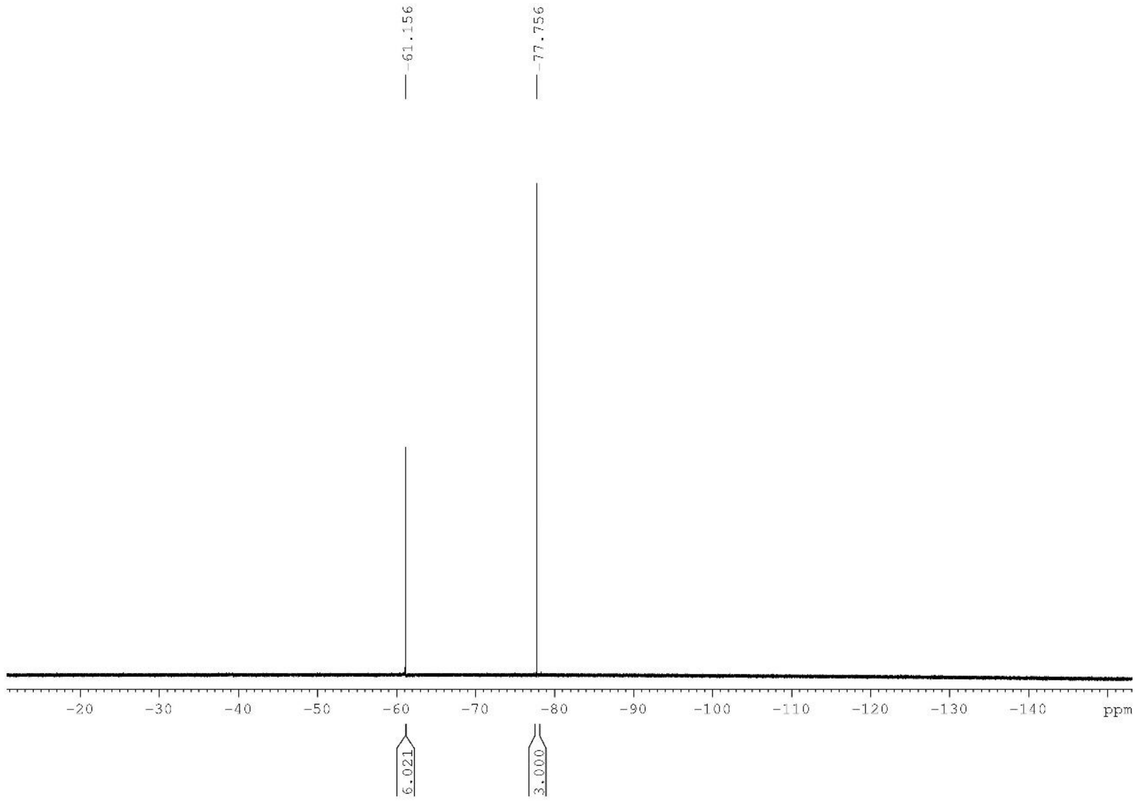

Figure S11.^{19}F-NMR spectrum of **Ir5a** complex in DMSO-d_6.

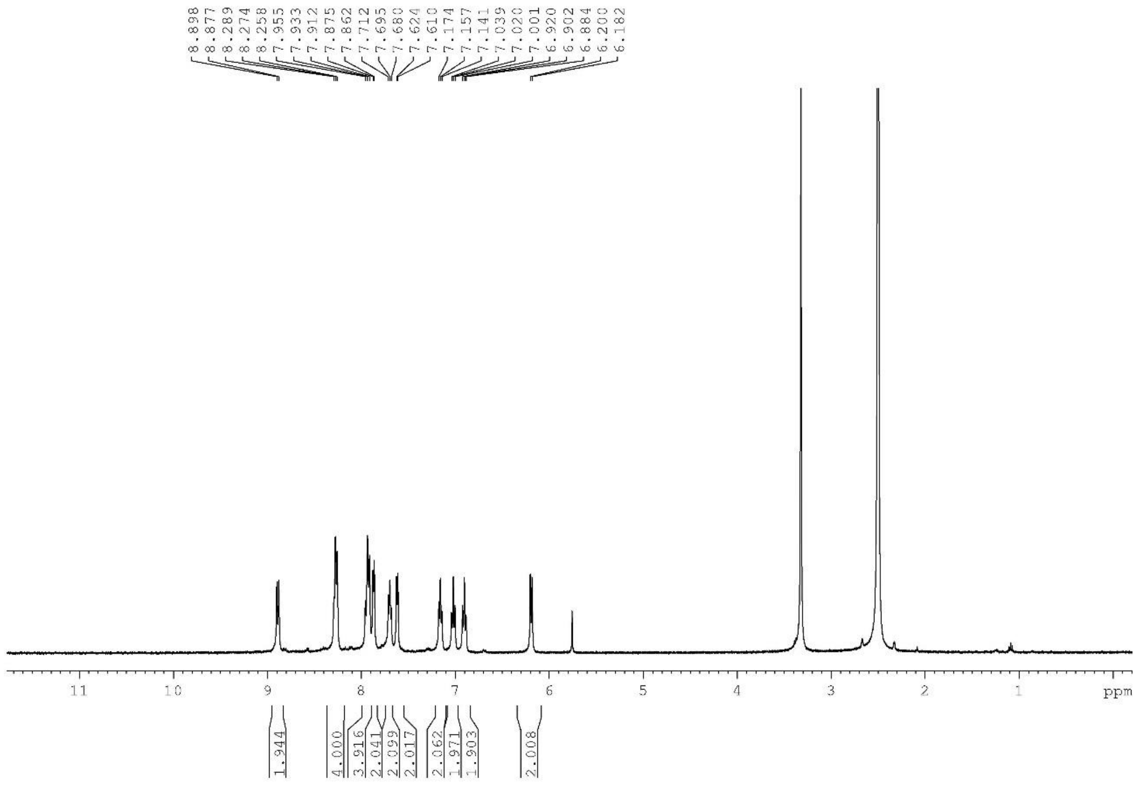

Figure S12. ^{1}H NMR spectrum of **Ir1b** in DMSO-d_6.

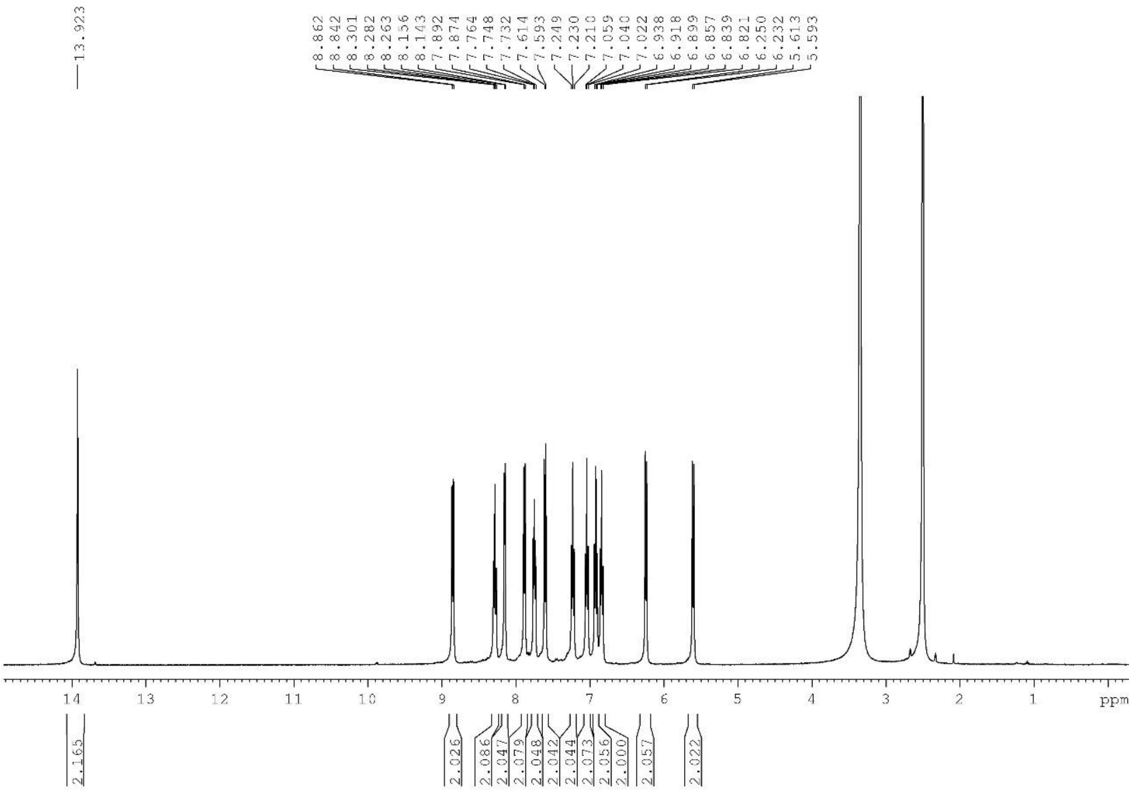

Figure S13. ^{1}H NMR spectrum of **Ir2b** in DMSO-d_6.

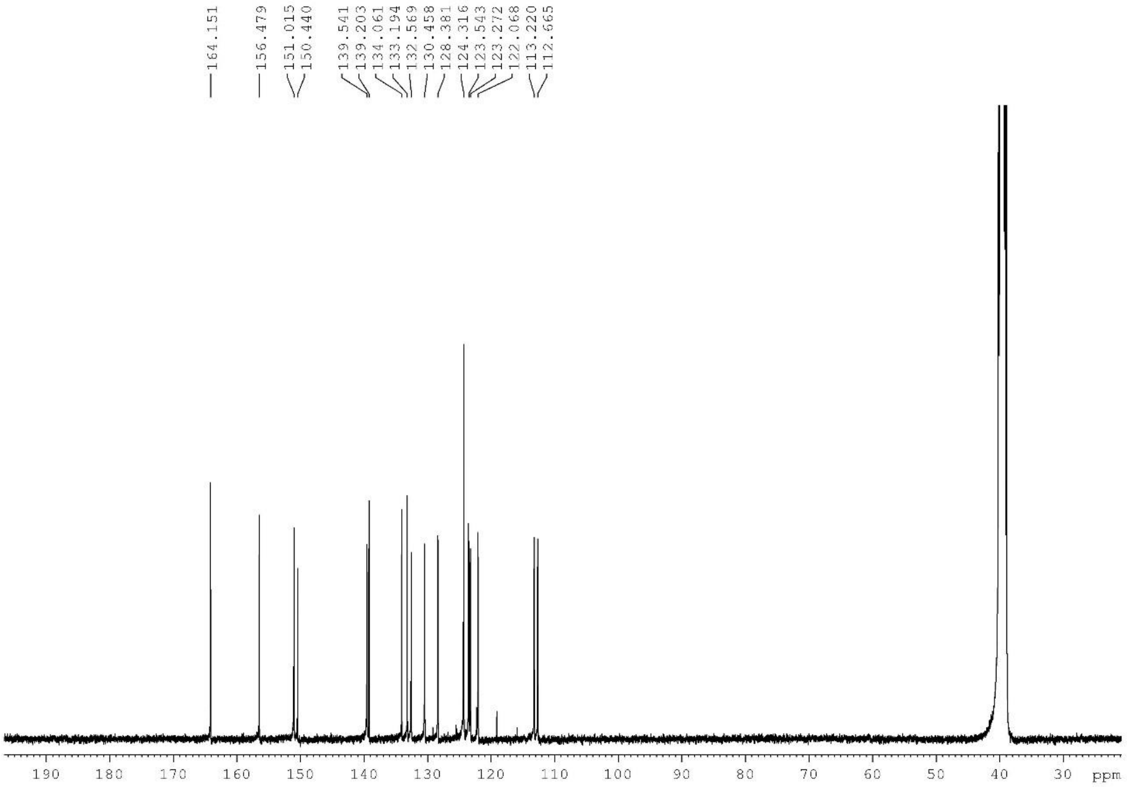

Figure S14. ^{13}C NMR spectrum of **Ir2b** in

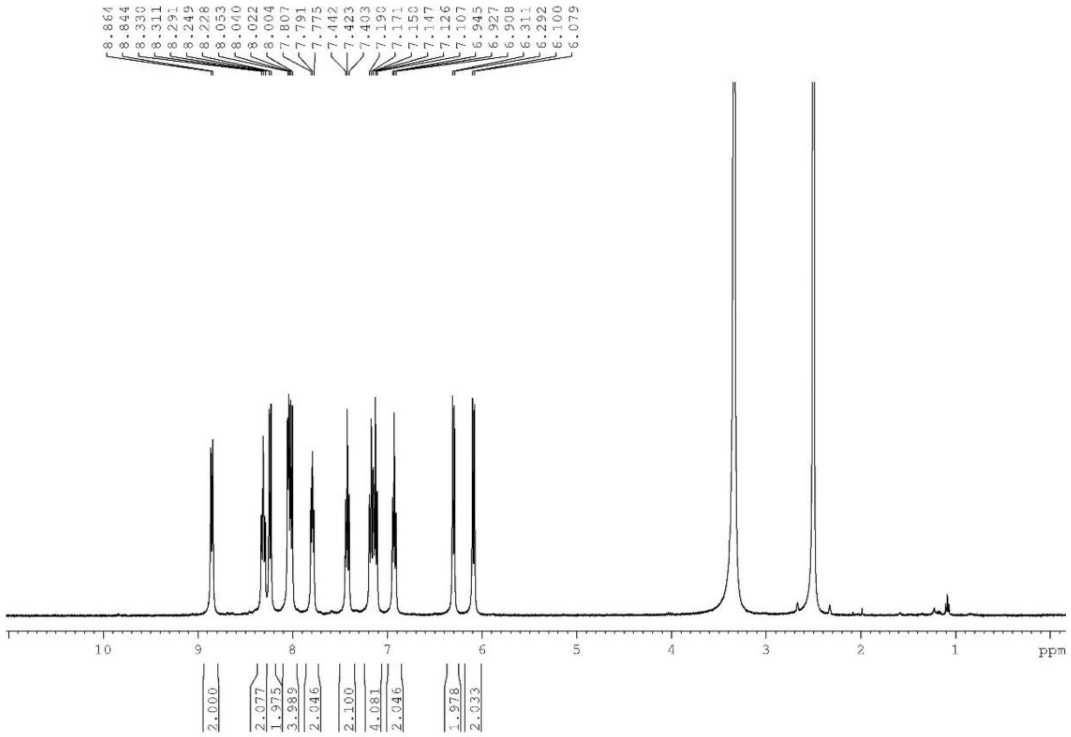

Figure S15. ^{1}H NMR spectrum of **Ir3b** in DMSO-*d*$_6$.

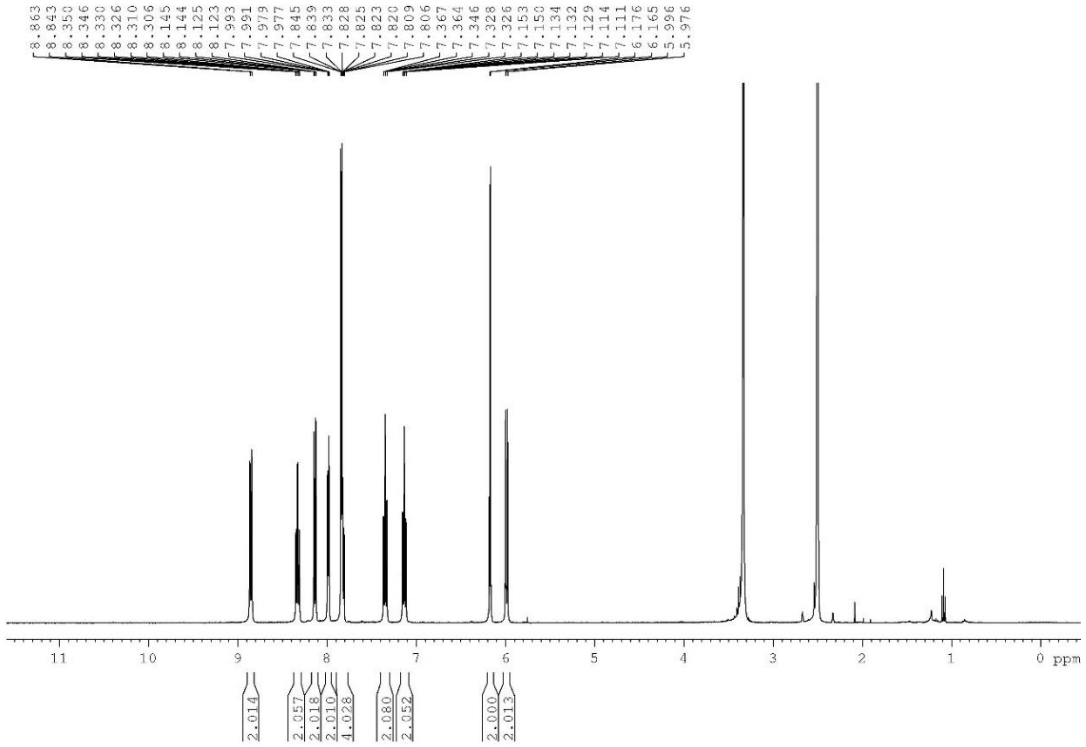

Figure S16. ^{1}H NMR spectrum of **Ir4b** in DMSO-*d*$_6$.

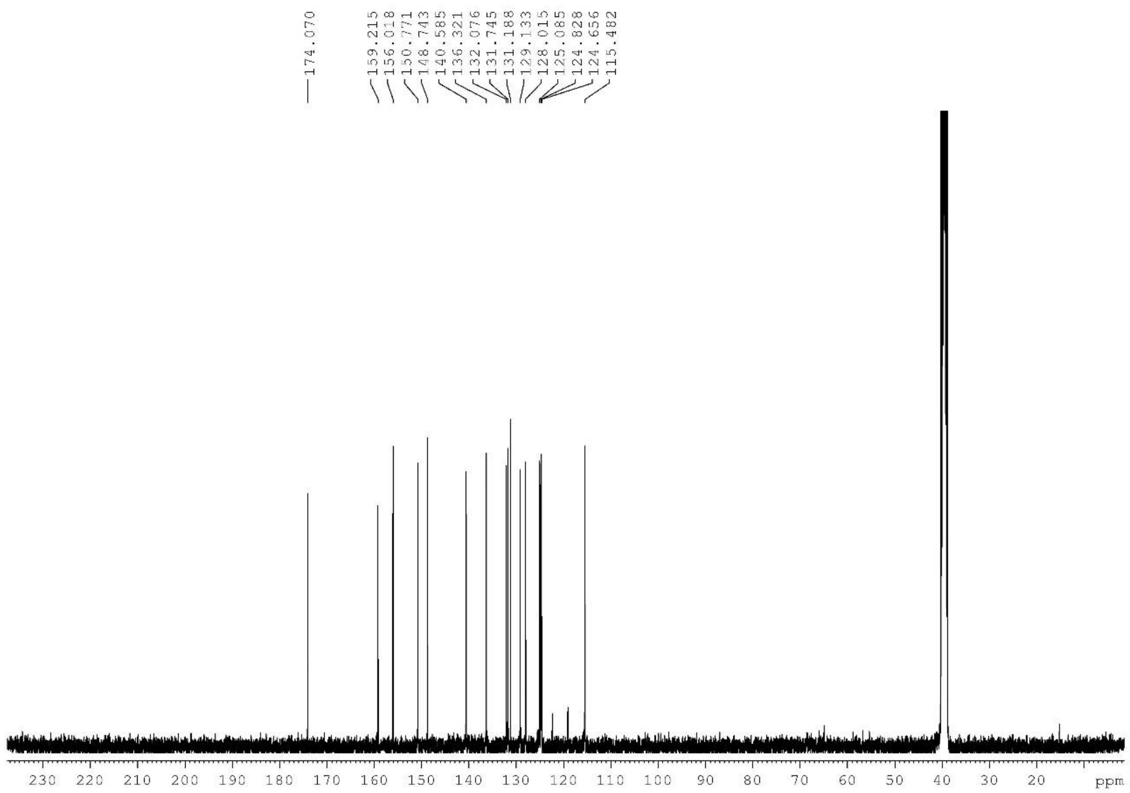

Figure S17. ^{13}C NMR spectrum of **Ir4b** in DMSO-d_6.

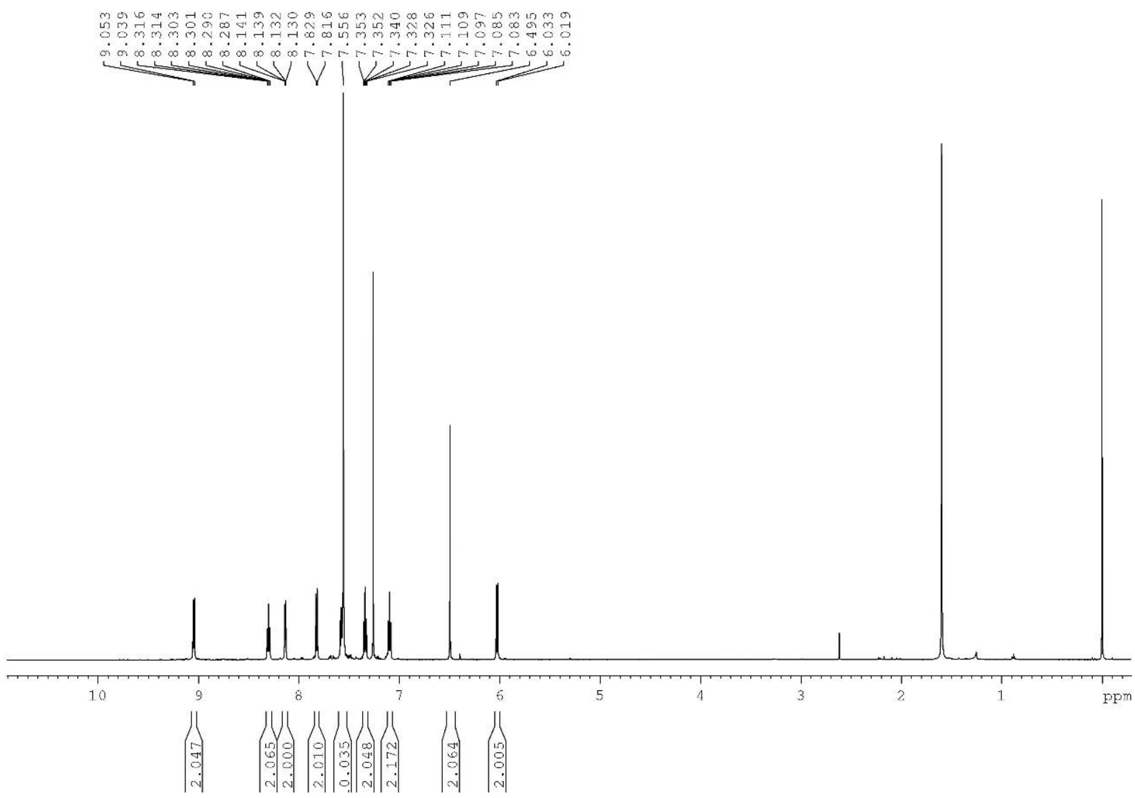

Figure S18. ^{1}H NMR spectrum of **Ir5b** in CDCl$_3$.

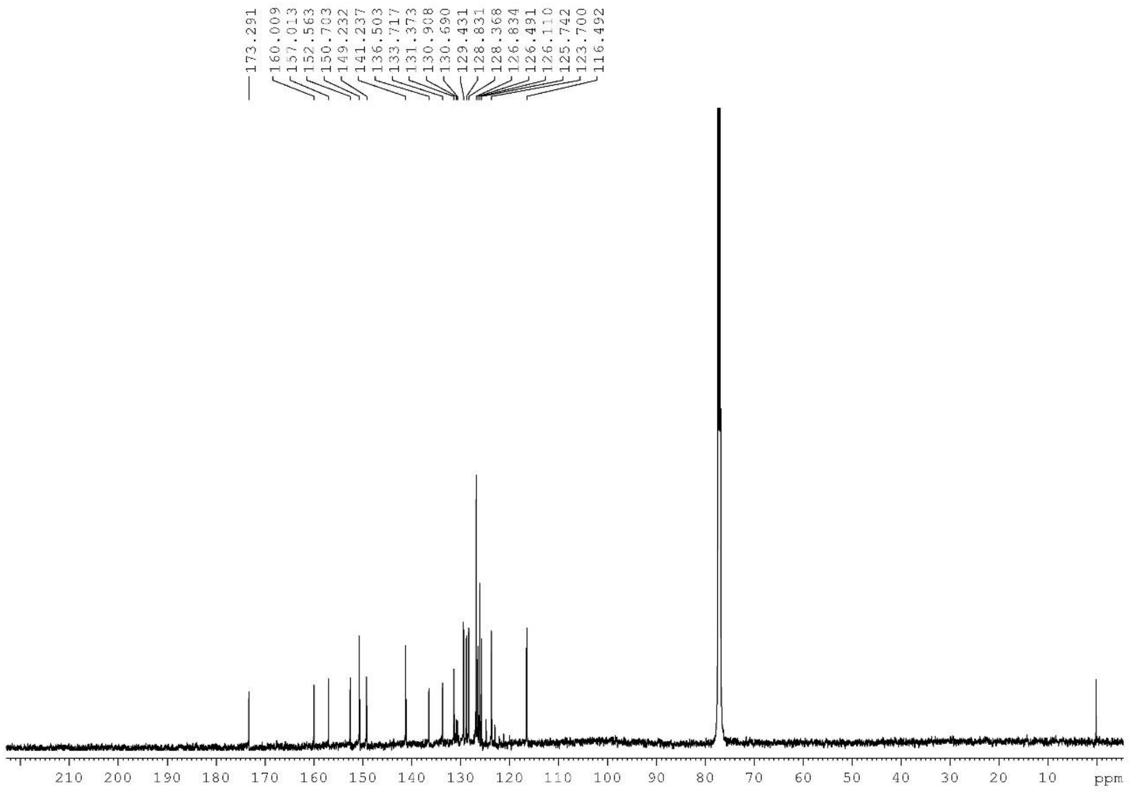

Figure S19. ^{13}C NMR spectrum of **Ir5b** in $CDCl_3$.

4. High Performance Liquid Cromatography-Mass spectrometry analysis

Table S1. HPLC method

Time (min)	0.1 % formic acid in H_2O	0.1 % formic acid in CH_3CN	Flow (mL/min)
**0**	80	20	0.4
**25**	0	100	

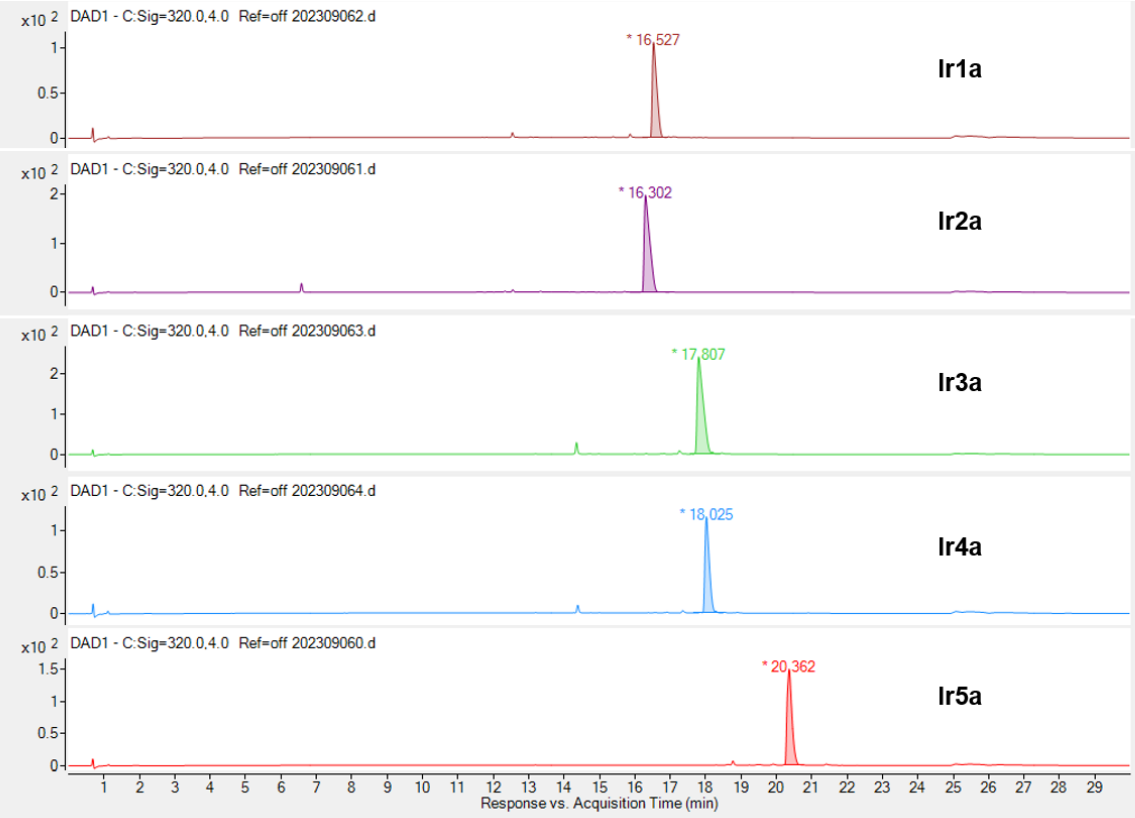

Figure S20. HPLC chromatograms with UV detection at 320 nm of **Ir1a-5a**.

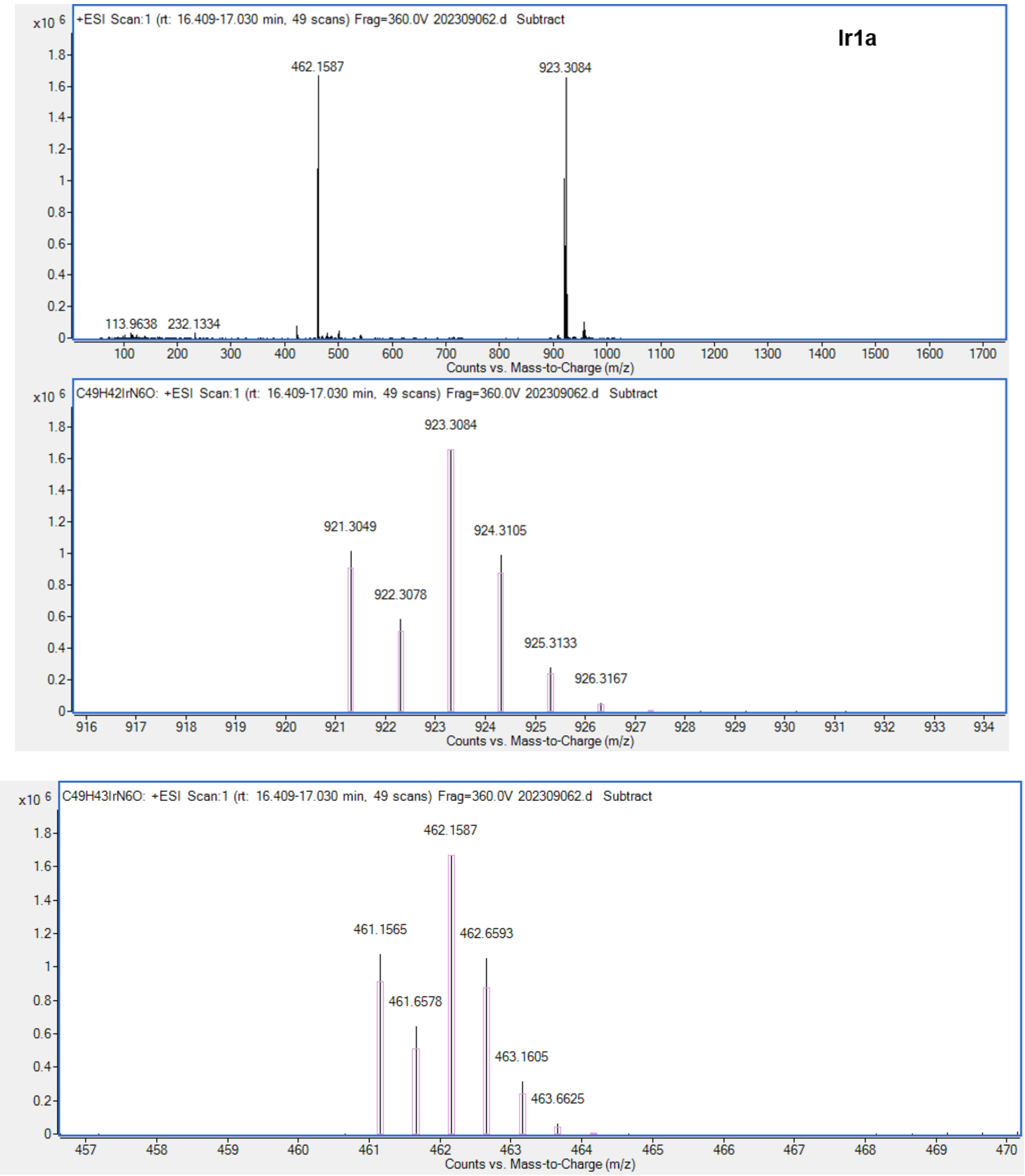

Figure S21. Mass spectra of the 16.5 min peak of chromatograms of **Figure S20** corresponding to the complex **Ir1a**.

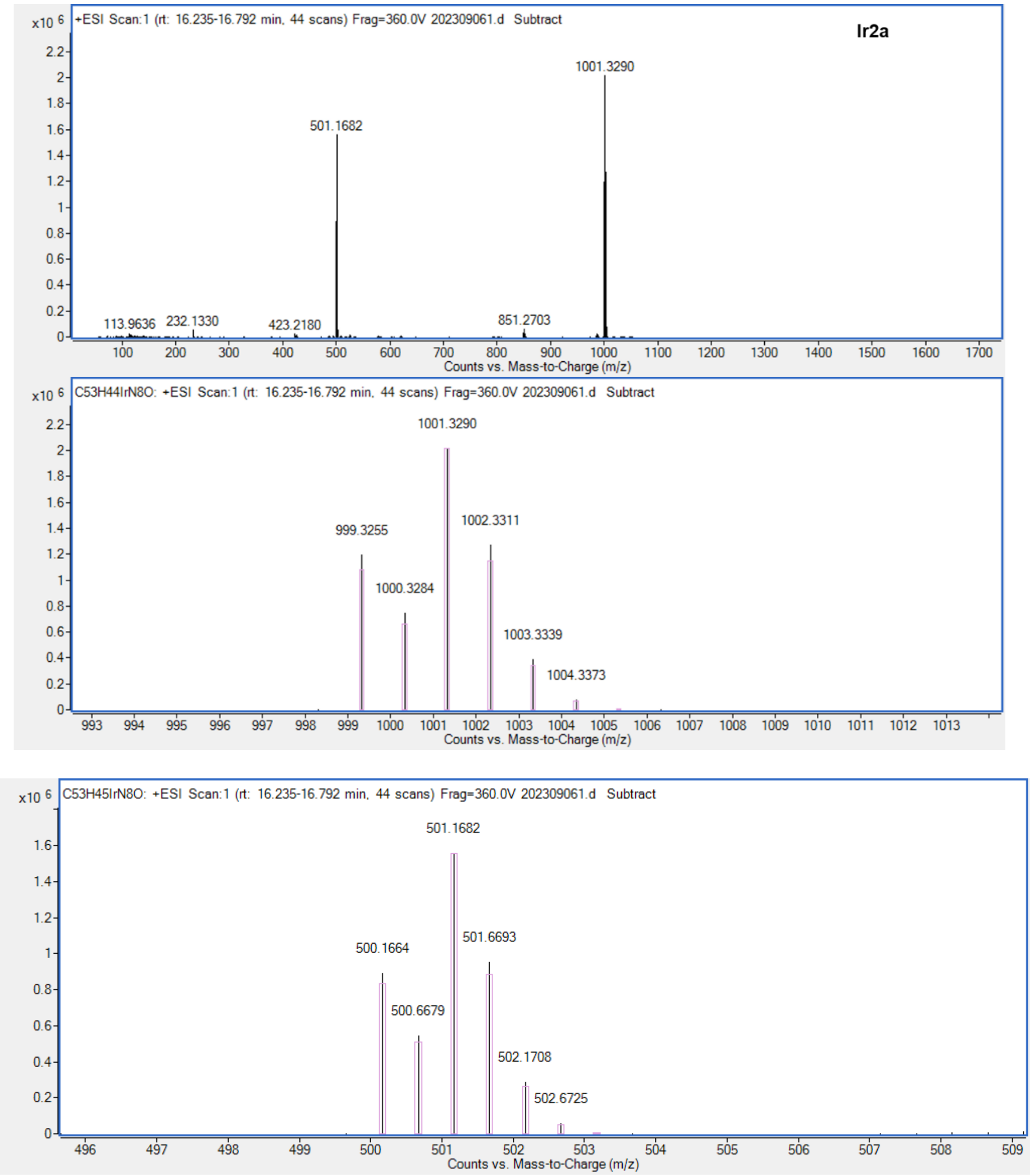

Figure S22. Mass spectra of the 16.3 min peak of chromatograms of **Figure S20** corresponding to the complex **Ir2a**.

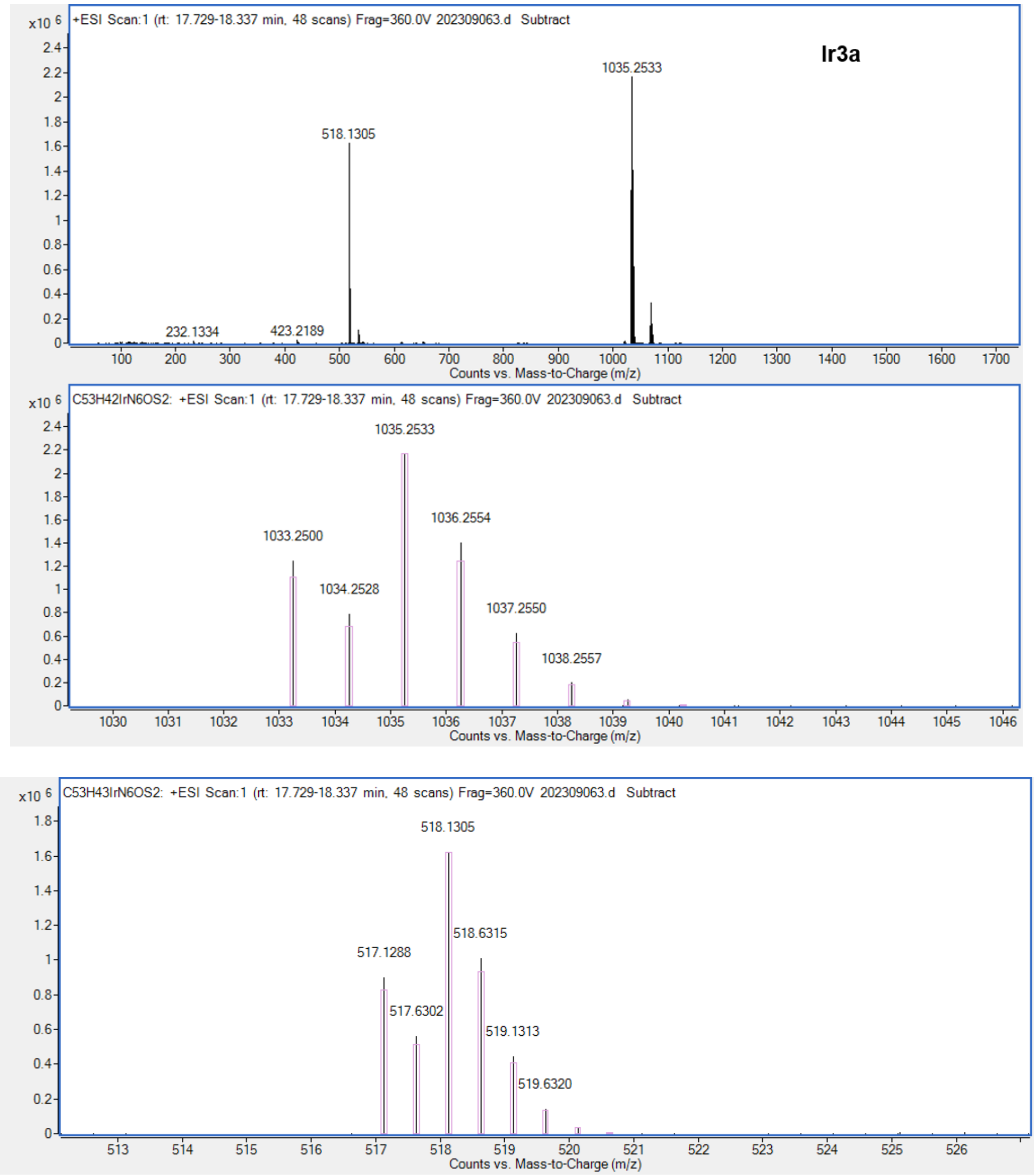

Figure S23. Mass spectra of the 17.8 min peak of chromatograms of **Figure S20** corresponding to the complex **Ir3a**.

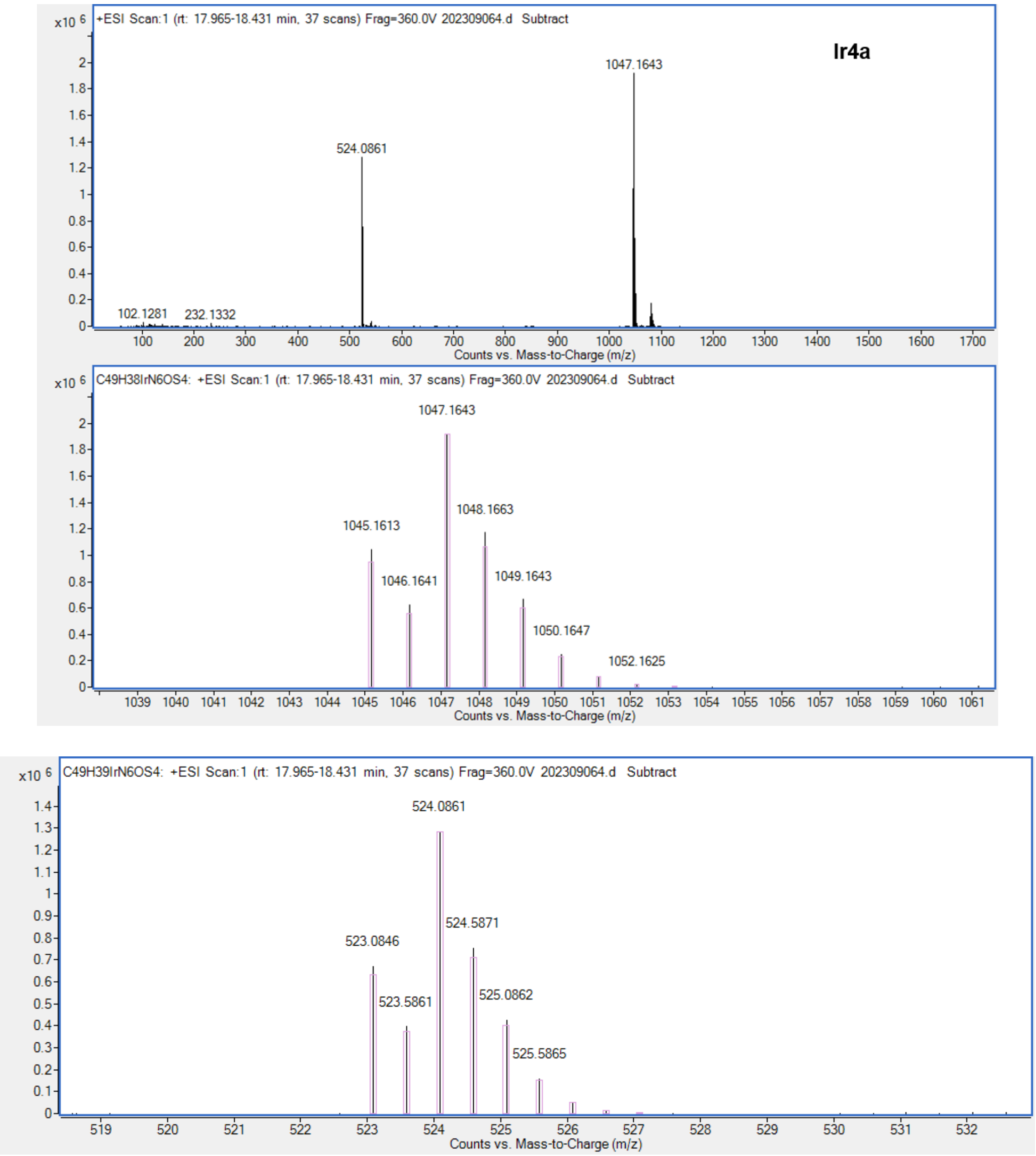

Figure S24. Mass spectra of the 18 min peak of chromatograms of **Figure S20** corresponding to the complex **Ir4a**.

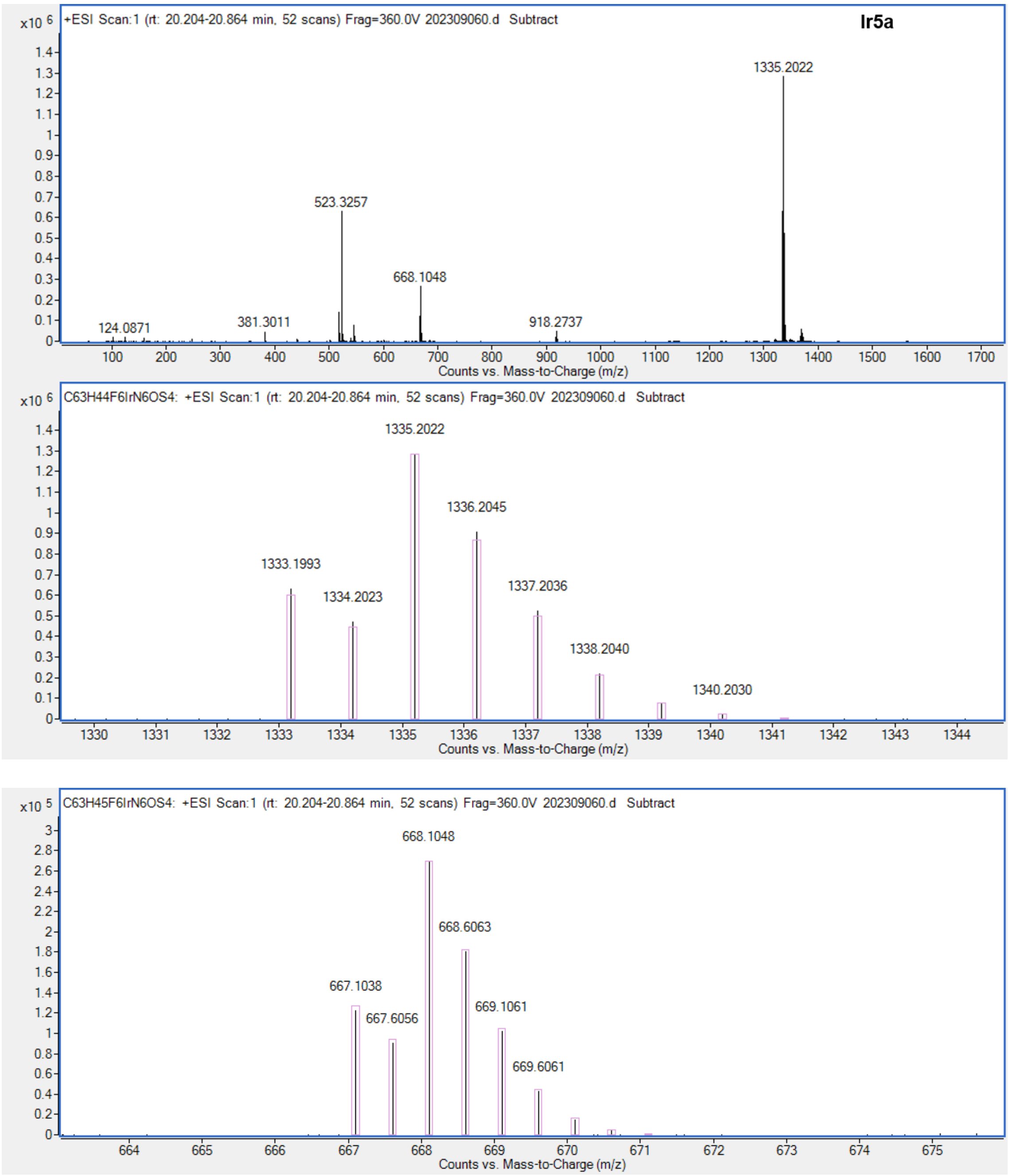

Figure S25. Mass spectra of the 20.3 min peak of chromatograms of **Figure S20** corresponding to the complex **Ir5a**.

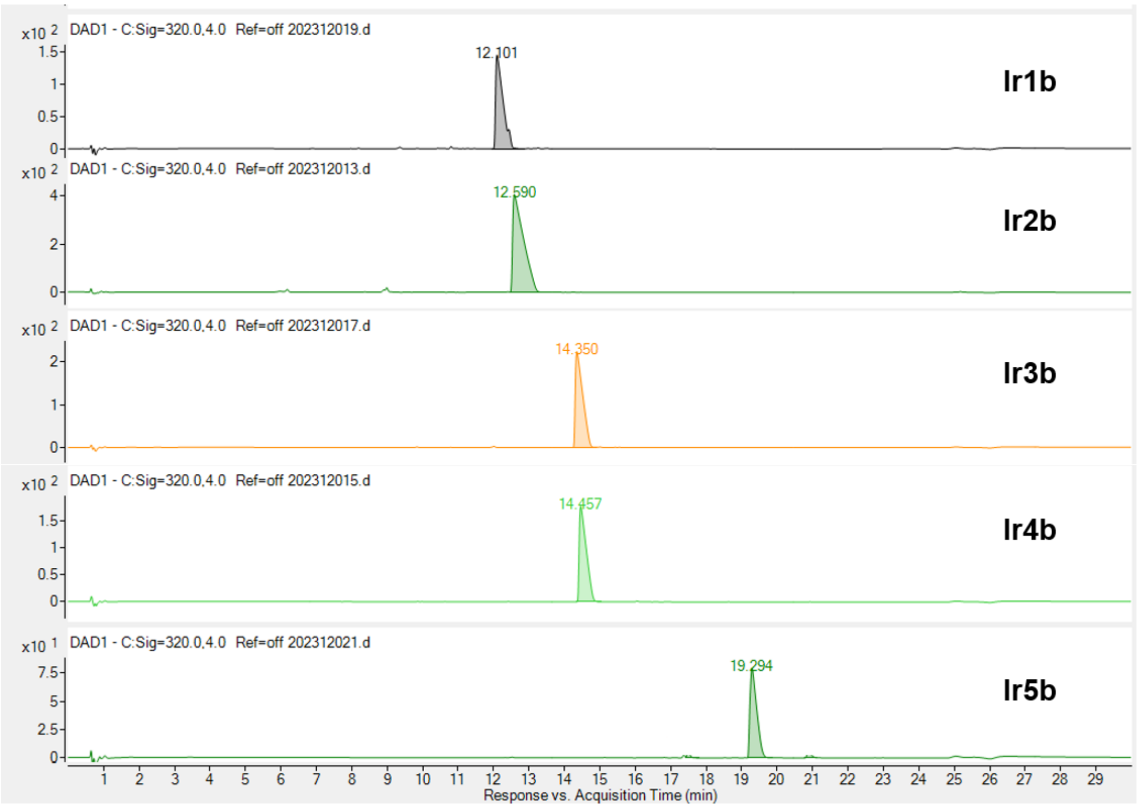

Figure S26. HPLC chromatograms with UV detection at 320 nm of **Ir1b-5b**.

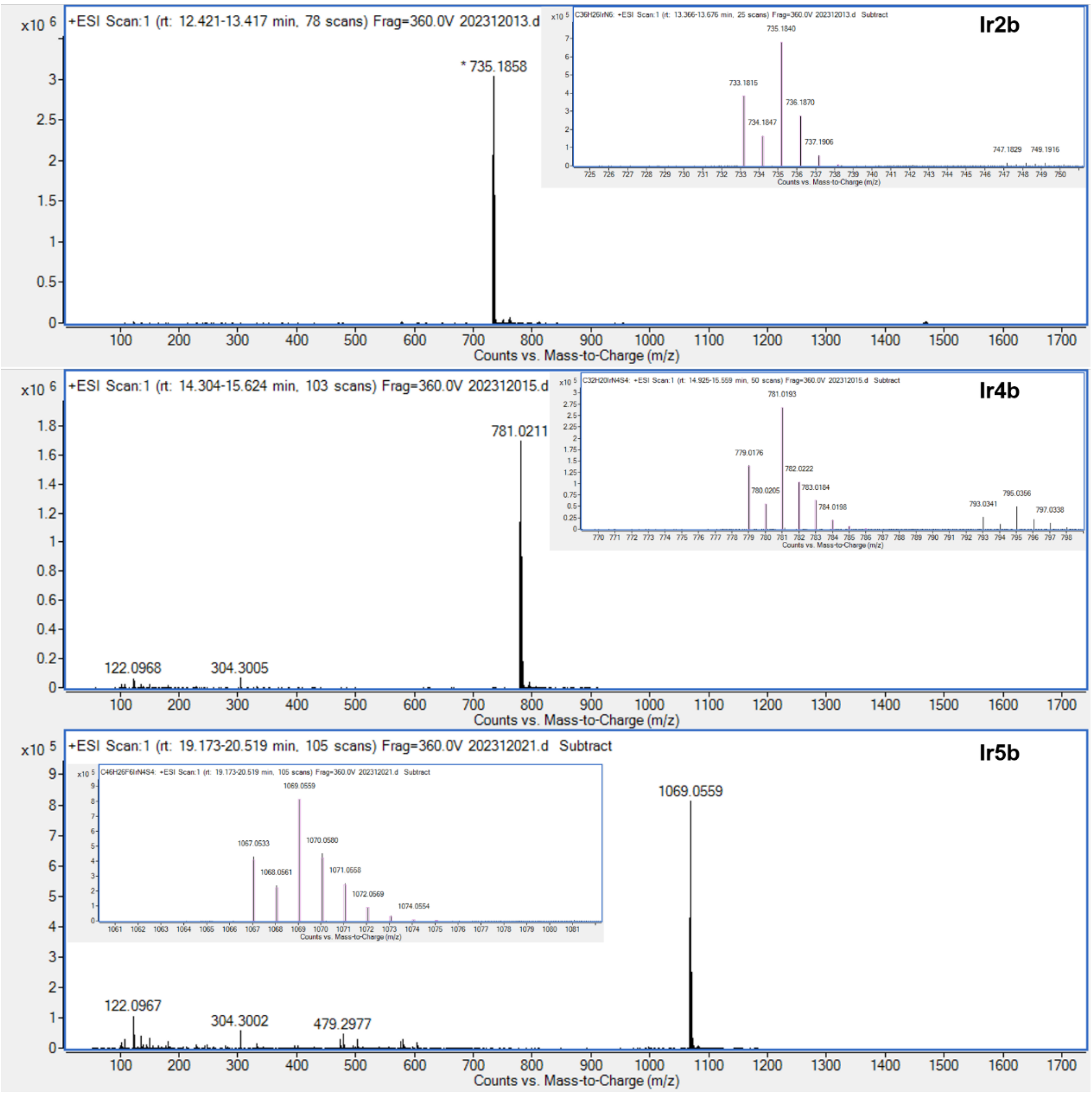

Figure S27. Mass spectra of the 12-19 min peak of chromatograms of **Figure S26** corresponding to the complexes **Ir2b**, **Ir4b** and **Ir5b**.

5. Photophysical properties

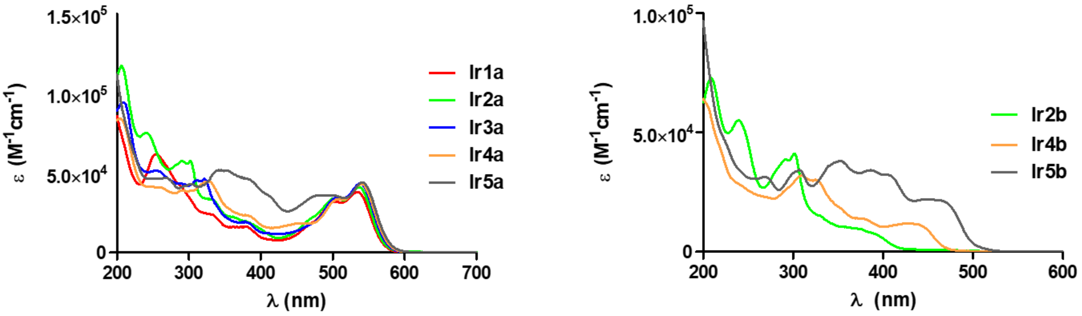

Figure S28. UV/Vis spectra of compounds Ir(III) complexes (10 µM) in acetonitrile.

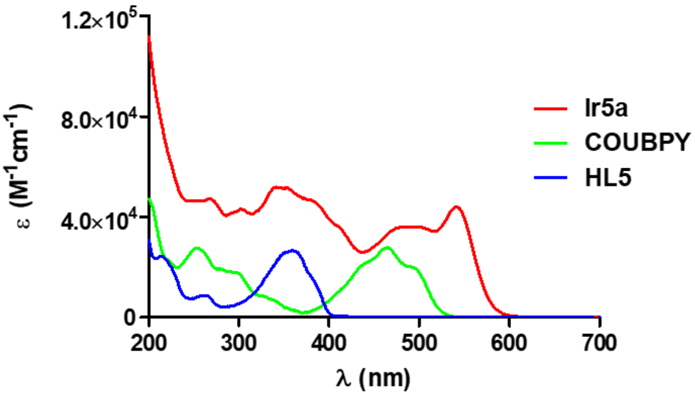

Figure S29. UV/Vis spectra of **HL5**, **COUBPY** ligand and **Ir5a** complex in acetonitrile.

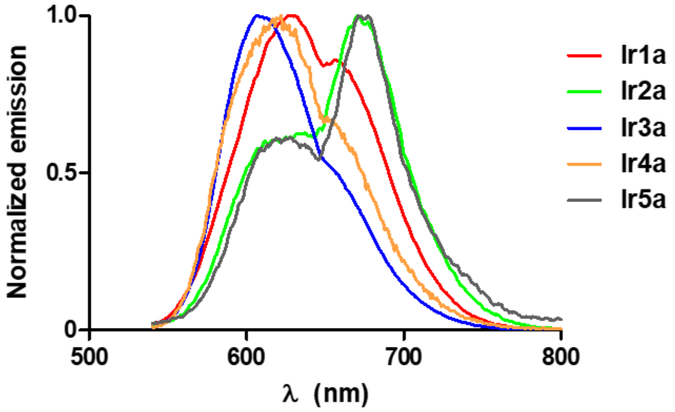

Figure S30. Emission spectra of **Ir1a-5a** in water (1 % DMSO).

Table S2. Absorption (λ_{abs}) and emission (λ_{em}) maxima wavelengths of Ir(III) complexes**,** lifetimes (τ_{em}) and emission quantum yields (Φ_{em}) in acetonitrile.

**Complex**	**λ_{abs}, nm (ε, $M^{-1}cm^{-1}$)**	**λ_{ex}, nm**	**λ_{em}, nm**	**τ_{em}, ns[a]**	**Φ_{em}[a]**
**Ir1a**	254 (61740), 332 (24050), 372 (16280), 503 (31970), 534 (38040)	500	584	778	<0,01
**Ir2a**	206 (117330), 240 (75300), 290 (57480), 302 (57520), 336 (32270), 503 (34230), 536 (41070)	500	578	1350 (21 %) 186 (79%)	<0,01
**Ir3a**	208 (94460), 254 (51410), 311 (45430), 321 (46450), 378 (19660), 506 (34550), 538 (43390)	500	588	2360	<0,01
**Ir4a**	204 (84210), 264 (40480), 292 (39000), 328 (44760), 451 (18470), 508 (33440), 539 (43140)	500	587	1750	<0,01
**Ir5a**	266 (47100), 302 (43120), 345 (51770), 499 (35980), 541 (44030)	540	586	266 (82 %) 10 (18 %)	<0,01
			668	1230	<0,01
**Ir1b[b]**	254 (41080), 310 (18100), 373 (4970)	380	586	280	0,11
**Ir2b**	208 (72760), 239 (55160), 292 (38590), 302 (41160), 380[h] (8920)	380	580	633	0,17
**Ir3b[b]**	270 (31735), 309 (28953), 409 (6882)	365	524, 560[h]	n.d.	0,03
**Ir4b**	309 (32000), 325 (30170), 377 (13970), 427 (11900)	370	575 621[h]	4940	0,36
**Ir5b**	269 (31470), 307 (34230), 352 (38060), 386 (34190), 405 (32290), 450[h] (22030)	470	669	1240	<0,01

[a]Lifetimes and emisión quantum yields measured in the absence of oxygen. [b]Values obtained from the literature.[3–5] n.d.: not determined.

Table S3. Absorption (λ_{abs}) and emission (λ_{em}) wavelengths of complexes **Ir1a-5a** in water (1 % DMSO).

Complex	λ_{abs}, nm (ε, $M^{-1}cm^{-1}$)	λ_{ex}, nm	λ_{em}, nm
**Ir1a**	259 (39330), 339^{sh} (18170), 392^{sh} (13790), 524 (19380), 564 (16640)	520	627<br>656
**Ir2a**	305 (32570), 515 (17500), 558 (15440)	520	628 sh<br>671
**Ir3a**	314 (48910), 380 (20990), 327 (47820), 380 (20990), 521 (29960), 554 (30740)	520	607
**Ir4a**	327 (39800), 394^{sh} (21530), 448^{sh} (14850), 524 (24420), 558 (26740)	520	622<br>655 sh
**Ir5a**	304 (30760), 347 (36760), 481 (20000), 554 (22900)	520	627<br>677

6. Dark and light stability studies

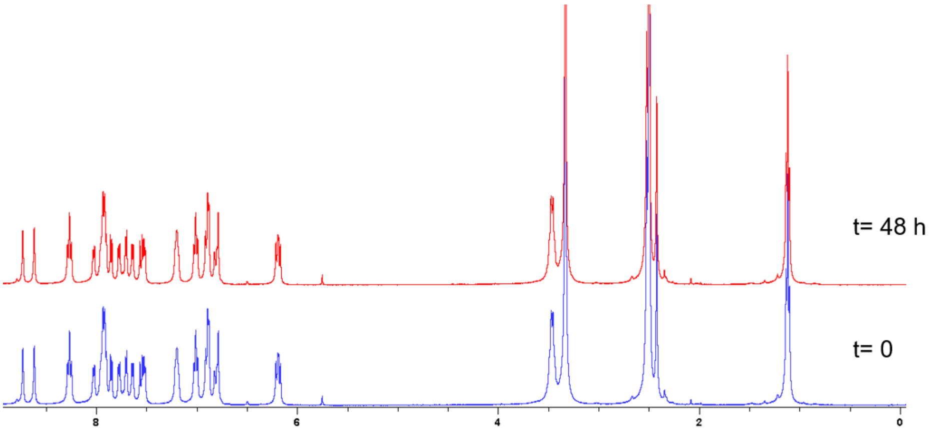

Figure S31. ^{1}H-NMR spectra of **Ir1a** in DMSO-d_6 at t=0 and after 48 h at room temperature.

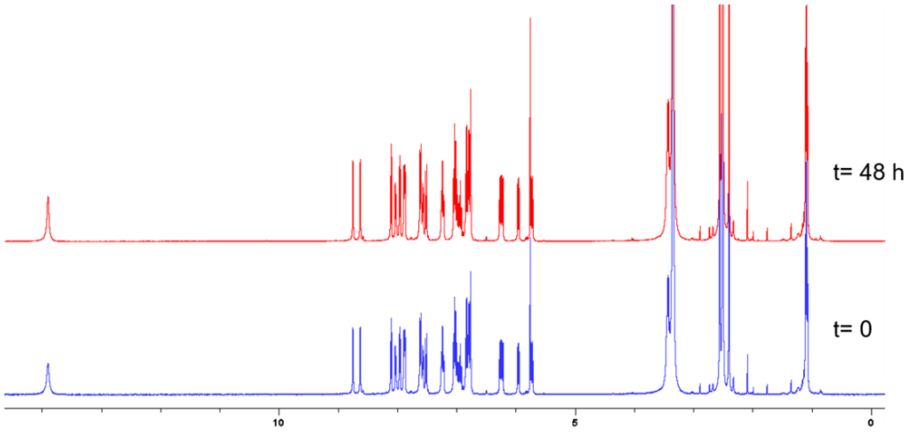

Figure S32. ^{1}H-NMR spectra of **Ir2a** in DMSO-d_6 at t=0 and after 48 h at room temperature.

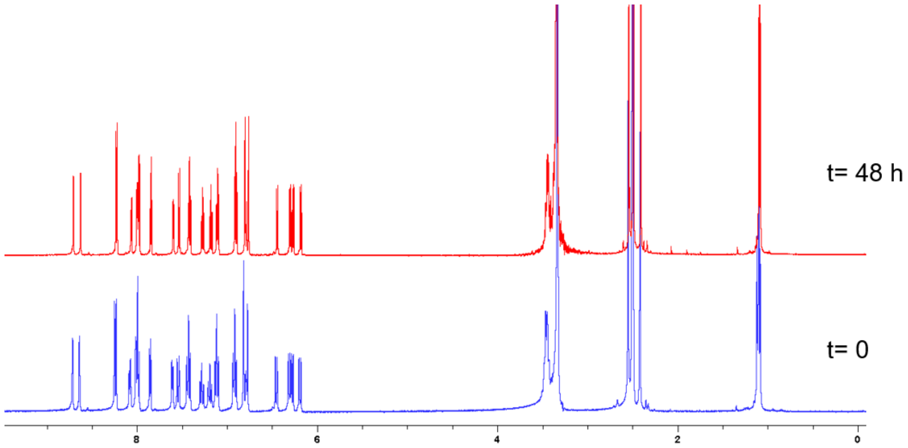

Figure S33. ^{1}H-NMR spectra of **Ir3a** in DMSO-d_6 at t=0 and after 48 h at room temperature.

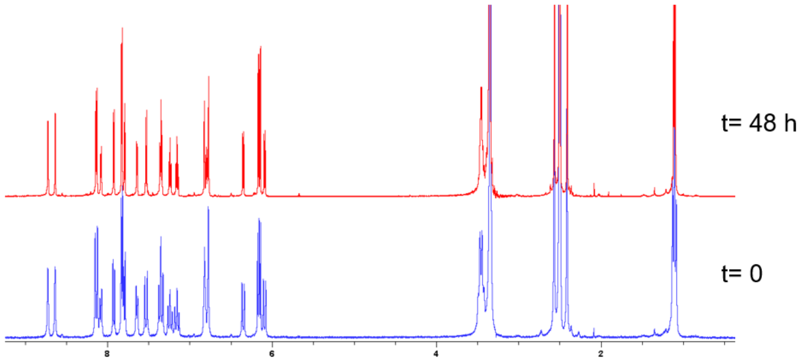

Figure S34. ^{1}H-NMR spectra of **Ir4a** in DMSO-d_6 at t=0 and after 48 h at room temperature.

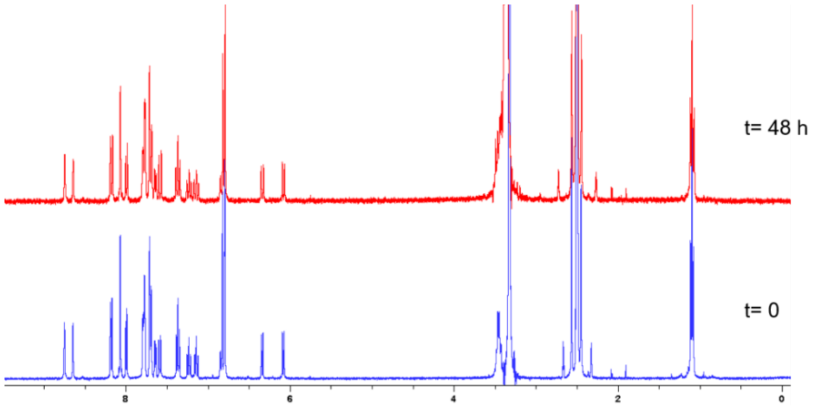

Figure S35. ^{1}H-NMR spectra of **Ir5a** in DMSO-d_6 at t=0 and after 48 h at room temperature.

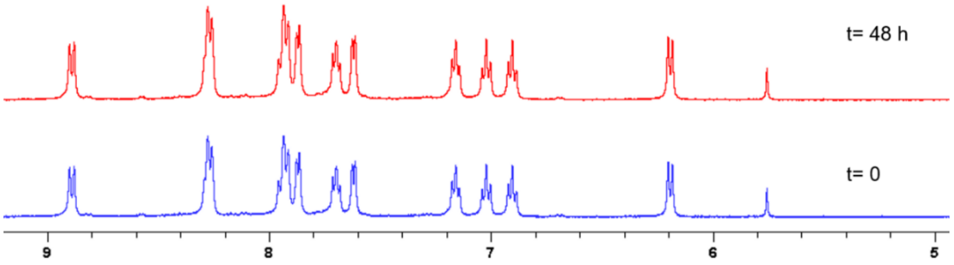

Figure S36. ^{1}H-NMR spectra of **Ir1b** in DMSO-d_6 at t=0 and after 48 h at room temperature.

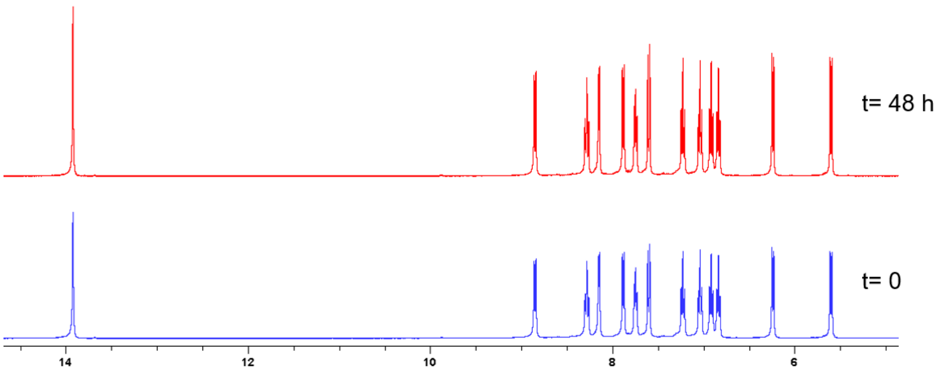

Figure S37. ^{1}H-NMR spectra of **Ir2b** in DMSO-d_6 at t=0 and after 48 h at room temperature.

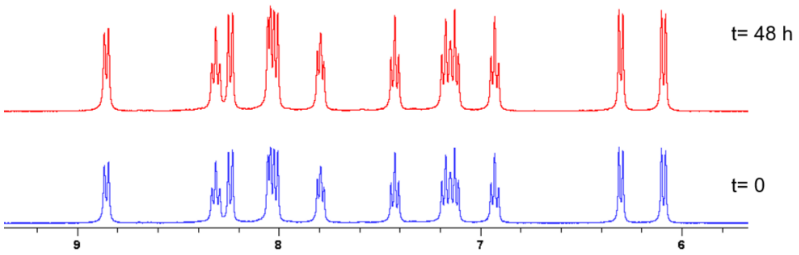

Figure S38. ^{1}H-NMR spectra of **Ir3b** in DMSO-d_6 at t=0 and after 48 h at room temperature.

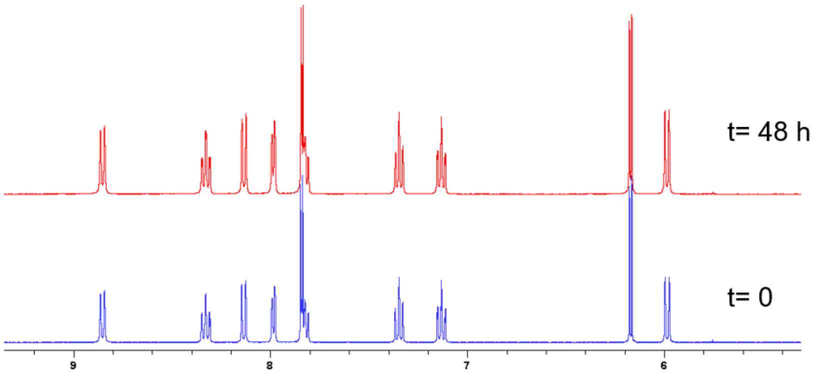

Figure S39. ^{1}H-NMR spectra of **Ir4b** in DMSO-d_6 at t=0 and after 48 h at room temperature.

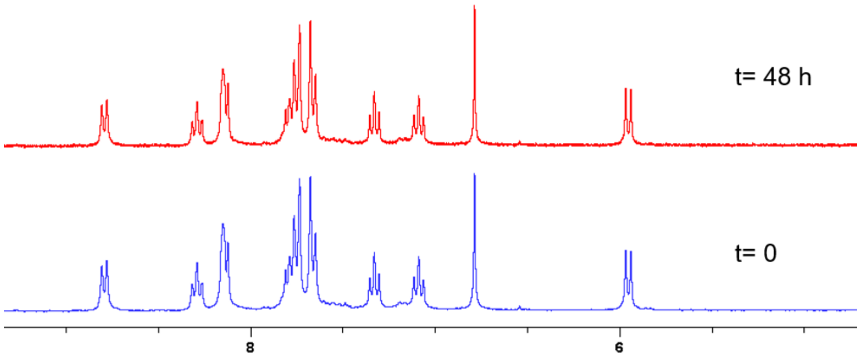

Figure S40. ^{1}H-NMR spectra of **Ir5b** in DMSO-d_6 at t=0 and after 48 h at room temperature.

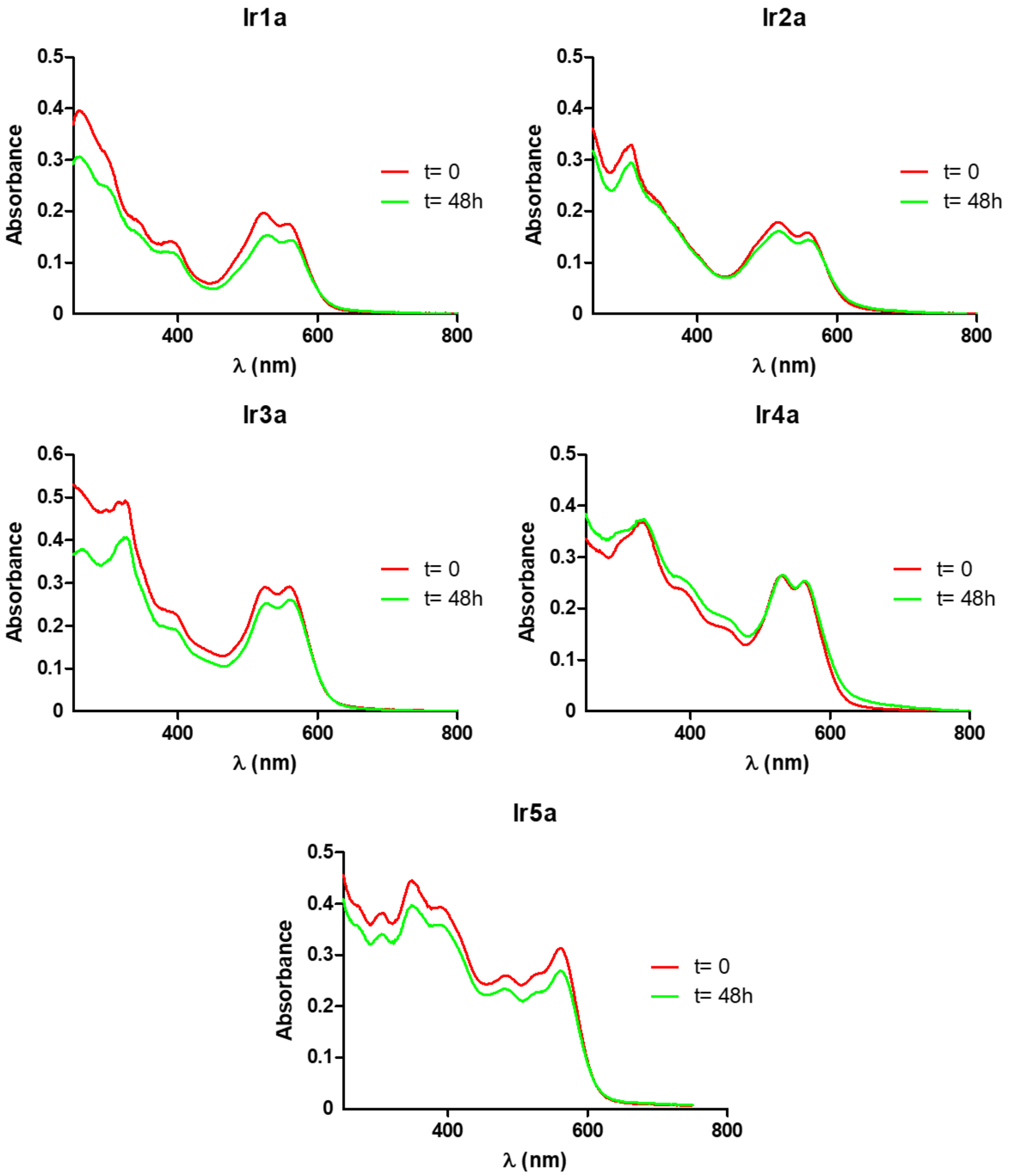

Figure S41. UV/Vis spectra of **Ir1a-5a** in RPMI (5 % DMSO) at t=0 and after 48 h at 37 ºC.

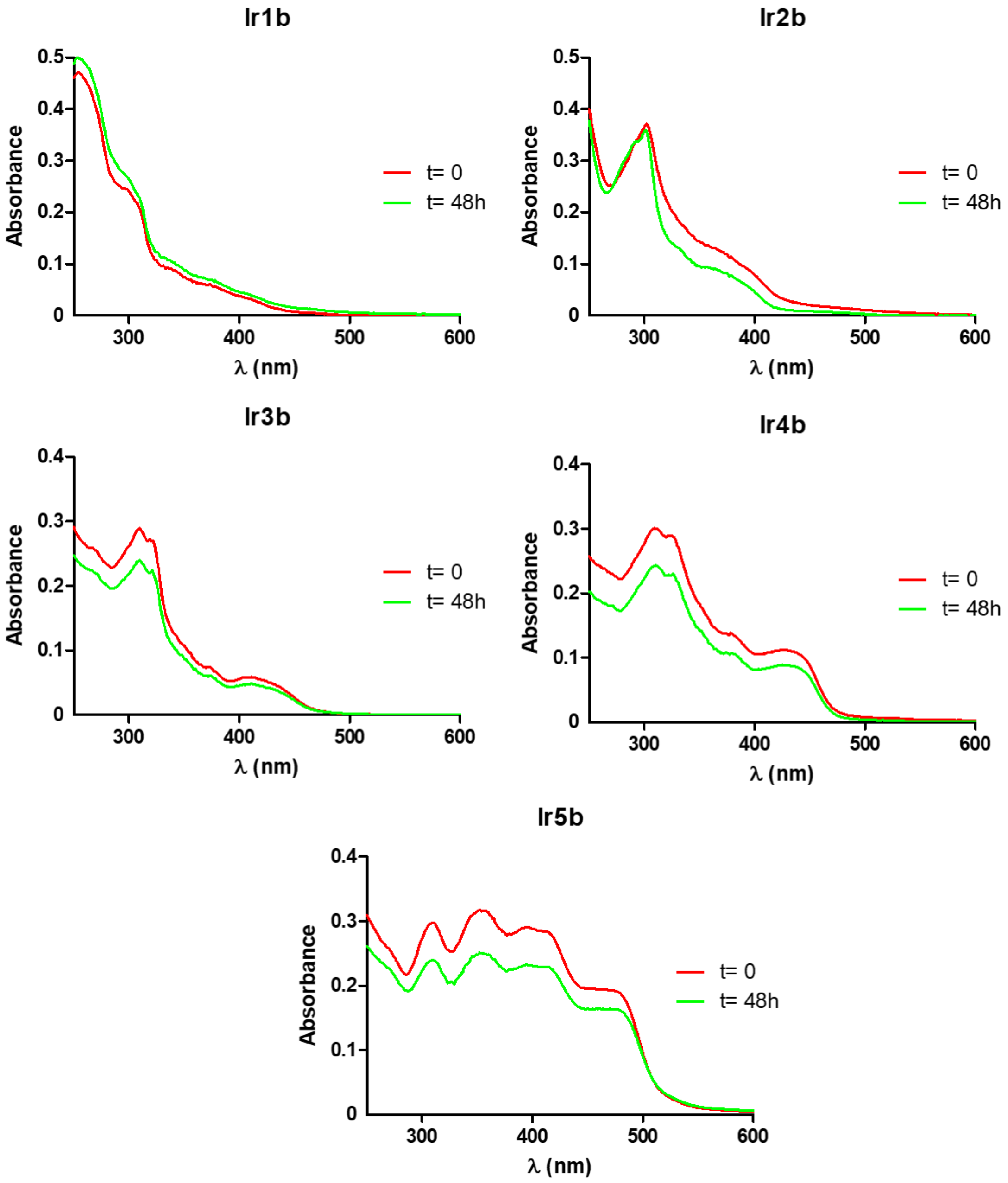

Figure S42. UV/Vis spectra of **Ir1b-5b** complexes in RPMI (5 % DMSO) at t= 0 and after 48 h at 37 ºC.

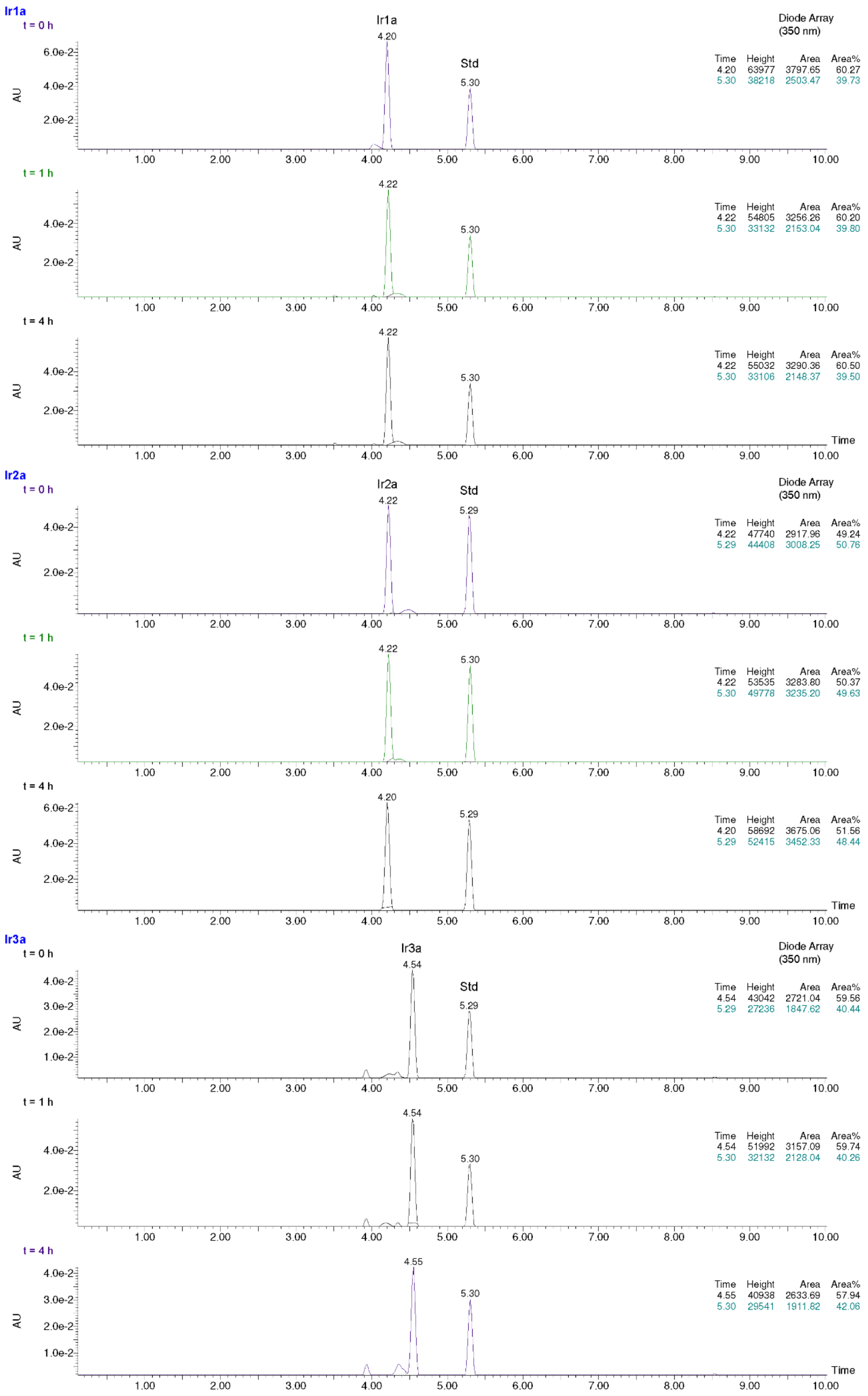

Figure S43. Stability of complexes **Ir1a–3a** after 4 h incubation in RPMI culture medium at 37 °C under dark conditions. Elution traces were obtained at 280 nm.

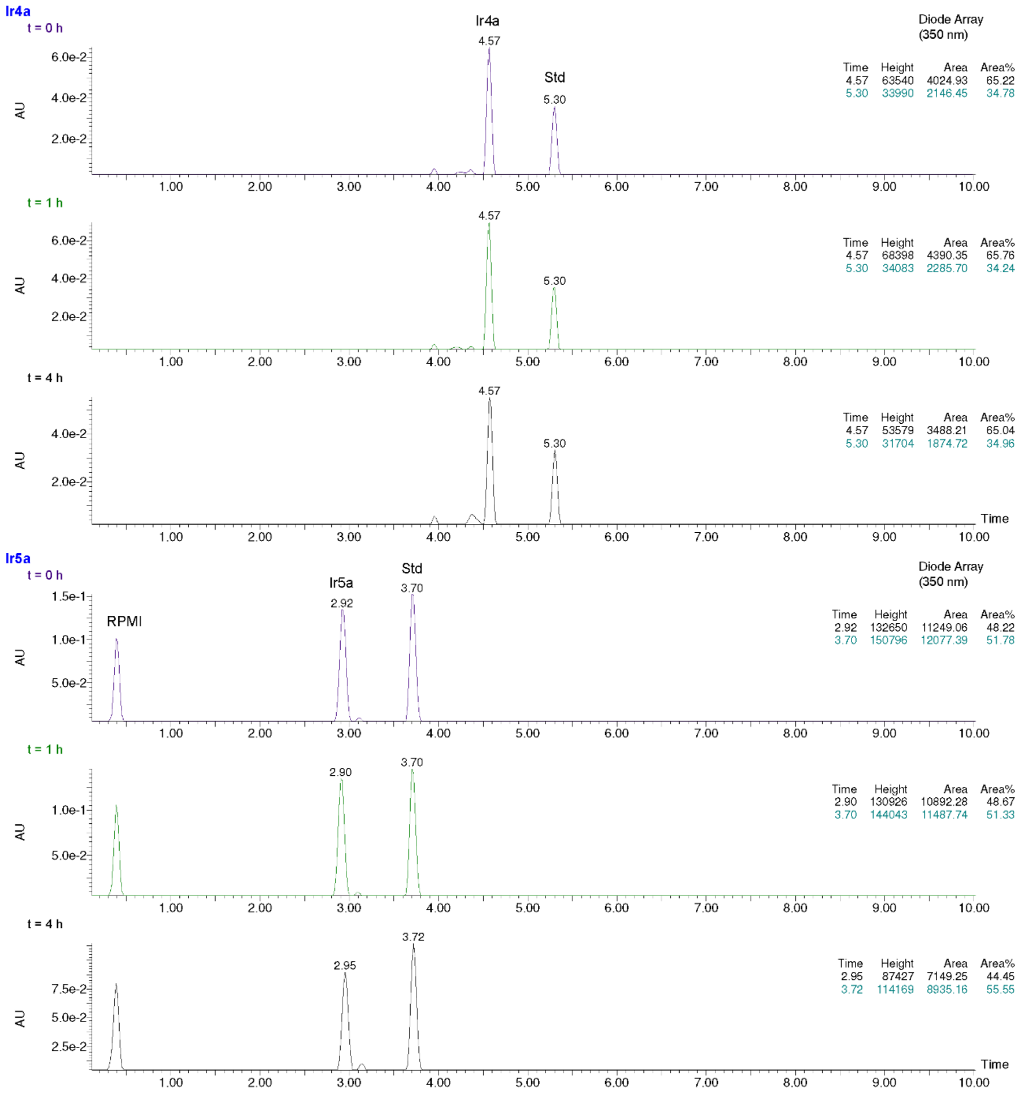

Figure S44. Stability of complexes **Ir4a** and **5a** after 4 h incubation in RPMI culture medium at 37 °C under dark conditions. Elution traces were obtained at 280 nm.

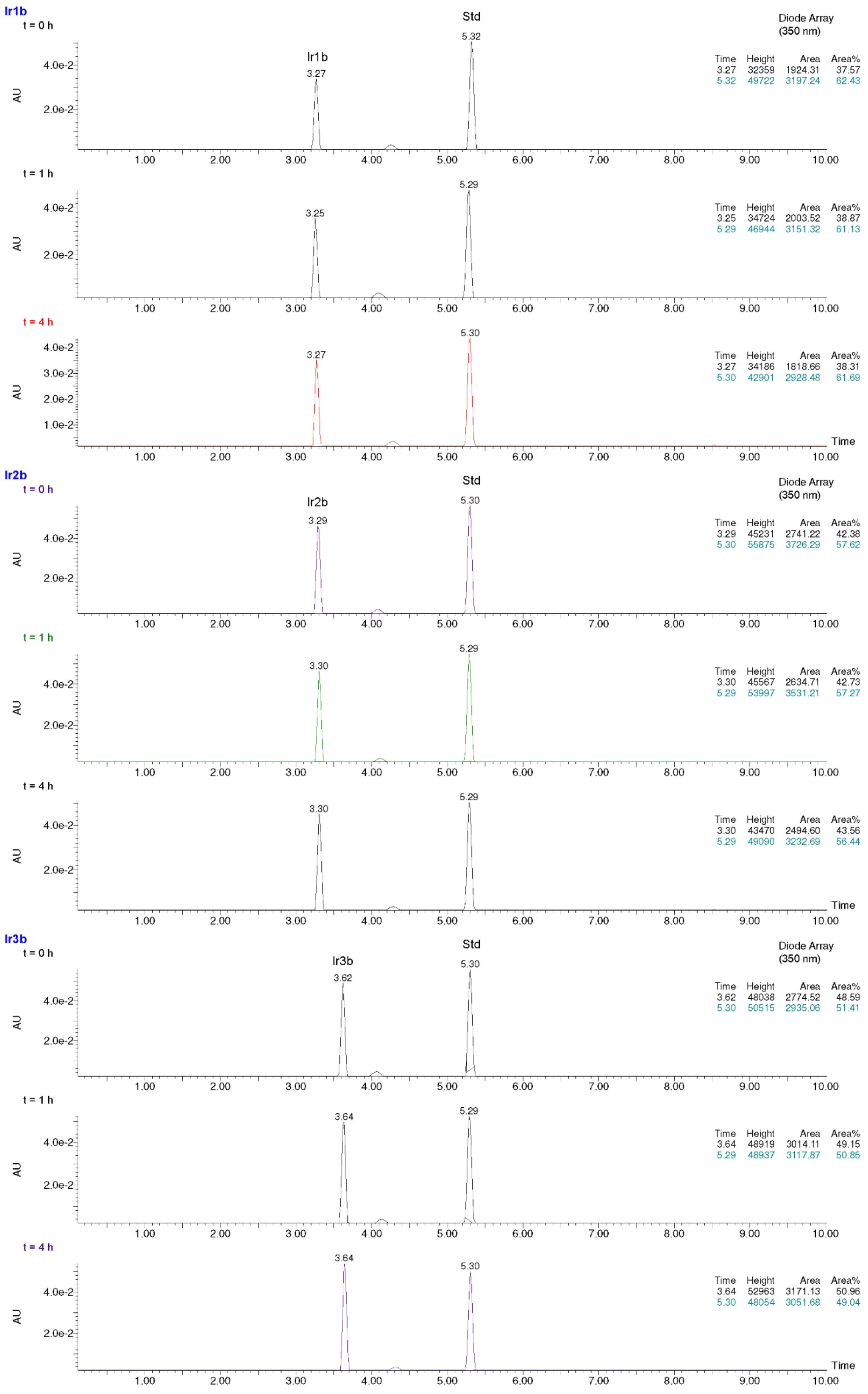

Figure S45. Stability of complexes **Ir1b–3b** after 4 h incubation in RPMI culture medium at 37 °C under dark conditions. Elution traces were obtained at 280 nm.

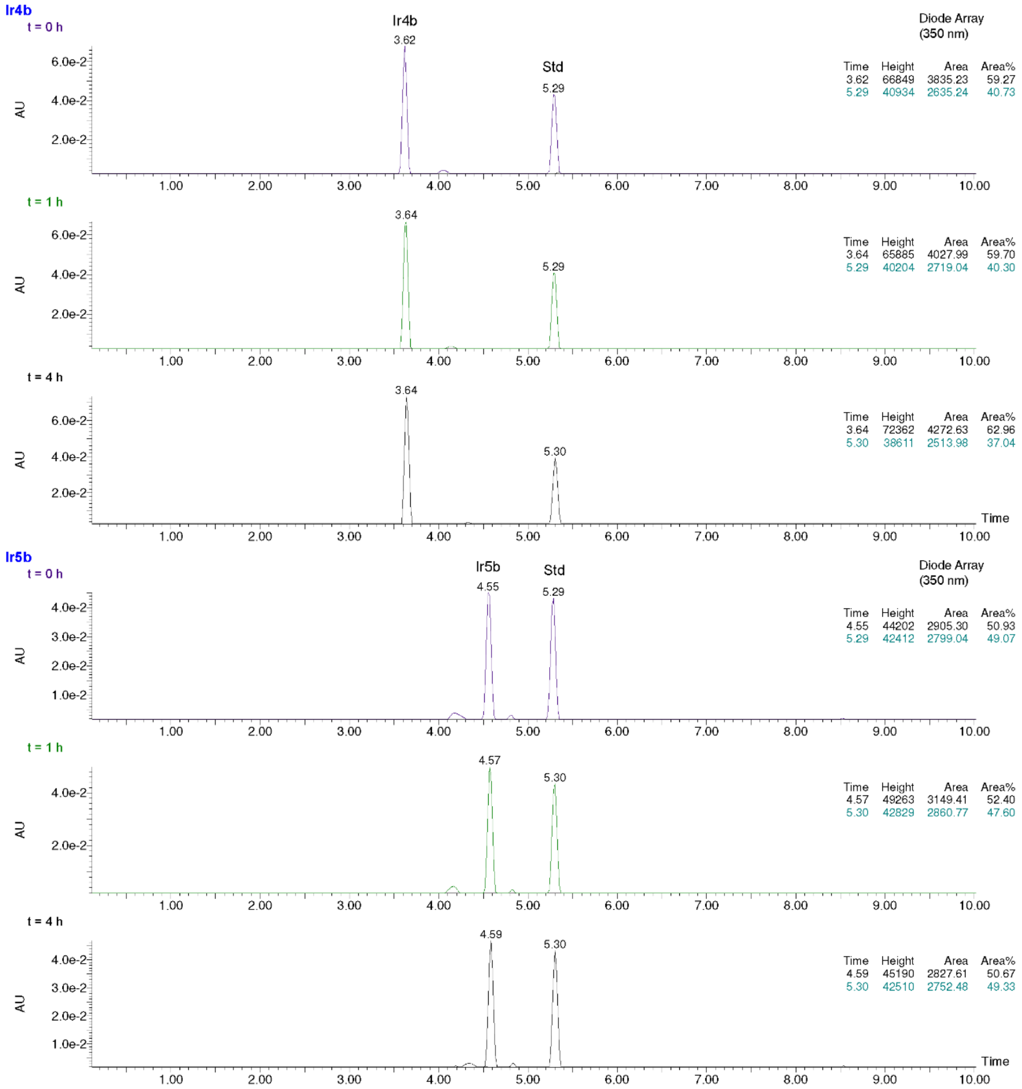

Figure S46. Stability of complexes **Ir4b** and **5b** after 4 h incubation in RPMI culture medium at 37 °C under dark conditions. Elution traces were obtained at 280 nm.

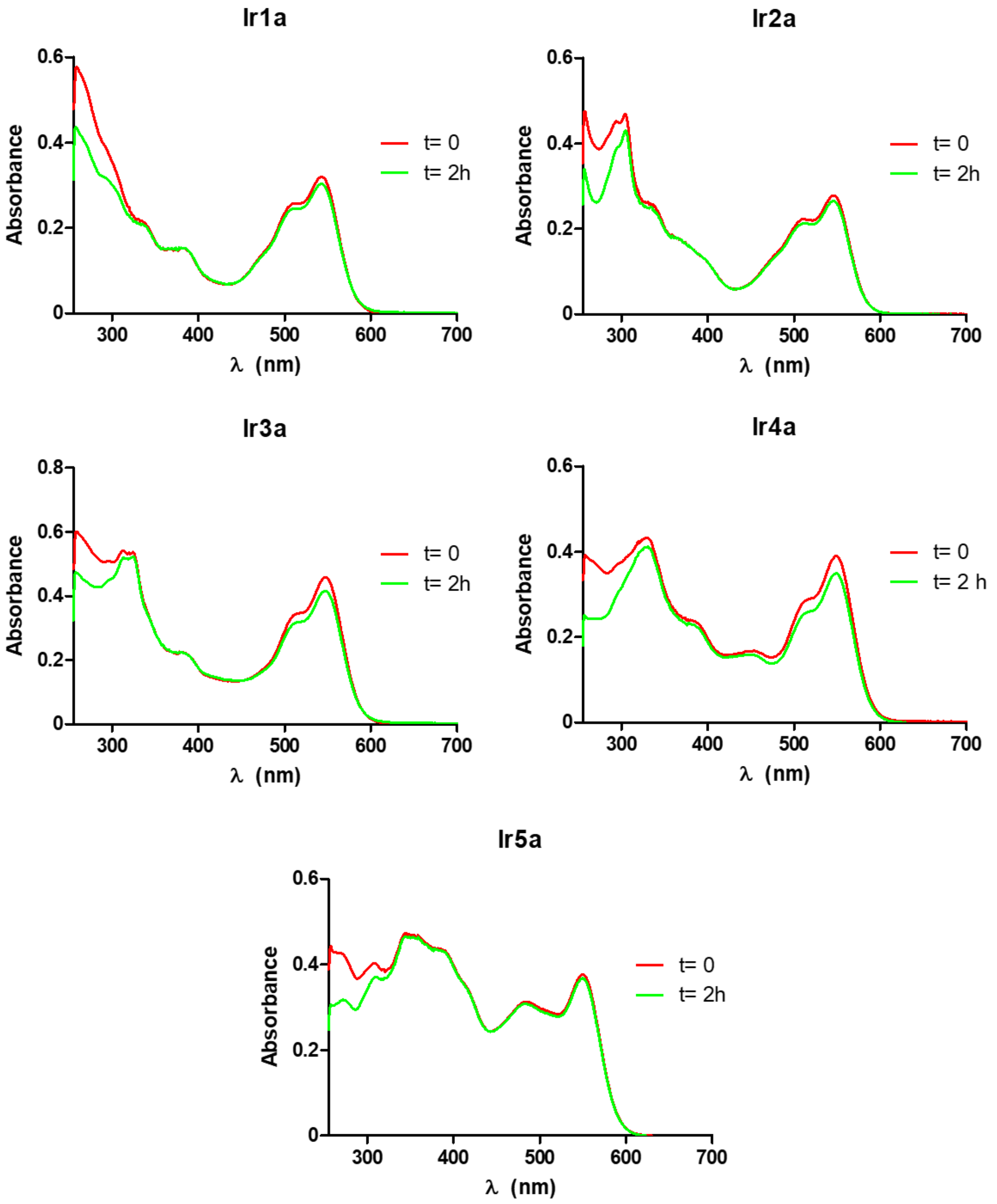

Figure S47. UV/Vis spectra of **Ir1a-5a** complexes in DMSO at t=0 and after 2 h of green light irradiation (520 nm, 1.8 mW/cm^2).

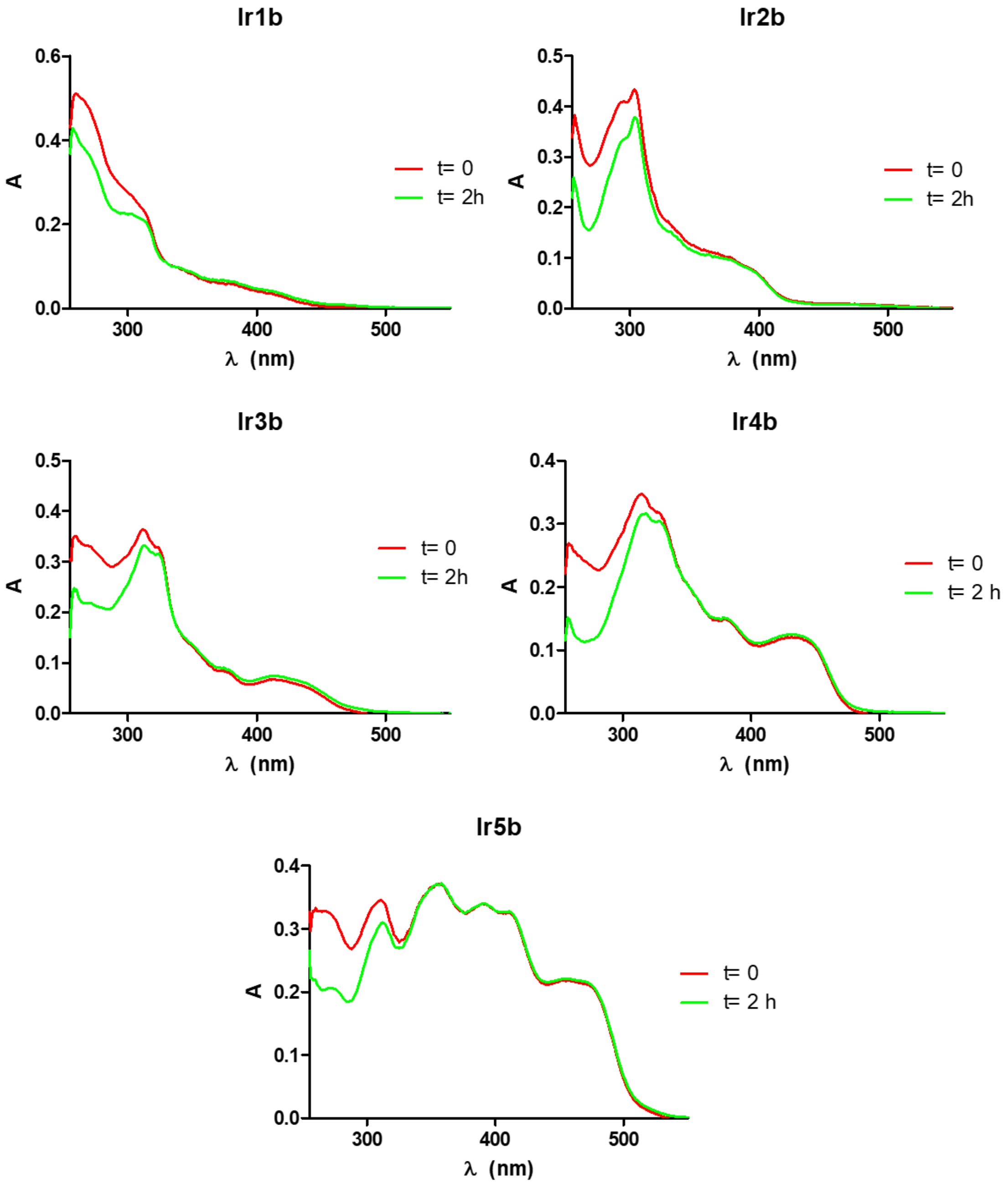

Figure S48. UV/Vis spectra of **Ir1b-5b** complexes in DMSO at t=0 and after 2 h of blue light irradiation (465 nm, 4.0 mW/cm^2).

7. NADH photo-oxidation and evaluation for $^1O_2$ and/or •OH/ $^{•}O_2^-$ generation in cell free media

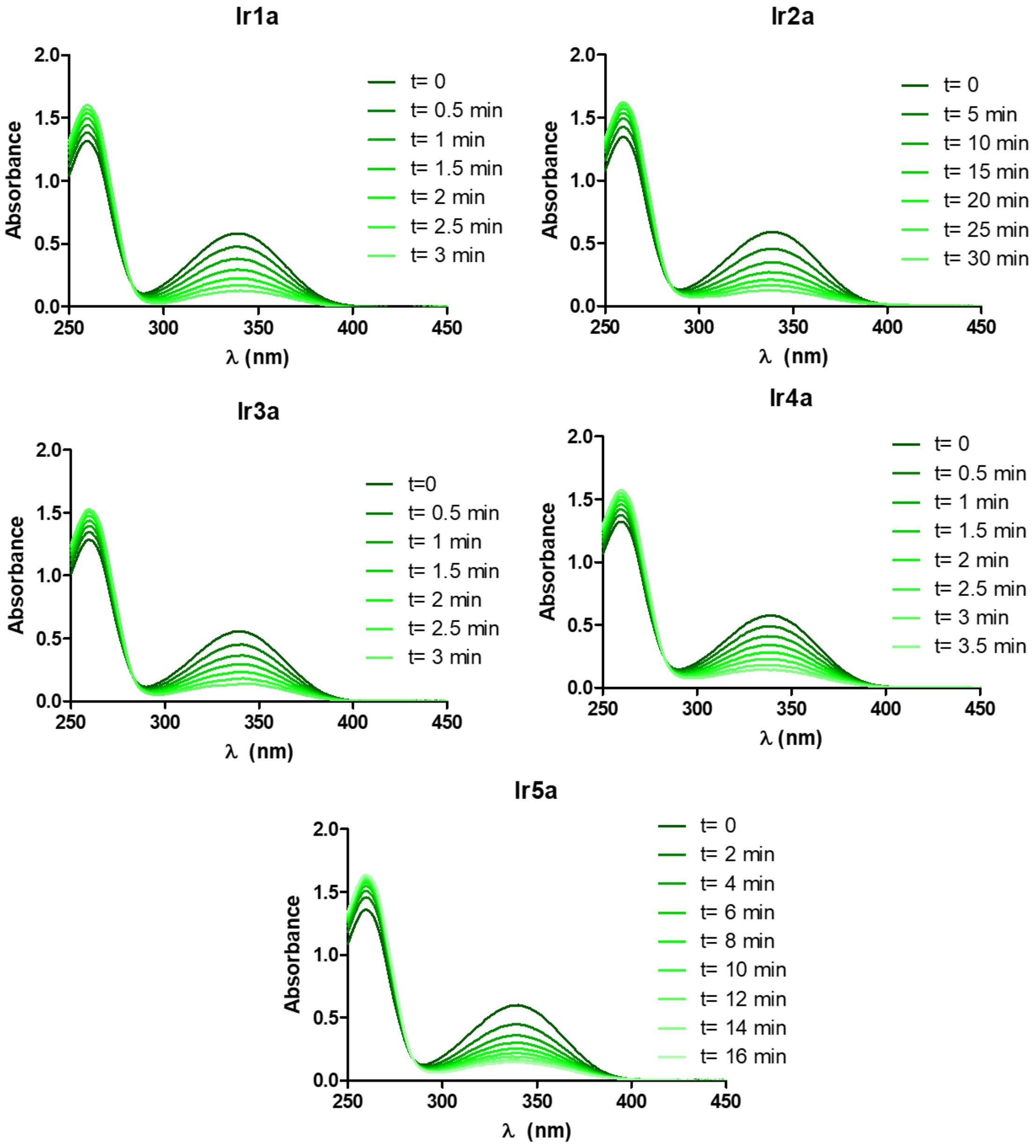

Figure S49. NADH absorption spectra (100 µM) in the presence of **Ir1a-5a** complexes (5 µM) in PBS (5 % DMF) under green light irradiation (520 nm, 2.0 mW/cm^2).

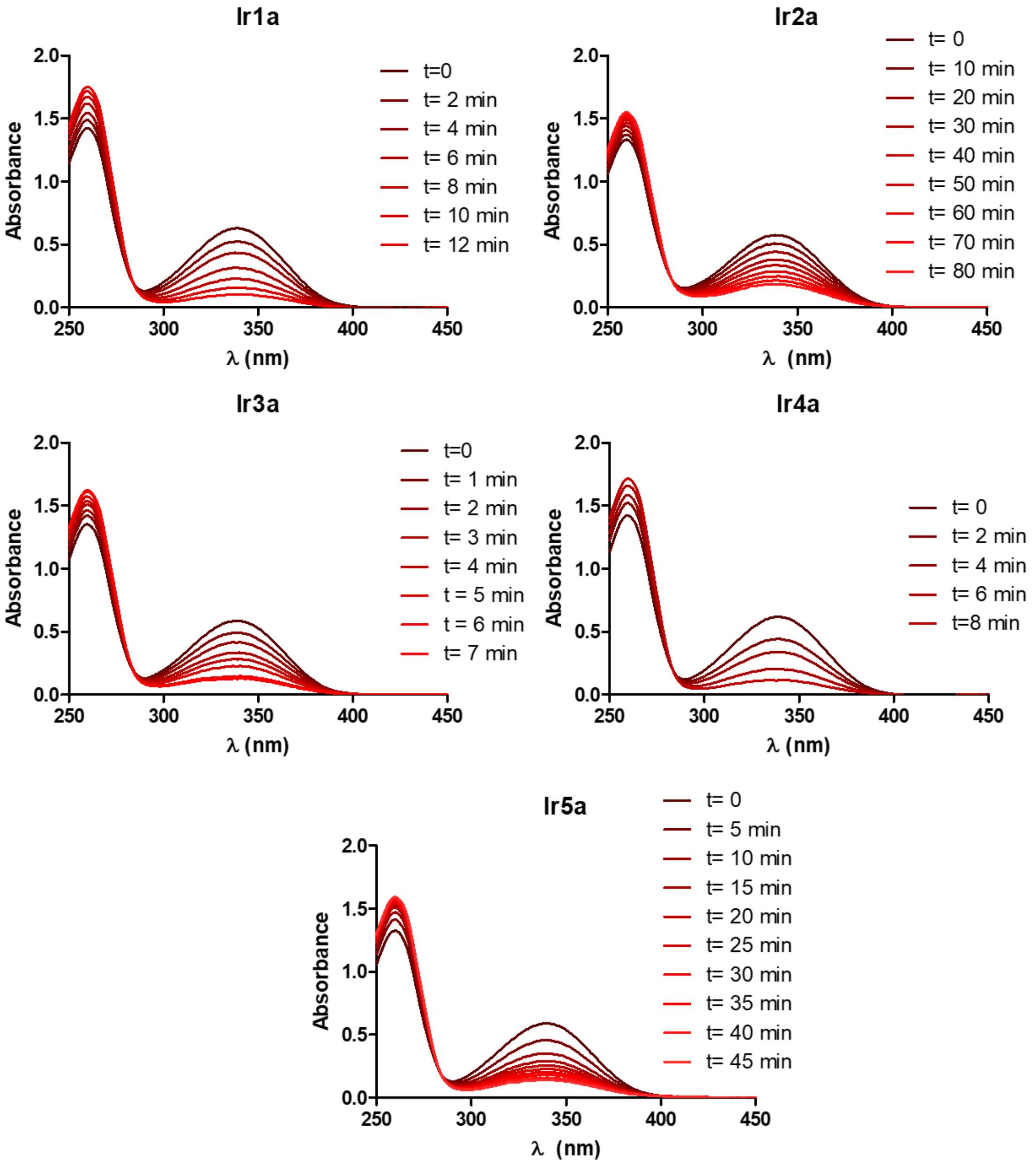

Figure S50. NADH absorption spectra (100 µM) in the presence of **Ir1a-5a** complexes (5 µM) in PBS (5 % DMF) under red light irradiation (620 nm, 15 mW/cm^2).

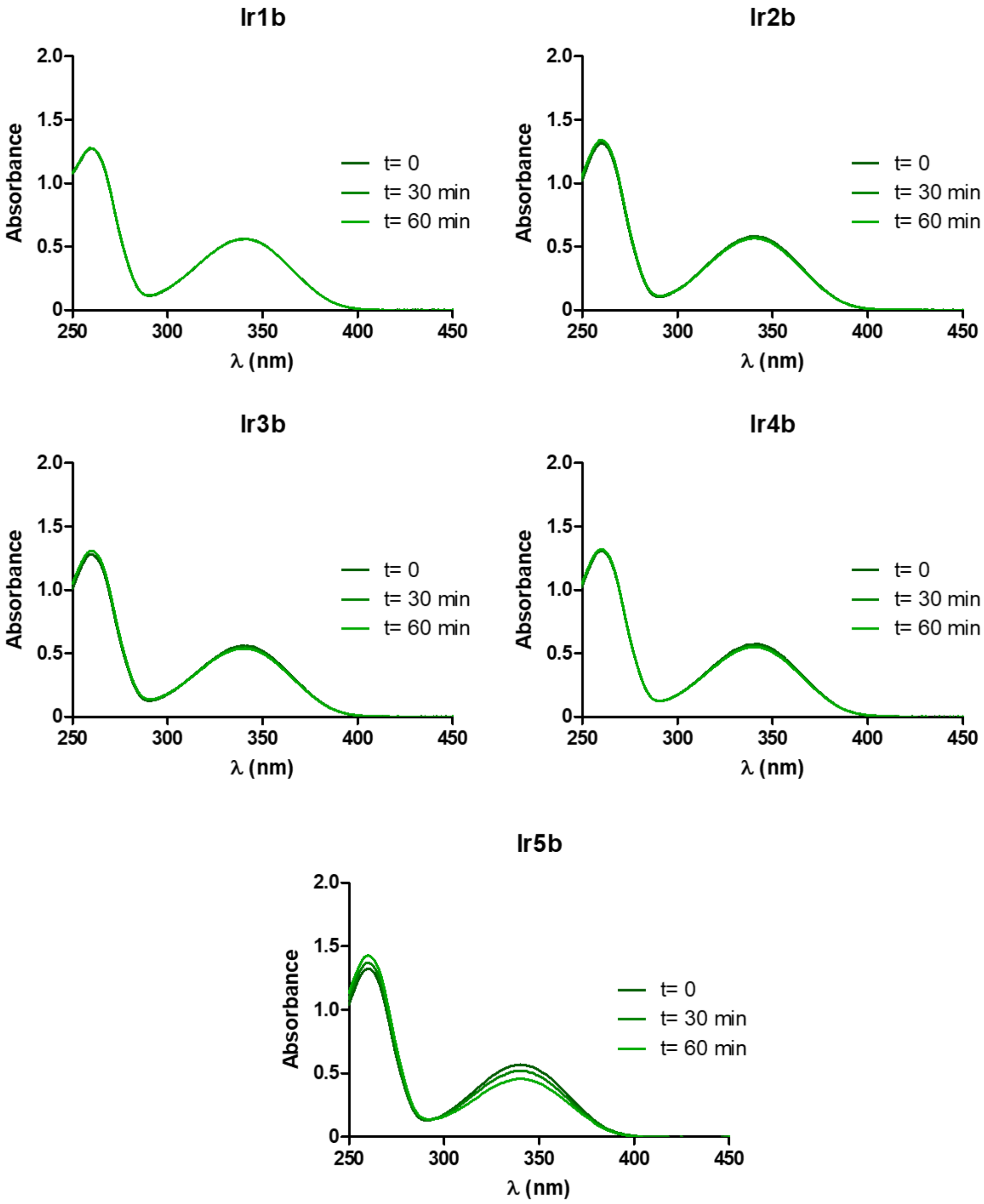

Figure S51. NADH absorption spectra (100 µM) in the presence of **Ir1b-5b** complexes (5 µM) in PBS (5 % DMF) under green light irradiation (520 nm, 2.0 mW/cm^2).

Table S4. TOF Values and Singlet Oxygen Quantum Yields of Ir1a-Ir5a and Ir1b-Ir5b complexes.

**Compound**	**TOF (h^{-1})**		Φ_{Δ}[d]
	λ= 620 nm[a]	λ= 520 nm[b]	
**Ir1a**	84.35	292.09	0.31
**Ir2a**	9.42	29,79	0.16
**Ir3a**	126.61	242.12	0.15
**Ir4a**	121.47	237.90	0.46
**Ir5a**	19.19	54.80	0.79
	λ= 465 nm[c]	λ= 520 nm[b]	Φ_{Δ}[e]
**Ir1b**	0.45	0	0.38
**Ir2b**	7.04	0.50	0.50
**Ir3b**	18.98	0.73	0.75
**Ir4b**	25.07	0.75	0.95
**Ir5b**	49.89	3.57	0.67

[a] Red light potency: 15 mW/cm^2. [b] Green light potency: 2 mW/cm^2.
[c] Blue light potency: 4 mW/cm^2. [d] Green light potency: 0.5 mW/cm^2.
[e] Blue light potency: 0.5 mW/cm^2.

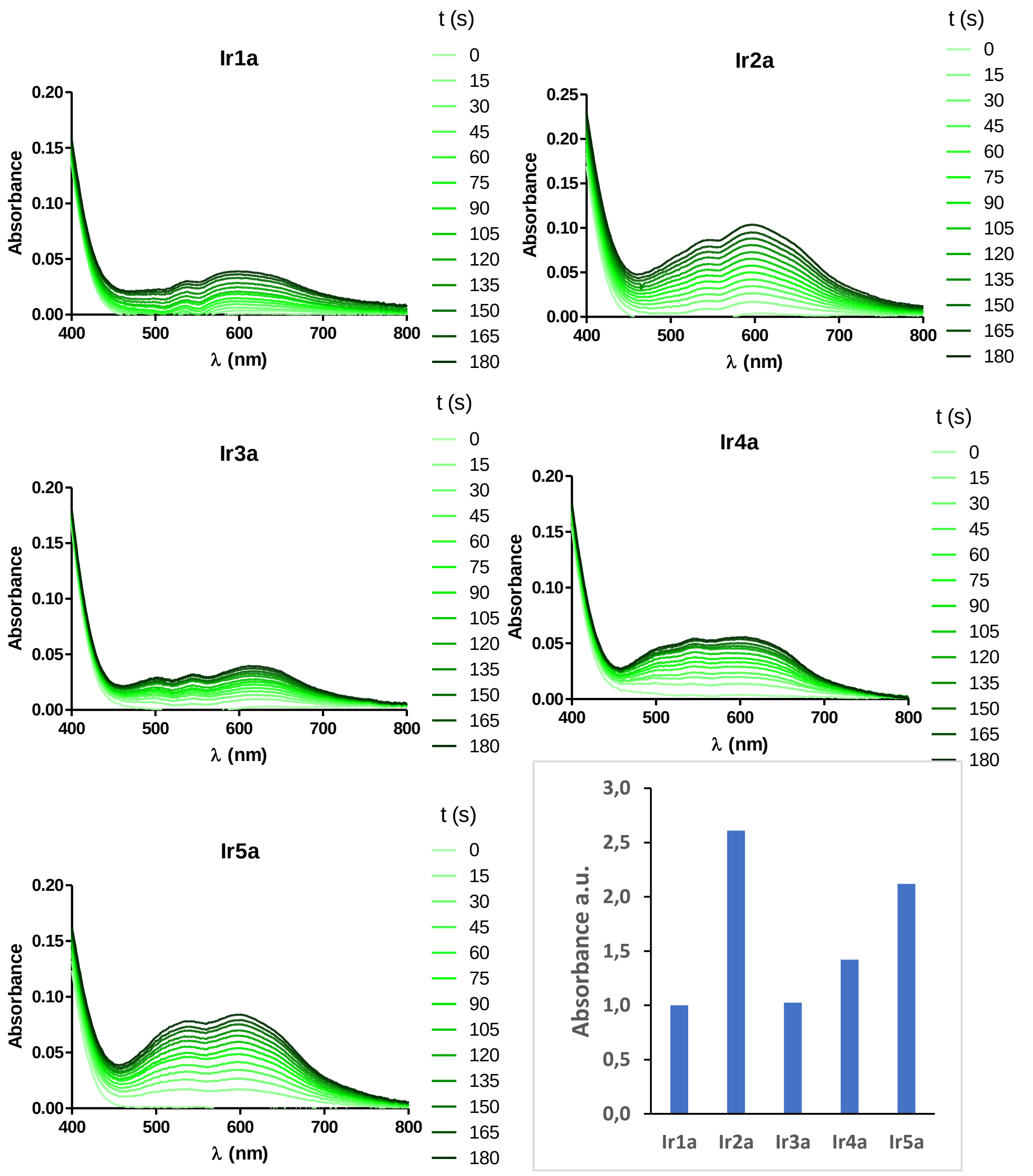

Figure S52. Monitoring of the absorption profile of NBT (50 µM) upon incubation with complexes **Ir1a-Ir5a** (5 µM) and NADH (100 µM) and green light irradiation (λ = 520 nm, 1.0 mW/cm^2) at short intervals.

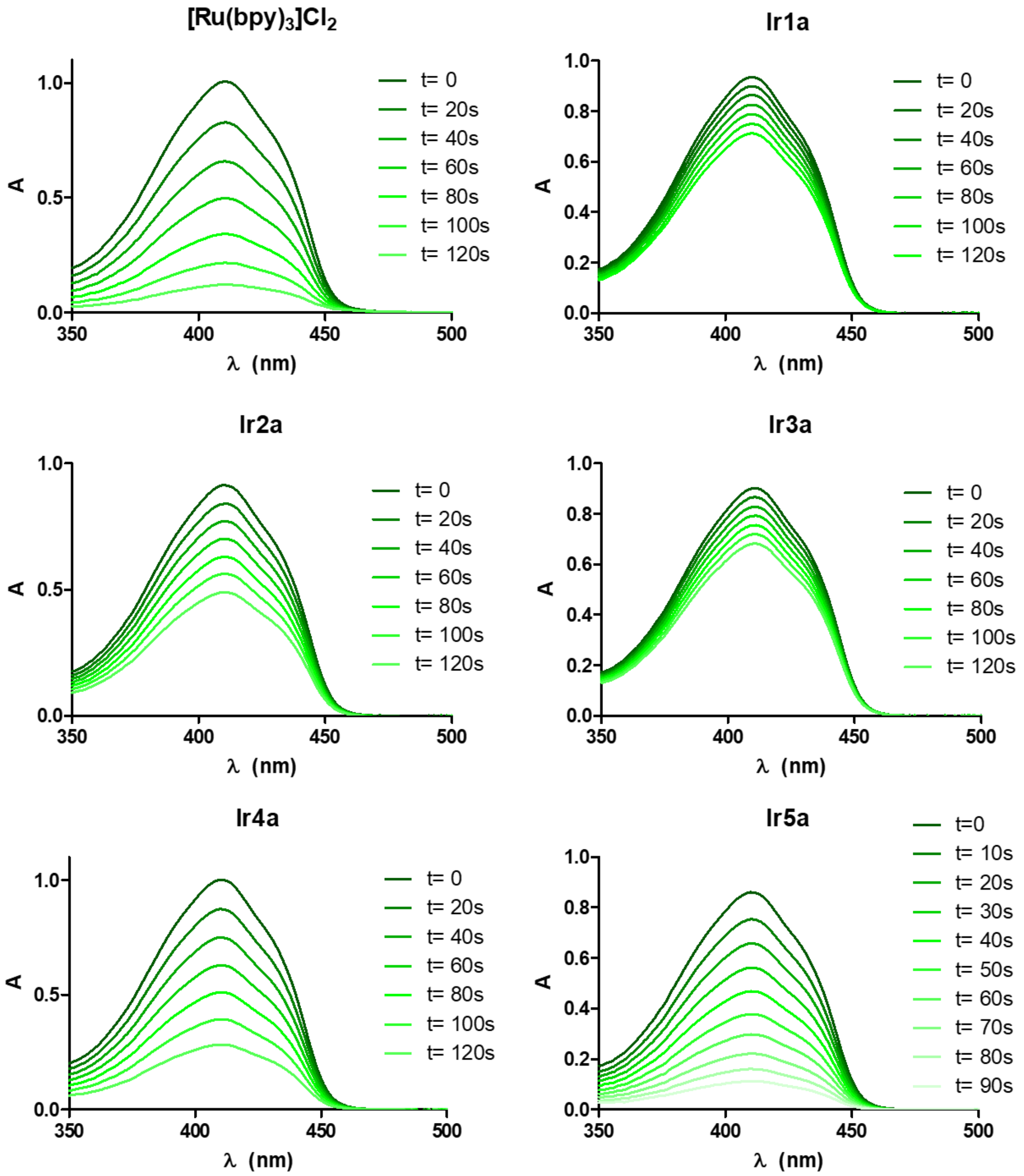

Figure S53. Absorbance decrease of DPBF (50 µM) in presence of **Ir1a-5a** complexes and the reference $[Ru(bpy)_3]Cl_2$ (50 µM) in aerated acetonitrile under green light irradiation (520 nm, 0.5 mW/cm^2).

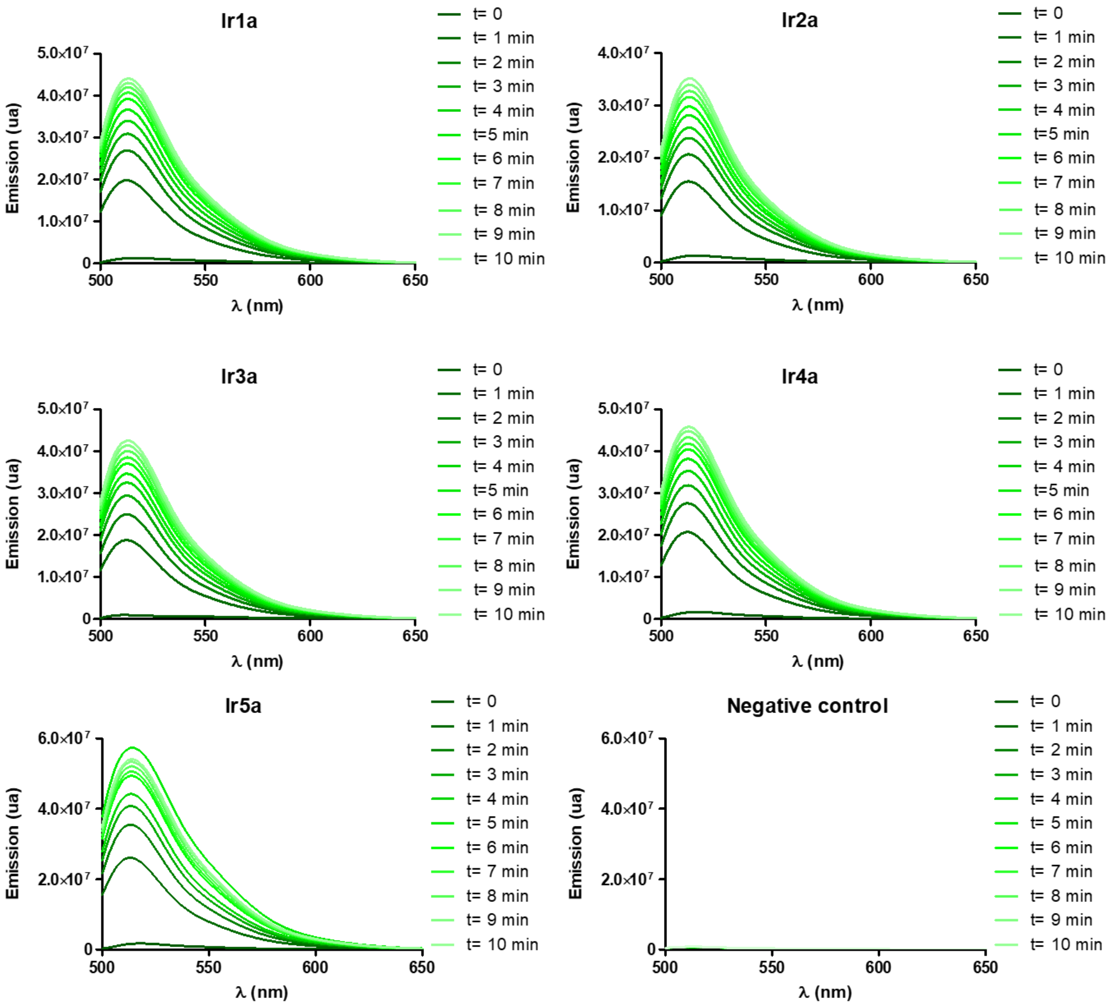

Figure S54. Increase of the fluorescence spectra emission of HPF upon photoirradiation of **Ir1a-5a** complexes at 520 nm (2.0 mW/cm^2). HPF was excited at 490 nm.

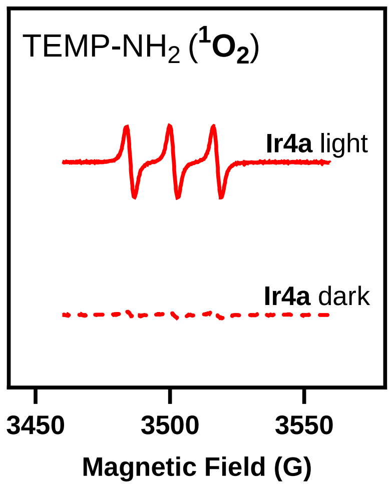

Figure S55. EPR spectra of **Ir4a** complex trapped by 4-amino-TEMP in MeOH measured in the dark and after green-light irradiation (300 s for $^1O_2$; 520 nm).

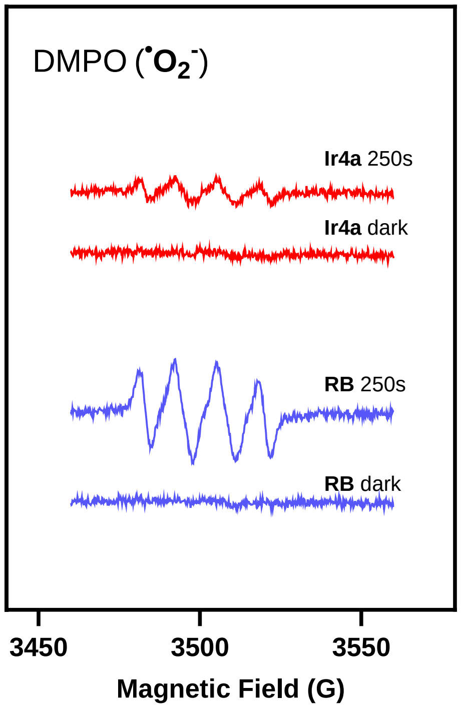

Figure S56. EPR spectra of **Ir4a** and **RB** complexes trapped by DMPO in MeOH measured in the dark and after orange-light irradiation (250 s for $^{\bullet}O_2^{-}$; 580 nm).

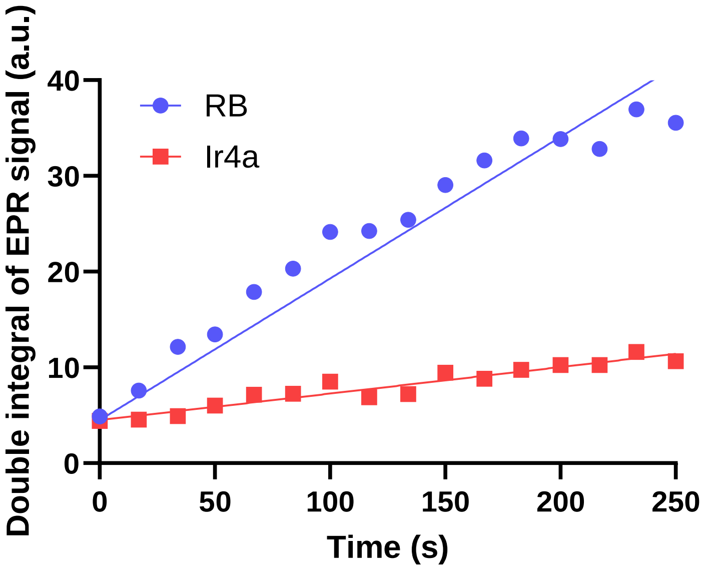

Figure S57. Second integral of the EPR signal of **Ir4a** and **RB** trapped by DMPO in MeOH, plotted as a function of time during irradiation with orange-light (300 s; 580 nm) for $^{\bullet}O_2^-$ detection.

8. Cellular uptake studies by confocal microscopy of Ir-COUBPY and Ir-bpy complexes

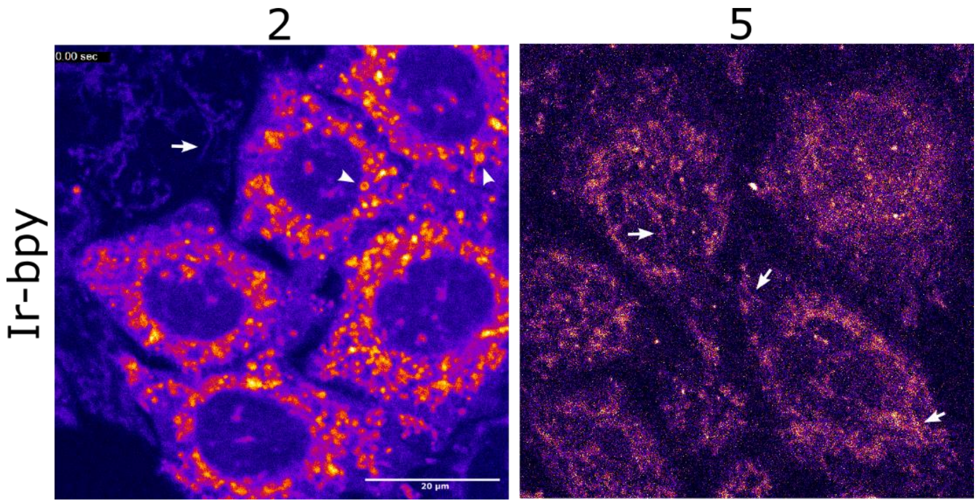

Figure S58. Cellular uptake of **Ir2b** and **Ir5b** in living HeLa cells, visualized by confocal microscopy. Images show single confocal planes of HeLa cells incubated with the compounds (20 μM) for 30 min at 37 °C. Ir-bpy complexes were excited at 405 nm, with emission detected between 500–625 nm. Fluorescence images use the Fire LUT. White arrows indicate mitochondria, and white arrowheads highlight doughnut-shaped mitochondria. Scale bar: 20 μm.

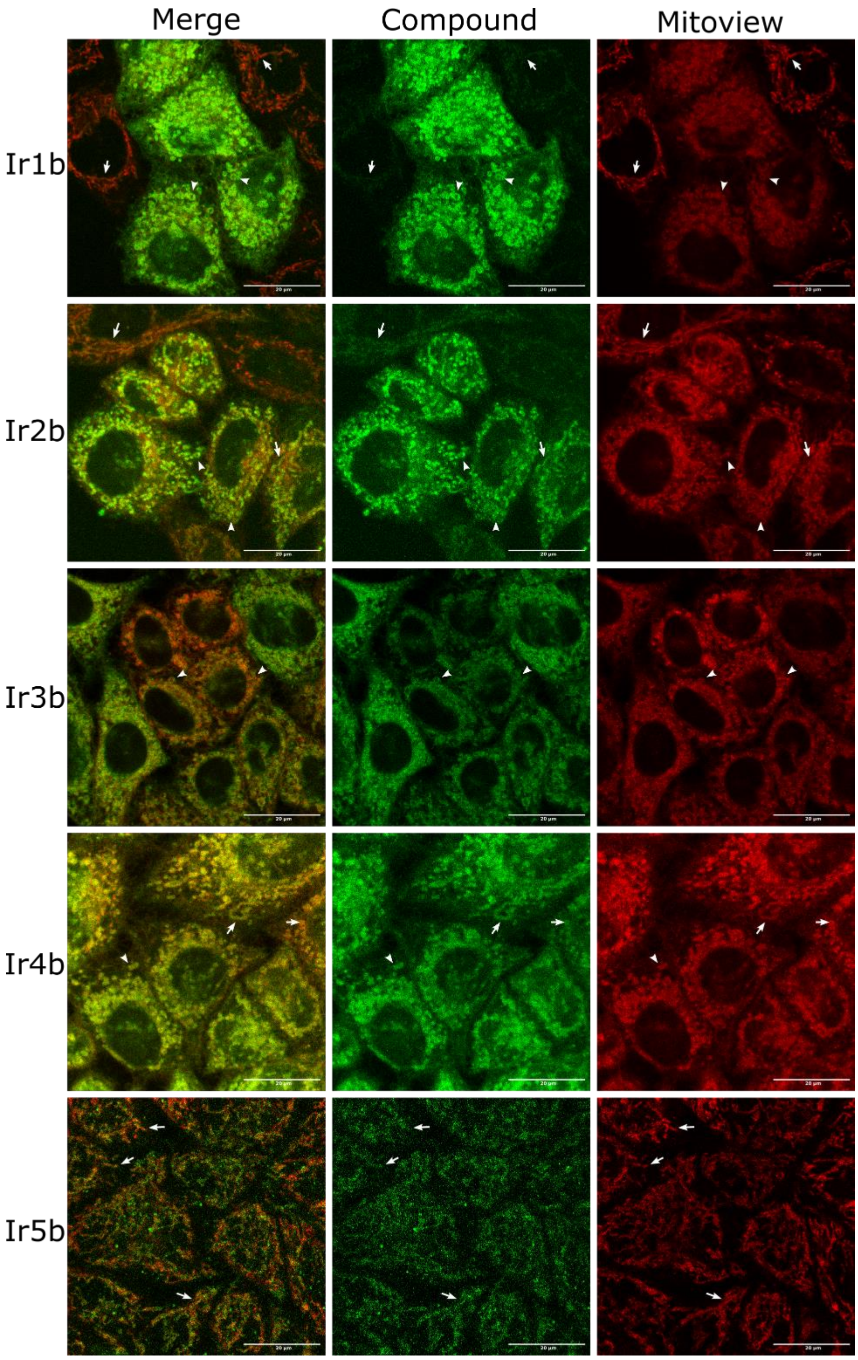

Figure S59. Co-localization of **Ir1b-Ir5b** with Mitoview 650 in HeLa cells. Images show single confocal planes of cells incubated with the compounds (5 or 20 µM, green) and Mitoview 650 (0.1 µM, red). Panels: Left, overlay of both signals; center, Ir complex fluorescence; right, Mitoview fluorescence. White arrows and arrowheads indicate colocalization of both signals in mitochondria and in doughnut-shaped mitochondria, respectively. Scale bar: 20 µm.

Table S5. Pearson's correlation coefficient (PCC) and Mander's overlap colocalization coefficients of **Ir1a**, **Ir3a** and **Ir4a** complexes with Mitoview 650. M1 corresponds to the amount of colocalization of the compound channel towards the marker channel and M2 the amount of colocalization of the marker channel towards the compound channel.

Compound	PCC	M1	M2	Nº cells
**Ir1a**	0.89	0.87	0.80	90
**Ir3a**	0.82	0.81	0.78	134
**Ir4a**	0.73	0.70	0.72	109

Table S6. Pearson's correlation coefficient (PCC) and Mander's overlap colocalization coefficients of **Ir1b-Ir5b** complexes with Mitoview 650. M1 corresponds to the amount of colocalization of the compound channel towards the marker channel and M2 the amount of colocalization of the marker channel towards the compound channel.

Compound	PCC	M1	M2	Nº cells
**Ir1b**	0.62	0.65	0.52	109
**Ir2b**	0.69	0.76	0.62	102
**Ir3b**	0.78	0.74	0.74	99
**Ir4b**	0.84	0.84	0.76	115
**Ir5b**	0.55	0.49	0.59	142

9. Partition coefficients (log P)

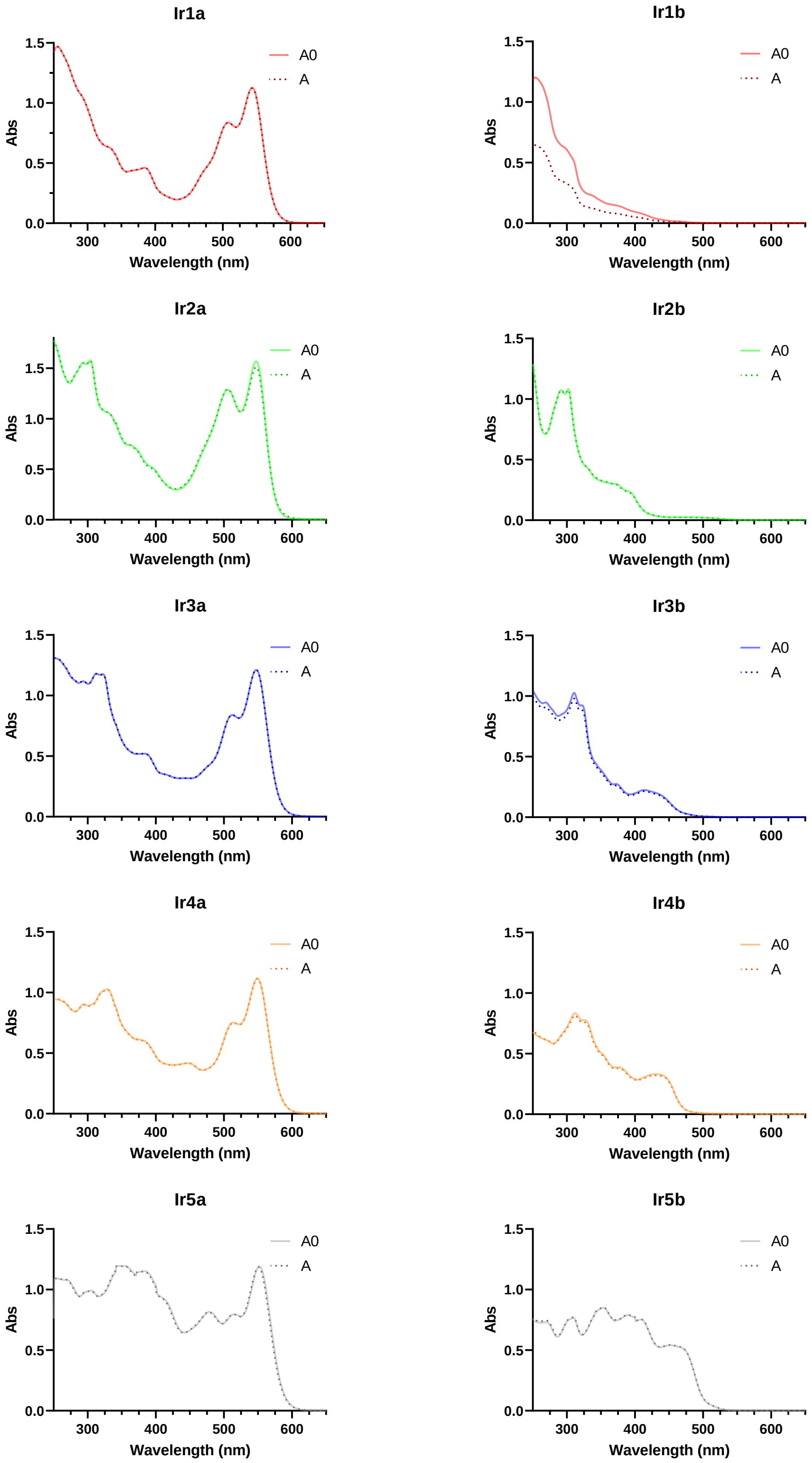

Figure S60. UV-Vis absorption spectra of the solutions used to determine the partition coefficients of the Ir–COUBPY (left panels) and Ir–bpy (right panels) complexes. Solid lines represent the initial reserved aliquots (A_0), while dotted lines correspond to the pre-equilibrated phases (A).

Table S7. Partition coefficient (log P) values of the Ir(III) complexes investigated in this study. The wavelength used for absorbance measurements of each compound is indicated.

**Ir-COUBPY**		λ_{max} (nm)	**Ir-bpy**		λ_{max} (nm)
**Ir1a**	2.47 ± 0.21	508	**Ir1b**	- 0.11 ± 0.03	338
**Ir2a**	2.51 ± 0.26	505	**Ir2b**	1.97 ± 0.34	370
**Ir3a**	2.65 ± 0.13	512	**Ir3b**	1.29 ± 0.02	414
**Ir4a**	2.48 ± 0.38	549	**Ir4b**	1.39 ± 0.19	430
**Ir5a**	2.88 ± 0.48	514	**Ir5b**	2.59 ± 0.39	450

10. Synthesis and characterization of NC-Ir4a

Table S8. Synthesis of amphiphilic cationic polymer

Reagent	Abbreviation	g	mmol	meq
2-2'-Dihydroxyethyl disulfide	DEDS	3,31	21,44	42,88
-	YMER N120	43,83	43,36	86,72
N-(3-dimethylaminopropyl)-*N,N'*-diisopropanolamine	Jeffcat DPA	3,57	16,35	32,71
Isophorone diisocyanate	IPDI	30,25	136,11	272,21
1,3-diamino-N-octadecylpropane	Genamin TAP 100D	20,99	63,98	127,96
Tetrahydrofuran	THF	125,53		

Table S9. Synthesis of **Ir4a**-loaded NCs (**NC-Ir4a)**

Reagent	mg	mmol	meq
Ir4a	8.4	7.79 μmol	-
Neobee 1053	23.3	46.20 μmol	-
IPDI	27.9	0.13	0.25
THF	1.6 mL	-	-
L-lysine	10.6	0.06	0.12
Mili-Q Water	8.1 g	-	-
Diethylenetriamine (DETA)	2.0	0.02	0.06
PP22R39	938.9	-	0.07

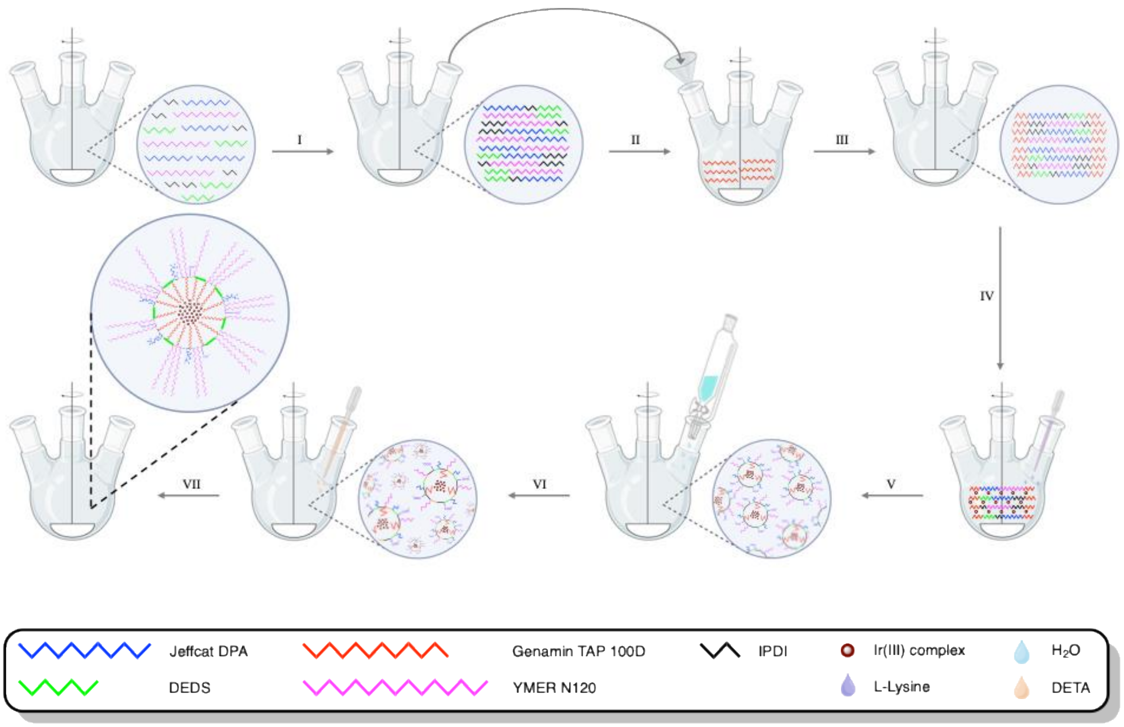

Figure S61. Schematic representation of the synthesis of the amphiphilic prepolymer (steps 1-3) and of the nanoencapsulation procedure. Abbreviations: *N*-(3-dimethylaminopropyl)-*N,N'*-diisopropanolamine (Jeffcat DPA), 2,2'-dihidroxyethyl disulfide (DEDS), 1,3-diamino-*N*-octadecylpropane (Genamin TAP 100D), isophorone diisocyanate (IPDI), diethylentriamine (DETA).

Table S10. Hydrodynamic diameter average of the nanoparticles.

Sample	Diameter ± SD (nm)
**NC-Ir4a**	11.69 ± 0.24

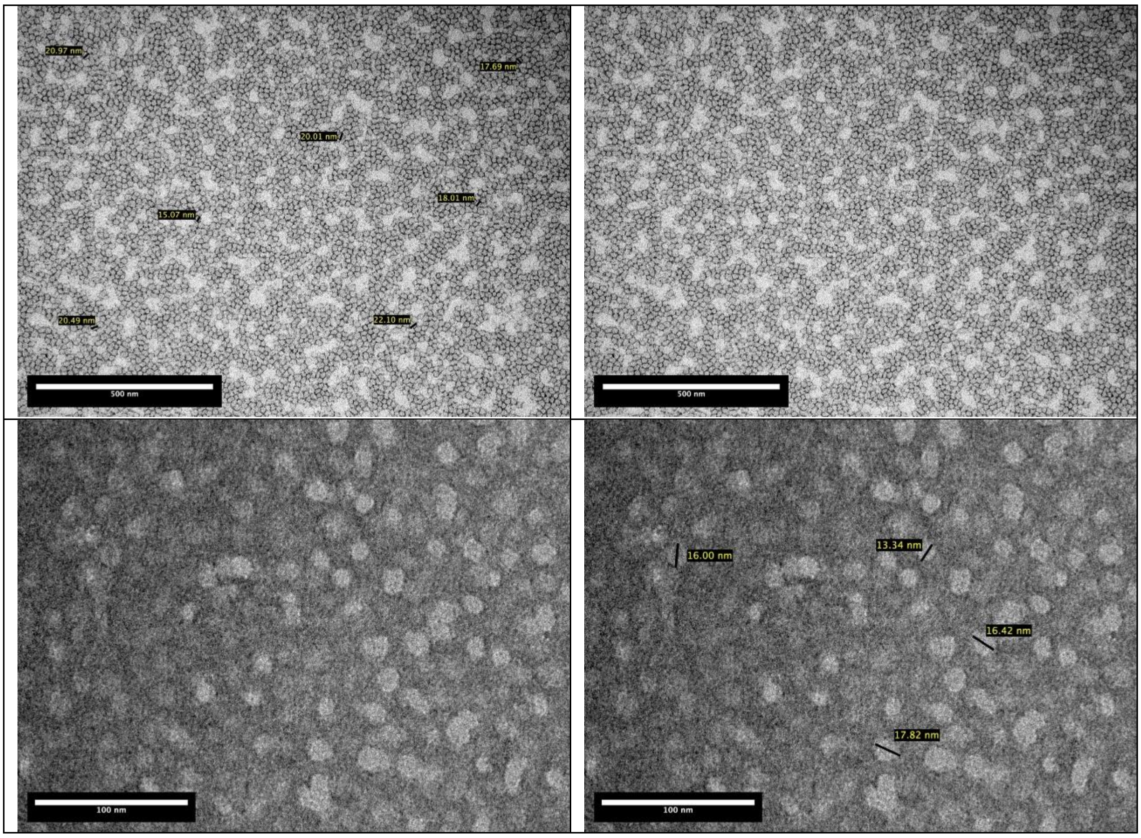

Figure S62. Particle size distribution of **NC-Ir4a** determined by TEM. Scale bars: top image, 500 nm; bottom image, 100 nm.

Table S11. Z-Potential pH dependence of **Ir4a**-loaded nanocapsules.

**Sample**	**Z-Pot ± SD (mV) at pH=6**	**Z-Pot ± SD (mV) at pH=6.5**	**Z-Pot ± SD (mV) at pH=7**	**Z-Pot ± SD (mV) at pH=7.5**
**NC-Ir4a**	15.5 ± 0.3	13.5 ± 0.6	10.2 ± 0.1	7.4 ± 0.1

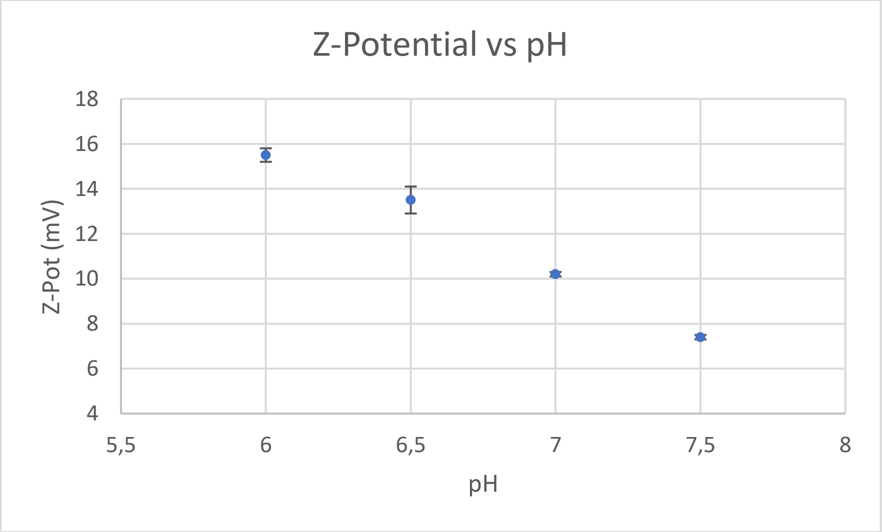

Figure S63. Z-Potential pH dependence of Ir(III)-loaded nanocapsules.

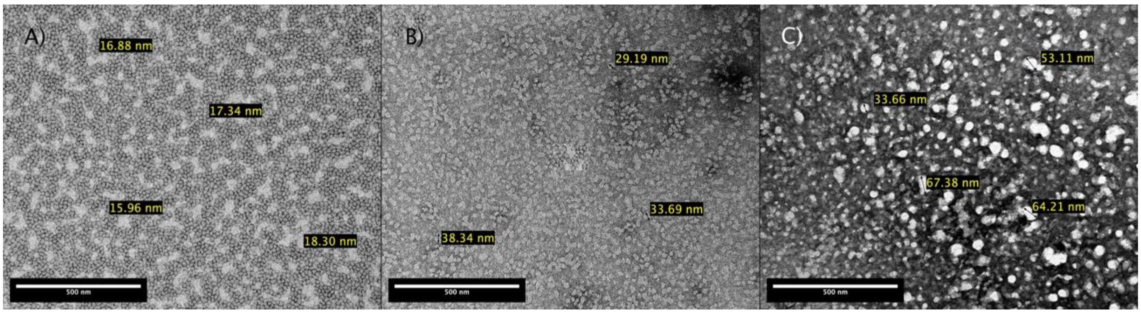

Figure S64. Selected TEM micrographs of Ir(III)-loaded nanocapsules. A) 4 mg/mL; B) incubated 4 mg/mL in 25 mM GSH-supplemented PBS for 24h and C) incubated 4 mg/mL in 25 mM GSH-supplemented PBS for 48 h.

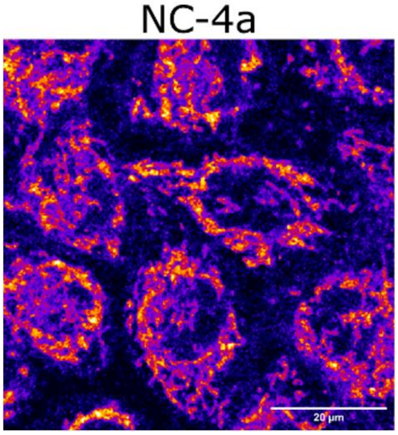

Figure S65. Cellular uptake of nanoformulation **NC-Ir4a** in living HeLa cells. Images show single confocal plane of HeLa cells incubated with the nanoencapsulated compound (20 µM) for 30 min at 37 °C. Excitation was carried out at 514 nm, with emission detected between 520–640 nm. White arrows indicate mitochondria, and white arrowheads highlight doughnut-shaped mitochondria. Lookup table (LUT): Fire. Scale bar: 20 µm.

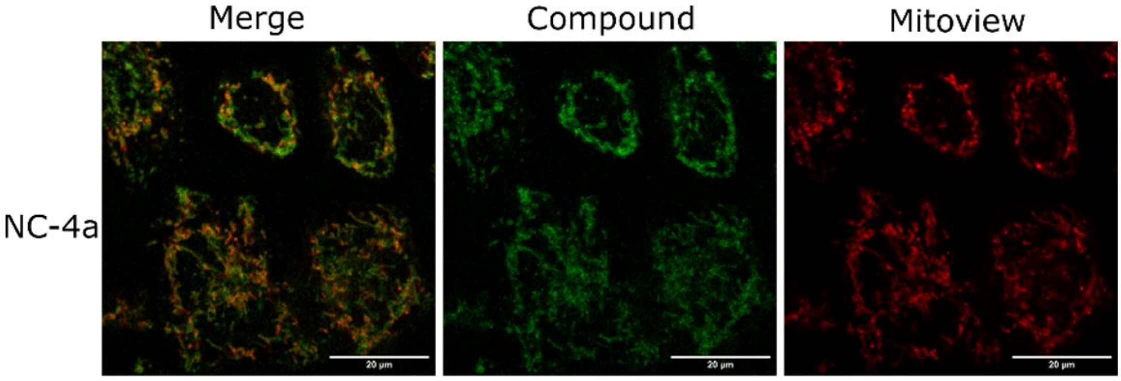

Figure S66. Co-localization of nanoformulation **NC-Ir4a** with Mitoview 650 in HeLa cells. Images show single confocal planes of cells incubated with the nanoencapsulated compounds (20 µM, green) and Mitoview 650 (0.1 µM, red). Panels: Left, overlay of both signals; center, Ir complex fluorescence; right, Mitoview fluorescence. Excitation was carried out at 514 nm, with emission detected between 520–640 nm. Mitoview was excited at 633 nm, with emission detected between 670–750 nm. White arrows indicate mitochondria, and white arrowheads highlight doughnut-shaped mitochondria. Scale bar: 20 µm.

Table S12. Pearson's correlation coefficient and Mander's overlap colocalization coefficients of the nanoencapsulated Ir-COUBPY complexe **Ir4a** with Mitoview 650. M1 corresponds to the percentage of colocalization of the compound channel towards the marker channel and M2 the percentage of colocalization of the marker channel towards the compound channel.

Compound	PCC	M1	M2	Nº cells
**NC-4a**	0.72	0.62	0.74	162

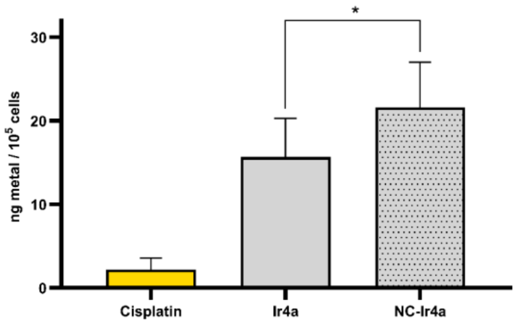

Figure S67. Intracellular accumulation of Ir in A375 cells incubated with Ir4a and NC-Ir4a (10 µM) for 1 h. The uptake of Pt (yellow bar) was set as the positive control. Data for intracellular Ir concentration presented as the mean ± SD from two independent tests. Statistical significance was determined using a two-tailed Student’s t test (*p < 0.05, **p ≤ 0.01 or ***p ≤ 0.001).

11. Biological assays

Table S13. (Photo)cytotoxicity of Ir-COUBPY complexes towards A375 and HeLa Cancer Cells Expressed as IC_{50} Values (μM).[a]

	A375				HeLa			
	IC_{50} values (μM)[a]			PI[b]	IC_{50} values (μM)[a]			PI[b]
**Compound**	**Dark**	**Green**	**Red**	**Green**	**Dark**	**Green**	**Red**	**Green**
**Ir1a**	0.103 ± 0.006	0.009 ± 0.003	0.028 ± 0.003	11.4	0.111 ± 0.007	0.010 ± 0.002	0.026 ± 0.003	11.1
**Ir2a**	> 100	0.5 ± 0.2	7.7 ± 08	> 200	> 100	0.8 ± 0.2	12.0 ± 1.9	> 125
**Ir3a**	3.3 ± 1.0	0.097 ± 0.017	0.588 ± 0.049	34.0	1.6 ± 0.3	0.085 ± 0.015	0.131 ± 0.013	18.8
**Ir4a**	4.8 ± 0.5	0.075 ± 0.007	0.123 ± 0.007	64.0	2.1 ± 0.5	0.095 ± 0.012	0.127 ± 0.014	22.1
**Ir5a**	> 100	9.3 ± 0.9	10.9 ± 2.4	> 10.8	> 100	6.4 ± 0.7	6.4 ± 0.7	> 15.6
**NC-Ir4a**	17.6 ± 4.7	0.098 ± 0.025	0.290 ± 0.031	179.6	17.3 ± 4.2	0.112 ± 0.018	0.687 ± 0.118	154.5

[a]Experimental conditions: Cells were incubated for 1 h at 37 °C, followed by either 1 h in the dark or irradiation under green (520 nm, 1.5 mW/cm^2) or red (620 nm, 15 mW/cm^2) light. Cell viability was determined after 46 h using the MTT assay. [b]Phototherapeutic index (PI) = IC_{50}(dark)/IC_{50}(light).

Table S14. (Photo)cytotoxicity of Ir-bpy complexes towards A375 and HeLa Cancer Cells Expressed as IC_{50} Values (μM).[a]

	A375			HeLa		
**Compound**	**IC_{50} dark (μM)[a]**	**IC_{50} light (μM)[a]**	**PI[b]**	**IC_{50} dark (μM)[a]**	**IC_{50} light (μM)[a]**	**PI[b]**
**Ir1b**	1.8 ± 0.4	2.3 ± 0.5	0.8	19.7 ± 5.4	13.6 ± 4.5	1.4
**Ir2b**	3.4 ± 0.7	0.98 ± 0.16	3.7	> 100	19.4 ± 9.2	> 5.2
**Ir3b**	0.30 ± 0.09	0.082 ± 0.010	3.7	1.9 ± 0.4	0.300 ± 0.055	6.3
**Ir4b**	0.50 ± 0.06	0.093 ± 0.007	5.4	2.5 ± 0.2	0.146 ± 0.011	17.1
**Ir5b**	15.3 ± 3.2	3.9 ± 0.2	3.9	17.5 ± 4.0	2.7 ± 0.3	6.5

[a]Experimental conditions: Cells were incubated for 1 h at 37 °C, followed by either 1 h in the dark or irradiation under green light (520 nm, 1.5 mW/cm^2). Cell viability was determined after 46 h using the MTT assay. [b]Phototherapeutic index (PI) = IC_{50}(dark)/IC_{50}(light).

Table S15. (Photo)cytotoxicity of Ir-COUBPY and Ir-bpy complexes towards A375 Cancer Cells Expressed as IC_{50} Values (μM), normalized by Ir accumulation of **Ir1a** according to ICP-MS analysis (Figure 7).

		Normalized IC_{50} values (μM)				Normalized IC_{50} values (μM)	
**Ir-COUBPY**	**Relative accumulation**	**Dark**	**Green**	**Ir-bpy**	**Relative accumulation**	**Dark**	**Green**
**Ir1a**	1	0.103	0.009	**Ir1b**	0.87	1.57	2.00
**Ir2a**	0.13	> 13	0.065	**Ir2b**	0.09	0.306	0.088
**Ir3a**	0.65	2.15	0.063	**Ir3b**	0.27	0.081	0.022
**Ir4a**	0.31	1.49	0.023	**Ir4b**	0.8	0.4	0.074
**Ir5a**	0.054	> 5.4	0.502	**Ir5b**	0.056	0.857	0.218

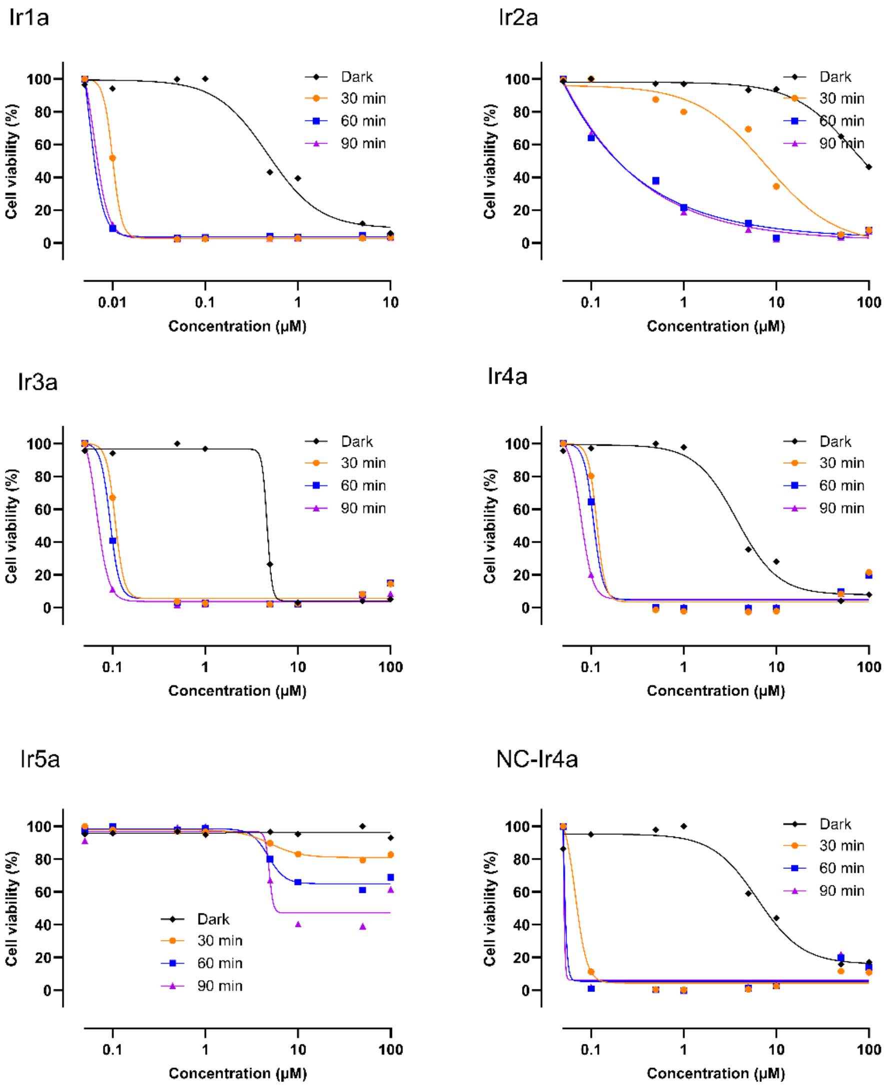

Figure S68. Dose–response photocytotoxicity of Ir-COUBPY complexes under green-light irradiation with varying exposure times (30, 60, and 90 min). A375 cells were treated with the indicated compounds for 1 h, irradiated with green light (λ = 520 nm, 1.5 mW cm^2) for the specified duration and allowed to recover for 48 h before viability assessment.

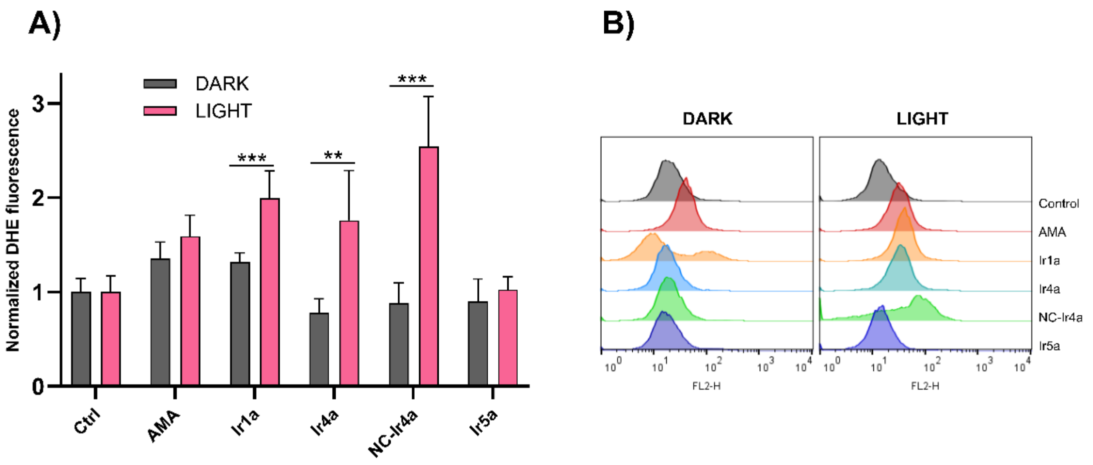

Figure S69. (A) Normalized intracellular ROS generation in A375 cells treated with the indicated compounds for 1 h, followed by 1 h of green light irradiation. Cells were stained with DHE and analyzed by flow cytometry (λ_{ex} = 488 nm and λ_{em} = 605 nm). Statistical significance was determined using an independent unpaired t-test (*p<0.05, **p<0.01, ***p<0.001). (B) Overlay histogram of DHE fluorescence intensity (FL-2 channel) in A375 cells under dark and green light conditions after treatment with the indicated compounds measured by flow cytometry (λ_{ex} = 488 nm and λ_{em} = 605 nm).

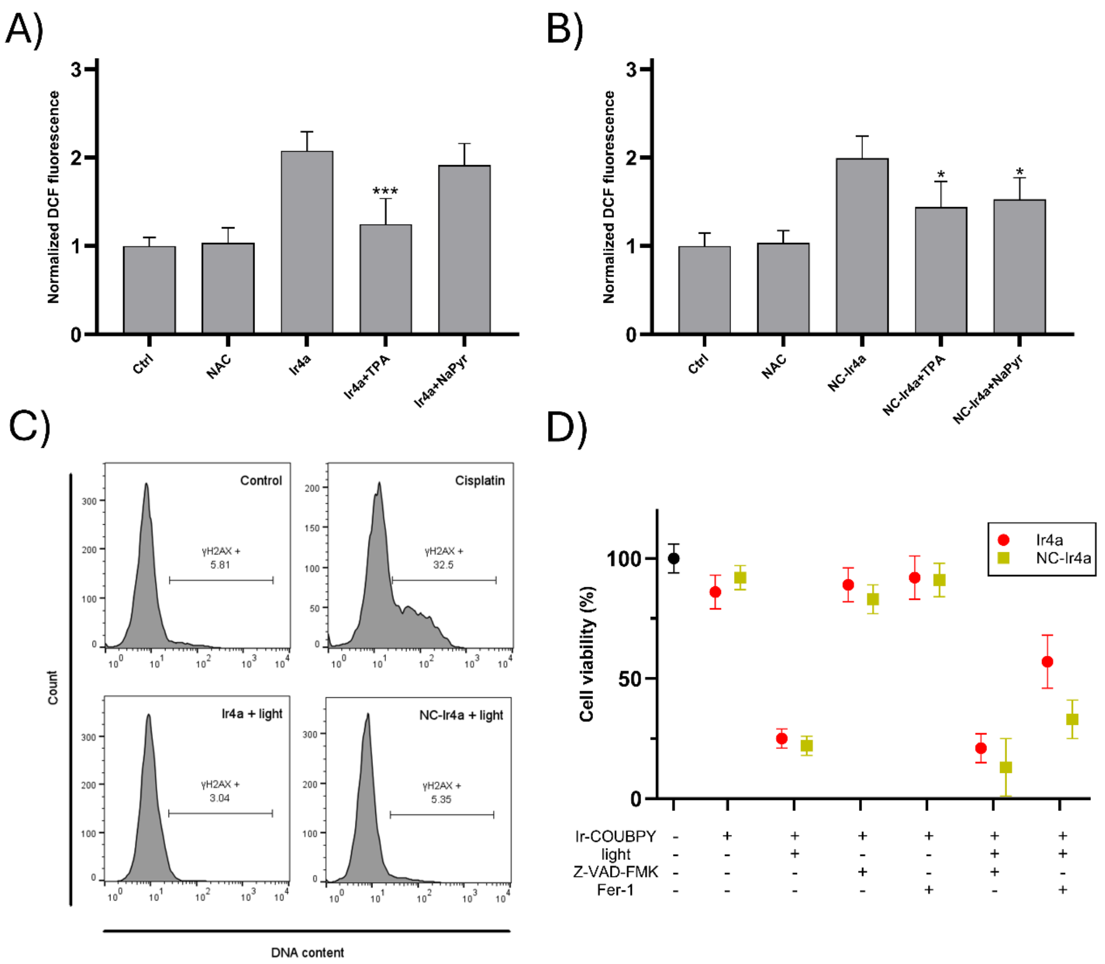

Figure S70. ROS levels in A375 cells following green light irradiation after treatment with 250 nM of (A) **Ir4a** and (B) **NC-Ir4a**, alone or in combination with terephthalic acid (TPA, 20 μM) and sodium pyruvate (NaPyr, 10 μM). Cells were incubated for 1 h in the dark, then irradiated with green light for 1 h, stained with DCFH-DA for 30 minutes at 37°C. Statistical significance was assessed using one-way ANOVA, comparing treatments with conjugates alone while significance levels indicated as *p < 0.05, **p < 0.01, and ***p < 0.001. Data are presented as mean ± SD (n = 2 replicates). (C) Cell viability after a 1 h treatment with 250 nM **Ir4a** or **NC-Ir4a**, co-treated with 10 μM Z-VAD-FMK or 10 μM Fer-1, followed by 1h of green light irradiation. Cells maintained in the dark served as the control. (D) Effect of **Ir4a** and **NC-Ir4a** on DNA damage, assessed by changes in γH2AX staining detected in the FL1-H channel, after 1 h treatment followed by 1 h of green light irradiation. Cisplatin was used as a positive control for DNA damage induction.

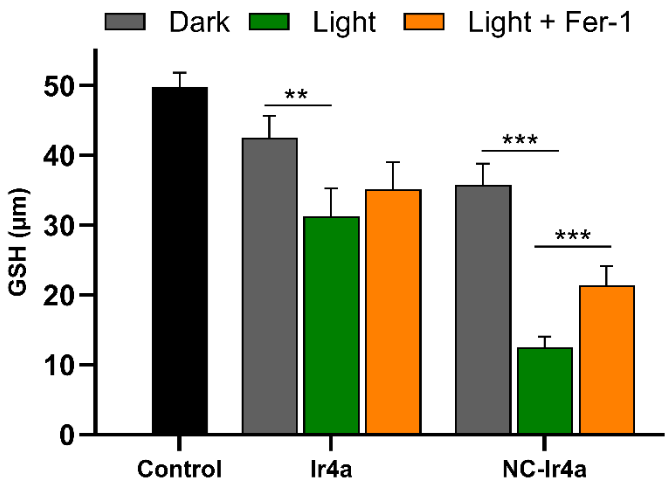

Figure S71. Reduced GSH level (µM) measured 4 h after green-light irradiation in A375 cells. Bars show mean ± SD (n = 3 independent experiments). One-way ANOVA was used for significance testing (*p < 0.05; **p < 0.01; **p < 0.001).

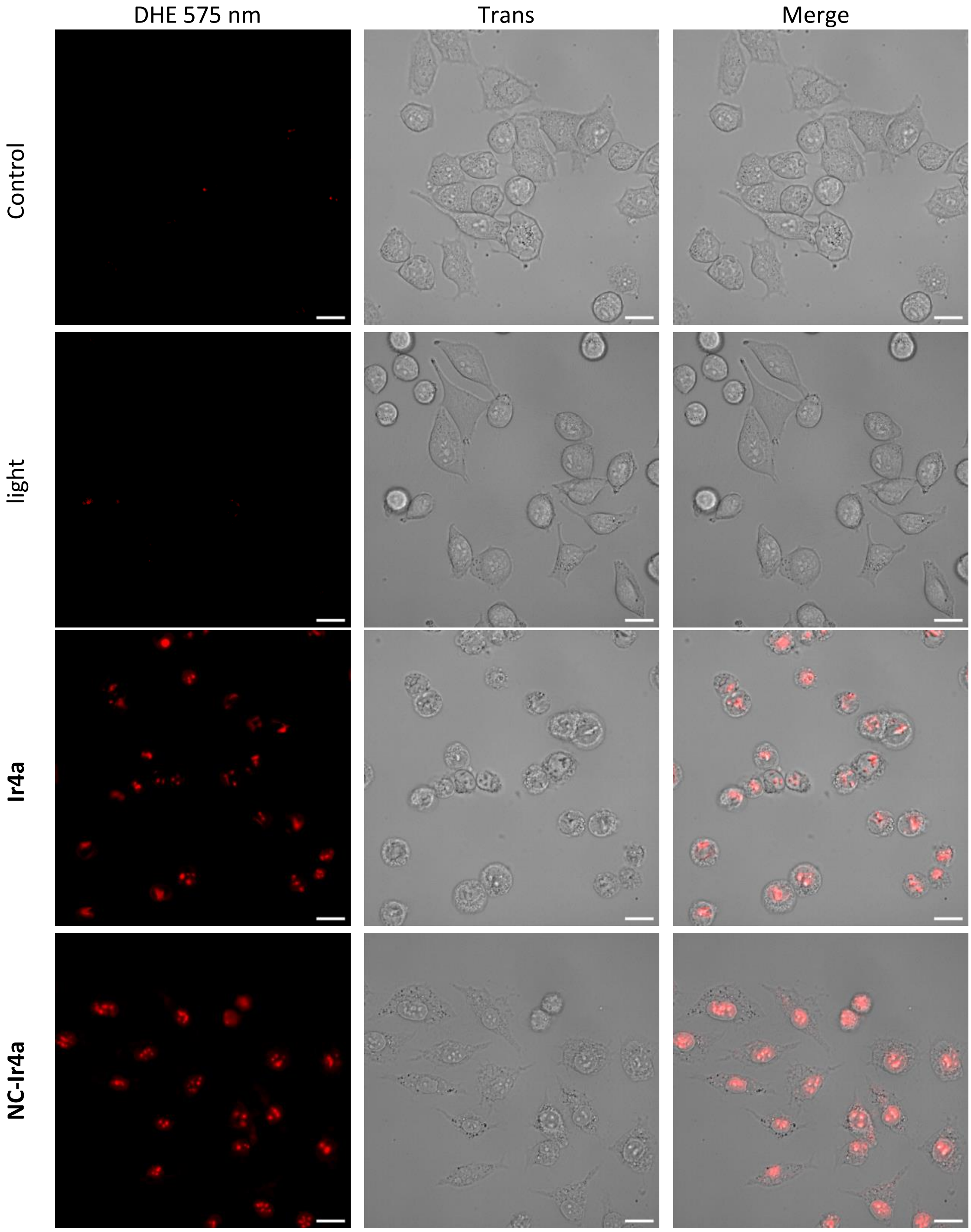

Figure S72. Widefield fluorescence microscopy images of A375 cells treated with **Ir4a** and **NC-Ir4a** (250 nM, 1 h) under light exposure and co-stained with DHE (λ_{ex} = 530 nm, λ_{em} = 605 ± 30 nm). Treatments with unloaded nano-capsules and light were used as controls. Scale bar: 20 μm.

A) B) C)

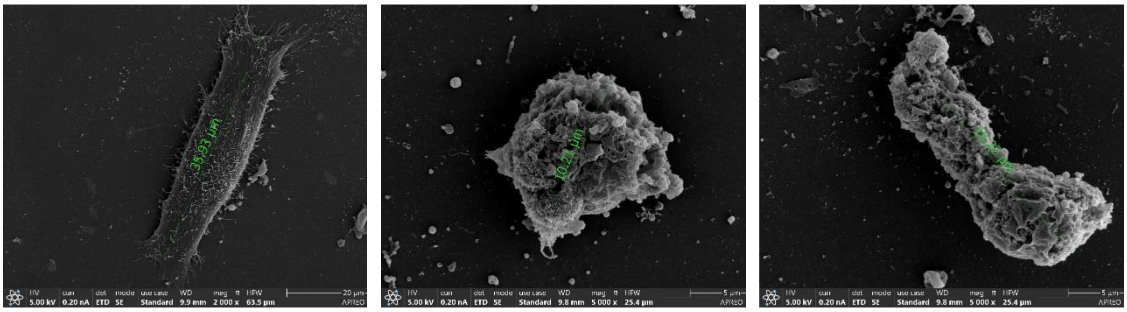

Figure S73. (A) Control, (B) cisplatin, (C) **Ir4a**-treated A375 cells showing differences in cell length observed under FE-SEM. Treated cells exhibit a reduction in length due to apoptosis- and ferroptosis-induced shrinkage. Magnification: 2000× (control) and 5000× (treated cells).

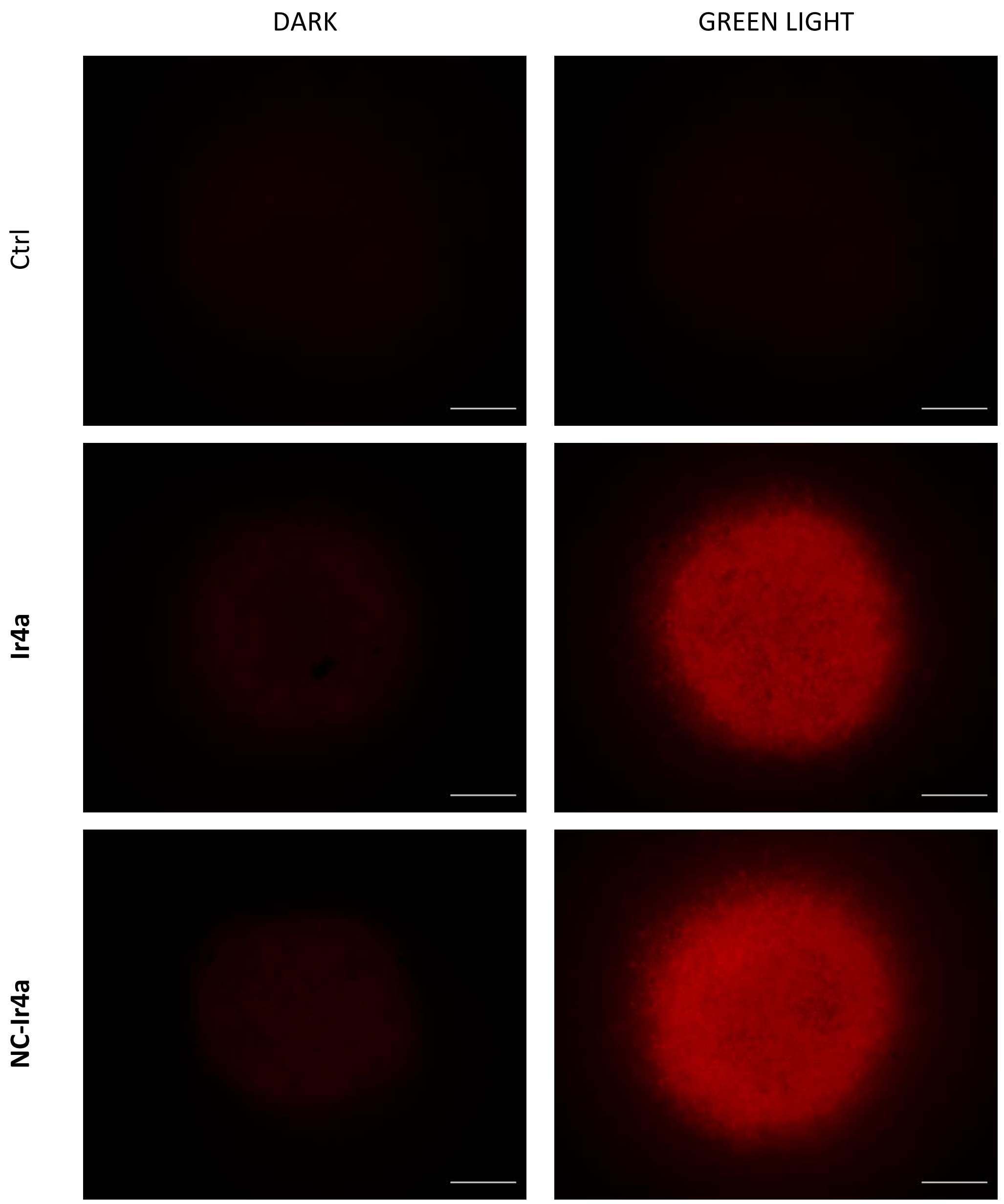

Figure S74. Fluorescence microscopy analysis of A375 multicellular tumor spheroids (MCTS) stained with dihydroethidium (DHE, 10 μM) to assess reactive oxygen species (ROS) generation. Spheroids were treated with **Ir4a** or **NC-Ir4a** (2.5 μM) for 1 hour, followed by 1-hour green light irradiation and 4 days of incubation, with a second treatment administered on day 2. Identical treatments under dark conditions served as controls. Scale bar: 200 μm.

12. References

(1) Abad-Montero, D.; Gandioso, A.; Izquierdo-Garcia, E.; Chumillas, S.; Rovira, A.; Bosch, M.; Jorda-Redondo, M.; Castaño, D.; Bonelli, J.; Novikov, V. V.; Deya, A.; Hernandez, J. L.; Galino, J.; Alberto, M. E.; Frances-Monerris, A.; Nonell, S.; Gasser, G.; Marchán, V. Ruthenium(II) Polypyridyl Complexes Containing COUBPY Ligands as Potent Photosensitizers for the Efficient Phototherapy of Hypoxic Tumors. *J Am Chem Soc*, **2025**, *147* (9), 7360–7376.

(2) Yellol, J.; Perez, S. A.; Yellol, G.; Zajac, J.; Donaire, A.; Vigueras, G.; Novohradsky, V.; Janiak, C.; Brabec, V.; Ruiz, J. Highly potent extranuclear-targeted luminescent iridium(iii) antitumor agents containing benzimidazole-based ligands with a handle for functionalization. *Chem Commun*, **2016**, *52* (98), 14165–14168.

(3) Novohradsky, V.; Vigueras, G.; Pracharova, J.; Cutillas, N.; Janiak, C.; Kostrhunova, H.; Brabec, V.; Ruiz, J.; Kasparkova, J. Molecular superoxide radical photogeneration in cancer cells by dipyridophenazine iridium (III) complexes. *Inorg Chem Fron*, **2019**, *6* (9), 2500–2513.

(4) Gandioso, A.; Izquierdo-Garcia, E.; Mesdom, P.; Arnoux, P.; Demeubayeva, N.; Burckel, P.; Saubamea, B.; Bosch, M.; Frochot, C.; Marchán, V.; Gasser, G. Ru(II)-Cyanine Complexes as Promising Photodynamic Photosensitizers for the Treatment of Hypoxic Tumours with Highly Penetrating 770 nm Near-Infrared Light. *Chem Eur J*, **2023**, *29* (61), e202301742.

(5) Schindelin, J.; Arganda-Carreras, I.; Frise, E.; Kaynig, V.; Longair, M.; Pietzsch, T.; Preibisch, S.; Rueden, C.; Saalfeld, S.; Schmid, B.; Tinevez, J. Y.; White, D. J.; Hartenstein, V.; Eliceiri, K.; Tomancak, P.; Cardona, A. Fiji: an open-source platform for biological-image analysis. *Nat Methods*, **2012**, *9* (7), 676–682.
